# hnRNP K Coordinates Transcriptional Silencing by SETDB1 in Embryonic Stem Cells

**DOI:** 10.1371/journal.pgen.1004933

**Published:** 2015-01-22

**Authors:** Peter J. Thompson, Vered Dulberg, Kyung-Mee Moon, Leonard J. Foster, Carol Chen, Mohammad M. Karimi, Matthew C. Lorincz

**Affiliations:** 1 Life Sciences Institute, Department of Medical Genetics, University of British Columbia, Vancouver, British Columbia, Canada; 2 Centre for High-Throughput Biology, University of British Columbia, Vancouver, British Columbia, Canada; 3 Biomedical Research Centre, University of British Columbia, Vancouver, British Columbia, Canada; Stanford University School of Medicine, United States of America

## Abstract

Retrotransposition of endogenous retroviruses (ERVs) poses a substantial threat to genome stability. Transcriptional silencing of a subset of these parasitic elements in early mouse embryonic and germ cell development is dependent upon the lysine methyltransferase SETDB1, which deposits H3K9 trimethylation (H3K9me3) and the co-repressor KAP1, which binds SETDB1 when SUMOylated. Here we identified the transcription co-factor hnRNP K as a novel binding partner of the SETDB1/KAP1 complex in mouse embryonic stem cells (mESCs) and show that hnRNP K is required for ERV silencing. RNAi-mediated knockdown of hnRNP K led to depletion of H3K9me3 at ERVs, concomitant with de-repression of proviral reporter constructs and specific ERV subfamilies, as well as a cohort of germline-specific genes directly targeted by SETDB1. While hnRNP K recruitment to ERVs is dependent upon KAP1, SETDB1 binding at these elements requires hnRNP K. Furthermore, an intact SUMO conjugation pathway is necessary for SETDB1 recruitment to proviral chromatin and depletion of hnRNP K resulted in reduced SUMOylation at ERVs. Taken together, these findings reveal a novel regulatory hierarchy governing SETDB1 recruitment and in turn, transcriptional silencing in mESCs.

## Author Summary

Retroelements, including endogenous retroviruses (ERVs), pose a significant threat to genome stability. In mouse embryonic stem (ES) cells, the enzyme SETDB1 safeguards the genome against transcription of specific ERVs by depositing a repressive mark H3K9 trimethylation (H3K9me3). Although SETDB1 is recruited to ERVs by its binding partner KAP1, the molecular basis of this silencing pathway is not clear. Using biochemical and genetic approaches, we identified hnRNP K as a novel component of this silencing pathway that facilitates the recruitment of SETDB1 to ERVs to promote their repression. HnRNP K binds to ERV sequences via KAP1 and subsequently promotes SETDB1 binding. Together, our results reveal a novel function for hnRNP K in transcriptional silencing of ERVs and demonstrate a new regulatory mechanism governing the deposition of H3K9me3 by SETDB1 in ES cells.

## Introduction

Long terminal repeat (LTR) retrotransposons, also called endogenous retroviruses (ERVs), are the relics of ancient and more recent germline retroviral integrations, comprising ~8–10% of the mouse and human genomes, respectively [1]. *De novo* retrotransposition of these parasitic elements is responsible for ~10% of spontaneous mutations in mice [2]. Among the remaining transcriptionally competent ERVs in the mouse genome, many class I Moloney murine leukemia virus (MLV) and class II intracisternal A-type particle (IAP) and MusD elements are transiently expressed and subsequently silenced in the early embryo [3]. Distinct epigenetic mechanisms cooperate to maintain ERV silencing including DNA methylation, covalent histone modifications, chromatin remodelling and non-coding RNAs [4].

Although DNA methylation suppresses ERV transcription in differentiated somatic cells [5], pluripotent stem cell lines derived from the inner cell mass of the blastocyst, such as murine embryonic stem cells (mESCs) utilize a DNA methylation-independent pathway to maintain ERV silencing [6]. Key effectors in this silencing pathway are the conserved Krüppel-associated box zinc finger proteins (KRAB-ZFPs), the largest family of C2H2 zinc finger transcription factors in vertebrate genomes [7]. Earlier experiments utilizing the MLV-based retroviral vectors harbouring a proline tRNA primer binding site (PBS^Pro^) revealed that KRAB-ZFPs bind to specific proviral sequences such as the PBS, to direct the recruitment of a large silencing complex that includes the obligate co-repressor KAP1 (also called TRIM28/TIF1β) [8, 9] and the lysine methyltransferase SETDB1 (also called ESET/KMT1E), which deposits H3K9me3 to maintain a repressive chromatin state [10, 11]. Interestingly, the KRAB-ZFP/KAP1 pathway also functions to protect the human genome against retroviral activity [12], indicating that this silencing pathway is conserved in primates. Although prototypical KRAB-ZFP candidates for this pathway have been identified, such as ZFP809 and ZFP819 [9, 13], it remains unclear whether PBS binding is a general property of most KRAB-ZFPs or only a select few. Consistent with observations that PBS sequences alone are insufficient to confer SETDB1/KAP1-mediated silencing [14], the transcription factor YY1 was shown to be required for silencing of the newly integrated MLV-based retroviruses in F9 embryonal carcinoma cells and mESCs [13], revealing that additional sequence-specific factors may collaborate with KRAB-ZFPs. In addition, KAP1 is apparently recruited to IAP elements via sequences in the 5’UTR downstream of the PBS [14].

In mESCs but not embryonic fibroblasts, both class I and II ERVs and newly integrated MLV-based retroviral vectors are marked with H3K9me3 by a SETDB1/KAP1-containing complex [11]. During DNA methylation reprogramming in E13.5 primordial germ cells (PGCs) ERVs are also marked by H3K9me3 and are silenced in a SETDB1-dependent manner [15]. Conditional knockout of *Setdb1* in undifferentiated mESCs or E13.5 PGCs abolishes H3K9me3 at ERVs and leads to reduced levels of DNA methylation and increased 5-hydroxymethylation [16], concomitant with pervasive de-repression of distinct class I and II ERV families including MLV, IAP, MMERVK10C and MusD elements [11, 15, 17]. A similar phenotype is apparent upon deletion of *Kap1* in mESCs [14]. Indeed, KAP1 is required for SETDB1 recruitment, since depletion of KAP1 leads to a loss of SETDB1 binding and H3K9me3 at ERVs and newly integrated MLV-based vectors [11, 14]. The Small ubiquitin-like modifier (SUMO) paralogue SUMO1 is conjugated to KAP1 via the autocatalytic SUMO E3 ligase activity of the plant homeodomain (PHD) zinc finger towards the bromodomain at the major lysine acceptor sites K554, K779 and K804 to direct SETDB1 recruitment and H3K9 methylation [18, 19]. However, the role of SUMOylation in SETDB1-mediated repression of ERVs and the involvement of additional factors in SUMO-dependent SETDB1 targeting have not been addressed.

Here, we identified the RNA-binding protein and transcription co-factor heterogeneous nuclear ribonucleoprotein K (hnRNP K) as a novel binding partner of the SETDB1/KAP1 complex in mESCs. Depletion of hnRNP K in these cells leads to a reduction of H3K9me3 and de-repression of class I and II ERVs, proviral reporter constructs and a cohort of germline-specific genes targeted by SETDB1. Strikingly, hnRNP K is required for SETDB1 but not KAP1 recruitment through its influence on SUMOylation levels at ERV chromatin. Taken together, our data reveal a novel RNA-independent role for hnRNP K in regulating recruitment of SETDB1 to KAP1-bound targets and in turn H3K9me3-dependent transcriptional repression in mESCs.

## Results

### hnRNP K interacts with the SETDB1/KAP1 complex in mESCs

**Figure 1:**
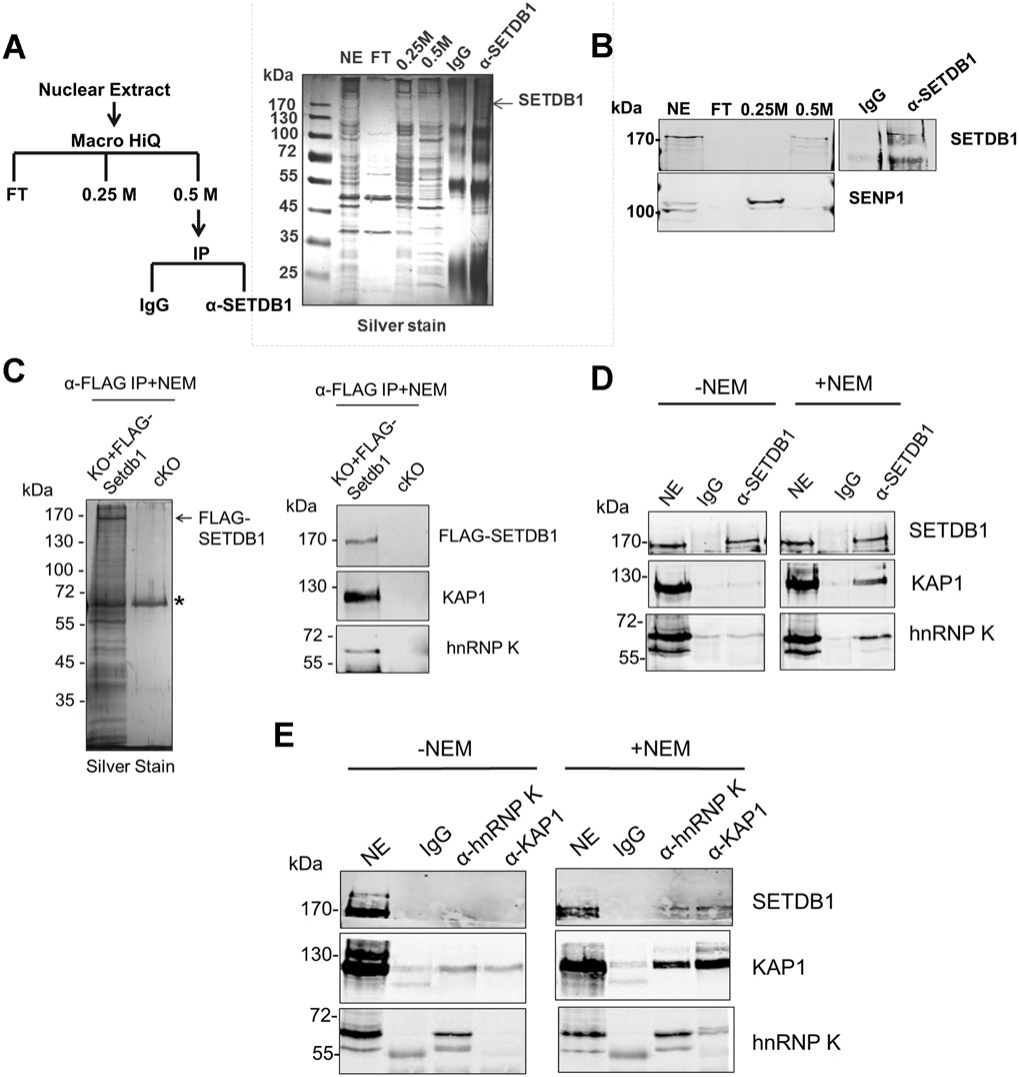
hnRNP K is associated with SETDB1 and KAP1 in mESCs. (A) IP scheme to identify SUMO-dependent binding partners of SETDB1 and silver stained gel showing protein content of the indicated fractions. The nuclear extract input (NE), negative control IP (IgG), SETDB1 IP (α-SETDB1), and the flow-through (FT), 0.25 M KCl and 0.5 M KCl fractions from the anionic column are shown. (B) Western blot of SETDB1 and SENP1 in the indicated fractions and IP. (C) Silver stained gel of FLAG-SETDB1 IP and western blot of FLAG-SETDB1, KAP1 and hnRNP K in immunopurified FLAG-SETDB1 complexes isolated from tamoxifen-induced *Setdb1* KO mESCs stably expressing 3XFLAG-*Setdb1* (KO+FLAG-Setdb1) or negative control uninduced *Setdb1* conditional KO cells (cKO). Complexes were immunoprecipitated with FLAG antibodies and specifically eluted with 3XFLAG peptide. Asterisk marks a non-specific band. Cropped band at bottom of hnRNP K blot is IgG heavy chain (~55 kDa). (D) Co-IP assay of endogenous KAP1 and hnRNP K with SETDB1 from mESC nuclear extracts in the absence or presence of 10 mM SENP inhibitor (NEM). Note that hnRNP K is detected in mESCs as two bands at ~65 kDa and ~60 kDa, the larger of which represents full-length hnRNP K and the smaller is a splicing isoform hnRNP J, also produced from the *Hnrnpk* gene [77]. The hnRNP K isoform is associated with SETDB1. ‘NE’ represents ~10% of nuclear extract input and ‘IgG’ is the negative control IP. (E) Co-IP assay of endogenous SETDB1 with KAP1 or hnRNP K from mESC nuclear extracts in the presence or absence of NEM, as in (D).

To identify novel factors involved in SETDB1-dependent transcriptional repression, we characterized endogenous SETDB1-containing complexes from mESCs by immunoprecipitation (IP) and mass spectrometry (MS), utilizing conditions that minimize de-SUMOylation of proteins given the SUMO-dependent interactions between SETDB1 and KAP1 [18]. Indeed, the Sentrin/SUMO-specific proteases SENP1 and SENP7 can de-SUMOylate KAP1 [20, 21] and are expressed in mESCs [22]. To enrich for candidate SUMO-dependent binding partners of SETDB1, we performed an anionic exchange step which efficiently depleted SENP1 followed by IP of endogenous SETDB1 with a specific N-terminal antibody [23] (Fig. 1A and 1B). MS analysis revealed the specific enrichment of KAP1 along with the previously described SETDB1 co-factor MCAF1 (also called mAM/ATF7IP) (Table 1), which directly interacts with SETDB1 independent of SUMOylation [18, 24]. Detection of MCAF1 and KAP1 supports the validity of this approach to identify candidate SUMO-independent and SUMO-dependent binding partners. MS analysis of a SETDB1 IP without prior SENP depletion identified a different set of polypeptides associated with SETDB1 (S1A Fig.). While MCAF1 was identified in this direct IP approach, KAP1 was not (S1A Fig.), indicating that the presence of SENPs in mESC nuclear extracts precludes the association of SETDB1 with its SUMO-dependent binding partners, including KAP1. Among the novel SETDB1-associated proteins detected in the SENP-depleted but not the direct IP, we chose to focus on hnRNP K (Table 1), a ubiquitously expressed protein that functions as a DNA/RNA-binding transcriptional co-activator or co-repressor [25]. Notably, *Hnrnpk* is highly expressed in the inner cell mass and in mESCs relative to earlier stages of development in the preimplantation embryo [22] and was previously reported to directly interact with the KRAB-ZFPs Zik1 and Kid1 [25, 26].

**Table 1:** Mass spectrometric analysis of SETDB1 (medium) versus IgG (light) IPs from the 0.5 M anionic column fraction.

**Protein**	**% Coverage**	**# Peptide Spectral Matches**	**# Unique Peptides**	**Medium/Light Ratio**
SETDB1	13.54	17	14	22.05
MCAF1	10.03	10	8	41.27
KAP1	9.59	7	6	2.92
hnRNP K	17.31	7	5	2.87
BAF155	3.78	3	3	2.74
TRIP12	1.19	2	2	2.37
Nup155	2.01	2	2	2.82
MCM5	3.68	2	2	3.45
ZFP161	4.23	2	2	44.46
PRMT1	7.23	2	2	3.01

We further validated the interaction between hnRNP K and SETDB1 in mESCs using a combination of co-IP, immunostaining and co-sedimentation assays. Both KAP1 and hnRNP K were detected in FLAG-tagged SETDB1 complexes immunopurified from mESCs in the presence of the cysteine protease inhibitor N-ethylmaleimide (NEM), which blocks SENP activity [27] (Fig. 1C). Moreover, using a specific antibody raised against an internal epitope of SETDB1 [28], both KAP1 and hnRNP K co-precipitated with SETDB1 from mESC nuclear extract only in the presence of NEM (Fig. 1D). The association of hnRNP K and SETDB1 was also apparent by immunostaining, which revealed that hnRNP K and SETDB1 colocalize in the nucleus and to a lesser extent the cytoplasm of mESCs upon a short incubation with NEM (S1B Fig.). Reciprocally, SETDB1 co-precipitated with both KAP1 and hnRNP K in the presence of NEM and hnRNP K and KAP1 also co-precipitated with each other (Fig. 1E), indicating that these proteins are present in a single complex. Notably, the IP of KAP1 was clearly more efficient in the presence of NEM (Fig. 1E), revealing that SENP inhibition may stabilize KAP1 oligomeric state, as KAP1 is known to form oligomers [29]. In addition, although hnRNP K binds to both DNA and RNA sequences [30, 31], the interaction between SETDB1 and hnRNP K was not perturbed in the presence of RNAse A and DNase I (S1C Fig.) indicating that it is not dependent upon nucleic acid. Consistent with the finding that the KAP1 IP was more efficient in the presence of NEM (Fig. 1E), sucrose gradient ultracentrifugation of mESC nuclear extracts revealed that SENP inhibition promotes the stability of SETDB1/KAP1/hnRNP K complexes, which migrated at higher density compared with the profile of purified GST-KAP1 and GST-hnRNP K (S1D–S1E Fig.). Although most of the hnRNP K remained uncomplexed with SETDB1/KAP1, a fraction of total nuclear hnRNP K clearly co-sedimented with SETDB1 and KAP1 at a higher density in fractions 9–11 in the presence of NEM (S1E Fig.), compared with GST-hnRNP K in fraction 5 (S1D Fig.). Together these results confirm that hnRNP K is associated with the SETDB1/KAP1 complex in mESCs.

### hnRNP K directly interacts with KAP1

**Figure 2:**
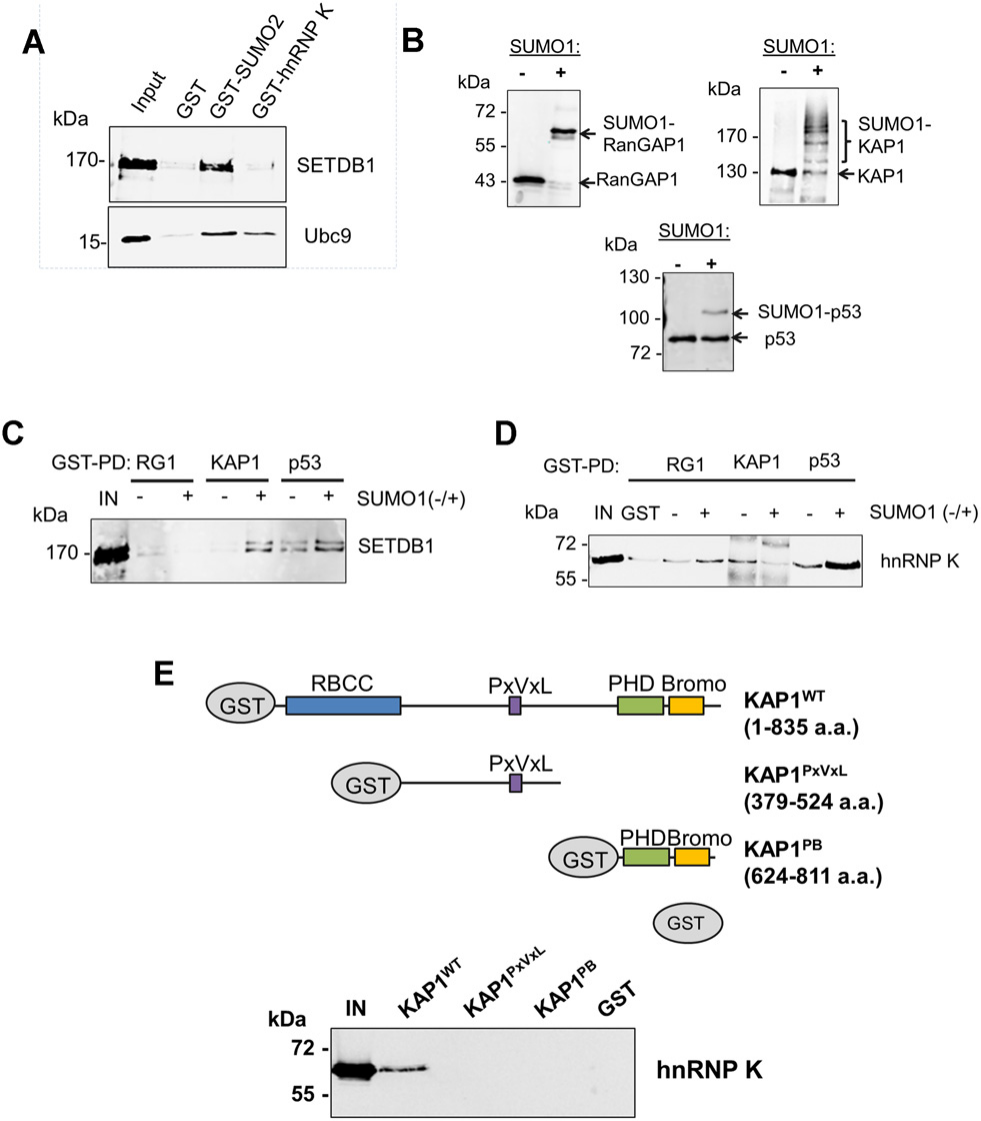
hnRNP K directly interacts with unmodified KAP1 in a region containing the RBCC domain. (A) GST pulldown assays using purified GST, GST-SUMO2 or GST-hnRNP K as baits with recombinant FLAG-SETDB1 or Ubc9 as prey proteins. ‘IN’ represents ~20% of pulldown prey protein input. (B) Western blot analysis of *in vitro* SUMOylation reactions performed on GST-tagged RanGAP1 C-terminal fragment (residues 419–587), full-length wt GST-KAP1 or GST-p53. + or—indicates presence or absence of SUMO1 in the reaction. (C) GST pulldown assays using SUMOylated or unmodified GST-tagged baits from (B) with purified recombinant FLAG-tagged SETDB1 as prey protein. ‘IN’ represents 10% of input SETDB1 prey protein. (D) GST pulldown assay as in (C) except using purified recombinant 6X-His-tagged hnRNP K as prey protein. (E) Schematic of wt KAP1 domain structure and KAP1 mutants used in GST pulldown assays. Unmodified GST-tagged wt KAP1, deletion mutants KAP1^PxVxL^ and KAP1^PB^ or GST alone were used as baits to pull down purified recombinant 6X-His-tagged hnRNP K. ‘IN’ represents 15% of the input hnRNP K protein.

To determine whether hnRNP K directly binds to SETDB1, we performed GST pulldown assays with recombinant SETDB1 or Ubc9 as a positive control protein for hnRNP K [32, 33]. Although KAP1 is SUMOylated in the SETDB1 complex under standard tissue culture conditions [18], hnRNP K was also reported to be SUMOylated but only following DNA damage [32, 33]. Indeed, whereas SETDB1 complexes from mESCs contained both SUMOylated and unmodified KAP1, we found no evidence of SUMOylated hnRNP K (S2A Fig.) and thus used unmodified hnRNP K in subsequent pulldown assays. In contrast with Ubc9, which bound to SUMO2 and hnRNP K, SETDB1 bound to SUMO2 but not hnRNP K (Fig. 2A). In addition, no interaction was detected between FLAG-tagged SETDB1 and T7-tagged hnRNP K upon co-expression and FLAG IP from 293T cells (S2B Fig.). Together these data indicated that hnRNP K does not directly interact with SETDB1.

To determine whether hnRNP K directly binds SUMOylated and/or unmodified KAP1, we prepared *in vitro* SUMO1-conjugated GST-tagged KAP1, GST-p53 as a positive control binding partner of hnRNP K [33], or a GST-tagged fragment of RanGAP1 as a model SUMO1 substrate for GST pulldown assays with recombinant SETDB1 or hnRNP K baits. Using purified SUMOylation cascade components, we achieved efficient mono-SUMOylation of RanGAP1 at K526 [34] and mono-, di-, tri- and tetra-SUMOylation of KAP1 (Fig. 2B) at its major SUMO acceptor lysines including K554, K676, K779 and K804 [18, 19]. p53 was mono-SUMOylated at K386 [35] although this was less efficient in the absence of a SUMO E3 ligase (Fig. 2B). While SETDB1 directly bound to p53 independently of SUMOylation, it bound to KAP1 in a SUMO1-dependent manner (Fig. 2C), consistent with previous observations [18]. HnRNP K binding to p53 was enhanced by SUMOylation but surprisingly, its binding to KAP1 was decreased upon SUMOylation (Fig. 2D).

KAP1 harbours several functional domains that participate in protein-protein interactions, including an N-terminal RING-B-box-coiled-coil (RBCC) domain, which mediates binding to KRAB-ZFPs and other proteins [36–38], a proline-x-valine-x-leucine (PxVxL) motif, which binds to HP1 proteins [39] and a C-terminal PHD finger-bromodomain that binds to Ubc9 and chromatin-modifying factors, including SETDB1 and CHD3 upon SUMOylation [10, 18, 40]. While hnRNP K bound to wt full-length KAP1, it failed to bind to the deletion fragments containing only the PxVxL motif or only the PHD finger-bromodomain (Fig. 2E), revealing that hnRNP K binding requires the N-terminal RBCC domain. Taken together, these observations indicate that hnRNP K and SETDB1 indirectly interact with each other via their binding to unmodified or SUMOylated KAP1 subunits. Consistent with this model, SETDB1 complexes in mESCs contain both unmodified and SUMOylated KAP1 (S2A Fig.), despite SETDB1 exhibiting binding affinity for only SUMOylated KAP1 (Fig. 2C). Furthermore, the interactions between hnRNP K and KAP1 in mESCs were unperturbed upon depletion of SETDB1 (S2C Fig.), confirming that they interact in a SETDB1-independent manner. In addition, endogenous KAP1 also co-precipitated with hnRNP K from 293T cell extracts (S2D Fig.), indicating that this interaction is not limited to mESCs. Finally, the observation that hnRNP K colocalized with KAP1 throughout the nucleus in mESCs in the absence of SENP inhibitor (S2E Fig.) is consistent with the model that they form complexes in the absence of KAP1 SUMOylation in cells.

### Depletion of hnRNP K disrupts SETDB1-dependent proviral silencing

We next investigated whether loss of hnRNP K compromises SETDB1-dependent transcriptional silencing of ERVs in mESCs. Using siRNA-mediated knockdown (KD), hnRNP K was efficiently depleted at the protein level by 24 h post-transfection (S3A Fig.). Notably, KD of hnRNP K in mESCs significantly reduced their proliferation by 72 h post-transfection (S3B Fig.). However, there was no gross effect on cell cycle distribution at this time-point and only minimal effects on expression of the pluripotency marker SSEA1 (S3C–S3D Fig.). Furthermore, reduced proliferation was not associated with induction of apoptosis, as determined by Annexin V staining (S3D Fig.). Thus hnRNP K KD does not result in overt differentiation or apoptosis at this time-point.

**Figure 3:**
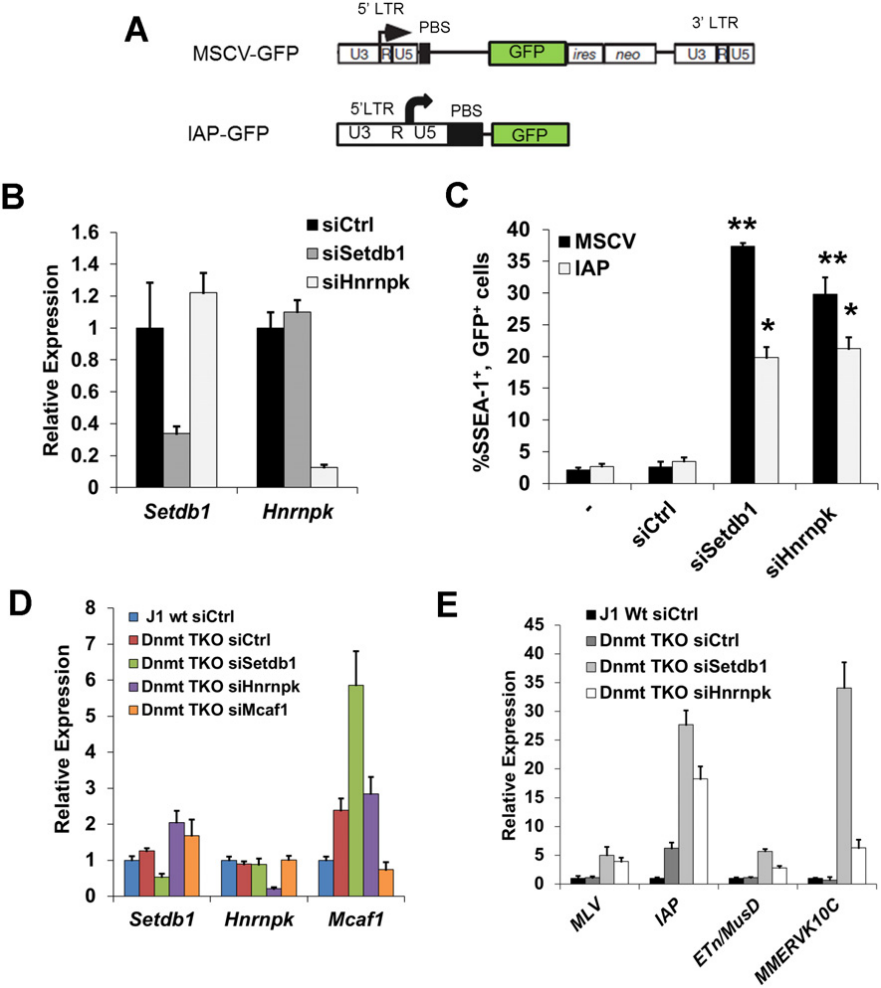
siRNA knockdown of hnRNP K results in de-repression of proviral reporters and ERVs. (A) Schematics of the MSCV (PBS^Gln^)-GFP retroviral vector in the *Setdb1^lox/-^*mESC line and the IAP LTR-PBS GFP reporter in the HA36 wt mESC line. (B) qRT-PCR analysis of *Setdb1* and *Hnrnpk* mRNA in *Setdb1^lox/-^* 33#6 MSCV-GFP cells transfected with control (siCtrl), *Setdb1* or *Hnrnpk* siRNAs at 24 h post-transfection. Data are mean expression level relative to *Gapdh* mRNA and normalized to siCtrl. Data are means (+ s.d.) of three technical replicates. (C) Flow cytometry analysis on untransfected (-) cells and cells transfected with indicated siRNAs at 72 h post-transfection. Data represent mean percentage of SSEA1^+^, GFP^+^ (double-positive) cells from three biological replicates of 10,000 propidium iodide-negative (PI^-^) cells per sample. Error bars are s.d. (D) qRT-PCR analysis of *Setdb1*, *Hnrnpk* and *Mcaf1* mRNA in J1 wt or *Dnmt3a^-/-^; Dmnt3b^-/-^; Dnmt1^-/-^*(*Dnmt* TKO) cells transfected with indicated siRNAs at 24 h post-transfection. Data are mean expression level (+ s.d.) normalized to expression levels in control siRNA transfected J1 wt mESCs, relative to level of *Gapdh* mRNA. (E) qRT-PCR analysis as in (D) of except for intact class I and II ERVs in J1 wt or *Dnmt* TKO mESCs transfected with the indicated siRNAs at 96 h post-transfection. *p < 0.01, **p < 0.001, Student’s two-tailed T-test, relative to siCtrl.

To determine the influence of hnRNP K depletion on proviral silencing, we used previously established proviral GFP reporter mESC lines, including the murine stem cell virus bearing a glutamine tRNA PBS (MSCV-PBS^Gln^) GFP line [11] and the HA36 mESC line, which harbours a silent IAP LTR-PBS-5’UTR region driving GFP transgene integrated into a defined genomic locus [41] (Fig. 3A). In both lines, proviral silencing is dependent upon H3K9me3 deposited by the SETDB1/KAP1 complex [11, 41]. Transfection of siRNAs specific for *Setdb1* or *Hnrnpk* effectively reduced expression of the relevant mRNAs to ~10–25% of the control siRNA-transfected cells (Fig. 3B). While only ~2–3% of SSEA1^+^ cells were also GFP^+^ in the untransfected (MSCV and IAP) and siRNA transfected controls, KD of *Setdb1* de-repressed both reporters, resulting in ~37% and ~20% SSEA1^+^; GFP^+^ cells, respectively (Fig. 3C). Strikingly, KD of *Hnrnpk* also consistently de-repressed both the MSCV and IAP reporters, resulting in an average of ~29% and ~20% SSEA1^+^; GFP^+^ cells, respectively (Fig. 3C). We also interrogated the role of the SETDB1 co-factor MCAF1, which facilitates conversion of H3K9me2 to H3K9me3 by SETDB1 [24]. Interestingly, *Setdb1* KD cells showed a ~4-fold upregulation of *Mcaf1* expression (S4A Fig.), indicating that the level of *Mcaf1* expression is sensitive to the level of SETDB1. The MSCV proviral reporter was also de-repressed in *Mcaf1* KD cells, (S4A–S4B Fig.), revealing that this catalytic co-factor of SETDB1 also plays a role in proviral silencing.

We next determined whether KD of hnRNP K disrupts silencing of ERVs. In contrast to the proviral reporter lines, KD of SETDB1 or hnRNP K in TT2 wt mESCs resulted in only modest de-repression (~2-fold) of class I and II ERVs by 72 h post-transfection (S4C–S4D Fig.), with the exception of robust induction of MMERVK10C elements in SETDB1 KD cells, despite efficient depletion of the protein (S4C Fig.). Surprisingly, class III MERVL elements, which are repressed by KAP1 in a SETDB1-independent manner [42], were strongly induced in hnRNP K KD cells (S4D Fig.).

We have shown previously that DNA methylation also plays a role in transcriptional repression of ERVs, particularly of IAP elements, in mESCs cultured in serum [17]. To preclude the influence of DNA methylation, we knocked down *Setdb1* or *Hnrnpk* in *Dnmt3a; Dnmt3b; Dnmt1* triple KO (*Dnmt* TKO) mESCs [43] (Fig. 3D), which are devoid of DNA methylation but maintain SETDB1 binding and H3K9me3 at ERVs [11] and thus solely rely on the SETDB1/H3K9me3 pathway for silencing of these elements. The absence of DNA methylation alone did not perturb silencing of MLV, MMERVK10C and MusD elements, but yielded a ~6-fold upregulation of IAP elements (Fig. 3E), consistent with the finding that IAP elements are modestly upregulated in *Dnmt* TKO cells [11]. In contrast, KD of *Setdb1* expression resulted in a substantial induction of ERVs in these cells (Fig. 3E). These ERVs were also de-repressed upon *Hnrnpk* KD, with IAP elements showing an increase in expression of ~18-fold, ~3-fold greater than the control KD in the *Dnmt* TKO line (Fig. 3E). Depletion of *Mcaf1* in the *Dnmt* TKO cells (Fig. 3D) also resulted in upregulation of class I and II ERVs beyond what was observed in the *Dnmt* TKO line alone (S4E Fig.). Importantly, although IAP elements are strongly induced in DNA methylation-deficient, differentiated *Dnmt1* ^-/-^; Oct4-negative mESCs [6], levels of *Oct4* mRNA was not appreciably reduced in any of these KD cultures compared to the control siRNA *Dnmt* TKO cells (S4F Fig.), indicating that ERVs were not induced as a secondary consequence of an increase in the number of differentiated cells in culture. Taken together, these results reveal that depletion of hnRNP K disrupts SETDB1/H3K9me3-mediated silencing of ERVs in mESCs.

### hnRNP K is required for repression of a cohort of male germline genes by SETDB1

**Figure 4:**
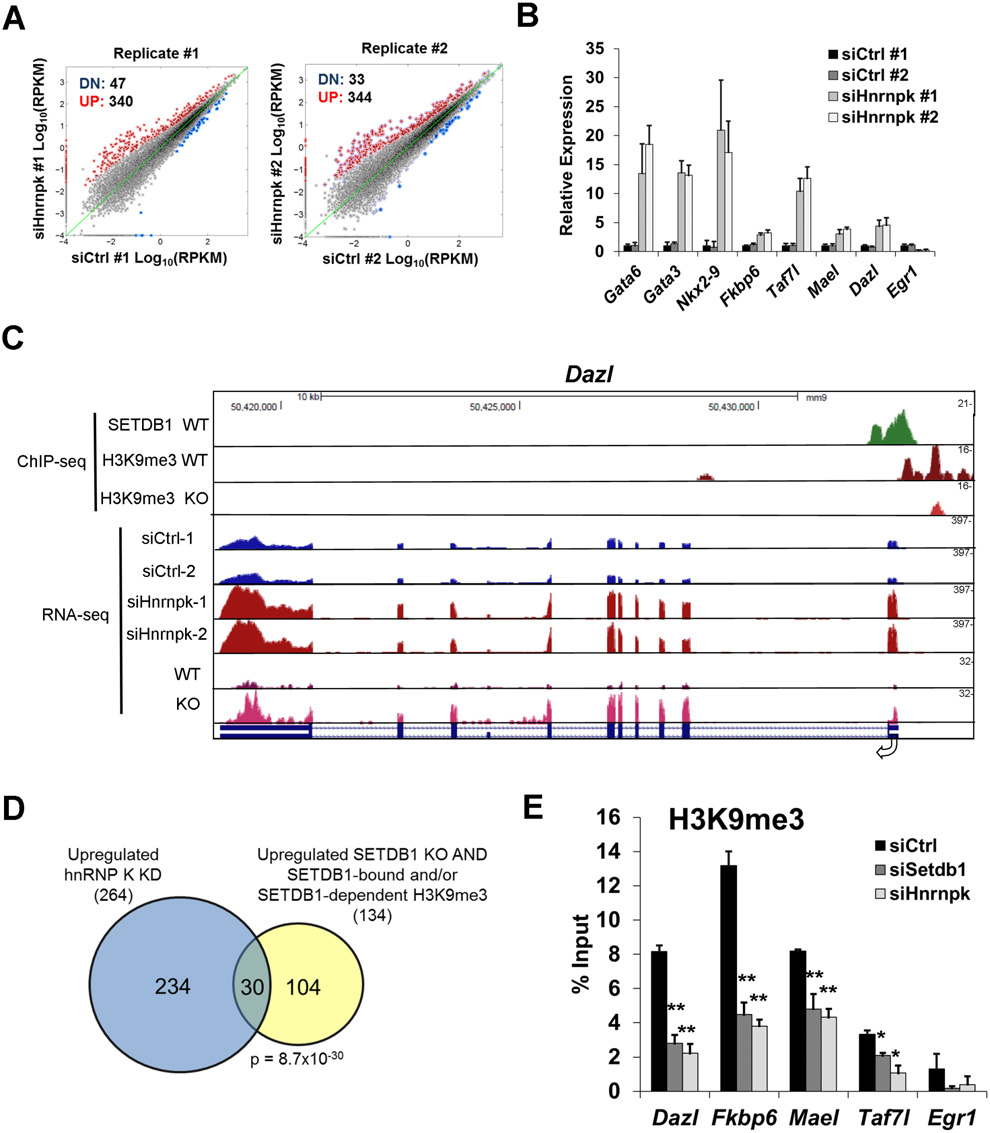
hnRNP K plays a role in repression of SETDB1 target genes. (A) RNA-seq 2D scatterplots of gene expression (reads per kilobase per million mapped reads; log(RPKM) from two biological replicates of TT2 cells transfected with control or *Hnrnpk* siRNAs at 72 h post-transfection. Genes upregulated ≥2-fold (‘UP’) in hnRNP K KD cells are labelled in red, whereas genes downregulated ≥50% (‘DN’) are shown in blue. Data points correspond to n = 22,138 ENSEMBL annotated genes. (B) qRT-PCR analysis of selected lineage-specific genes identified as upregulated from the RNA-seq of hnRNP K KD cells. *Fkbp6*, *Dazl*, *Mael*, *Taf7l* are direct SETDB1/H3K9me3 target genes. Data are relative mean expression level (+ s.d.) for three technical replicates. (C) UCSC genome browser screenshot including tracks for SETDB1 ChIP-seq in wt [28] and H3K9me3 ChIP-seq in wt and *Setdb1* KO mESCs and RNA-seq for wt and *Setdb1* KO mESCs [17] along with total coverage tracks for hnRNP K KD replicates RNA-seq at the *Dazl* gene. Numbers on the right indicate y-axis scale. (D) Venn diagram showing overlap of genes upregulated in hnRNP K KD cells (264) and genes upregulated in SETDB1 KO cells that are either bound by SETDB1 and/or marked by SETDB1-dependent H3K9me3 (134) according to [17]. p = 8.7×10^-30^, Fisher’s exact test (n = 22,138 ENSEMBL-annotated genes). (E) Native ChIP for H3K9me3 at the core promoters/TSS of the indicated genes on TT2 cells transfected with control, *Setdb1* or *Hnrnpk* siRNAs at 72 h post-transfection. Data are mean enrichment relative to input from three technical replicates, error bars are s.d. *p < 0.05, **p < 0.005, Student’s two-tailed T-test, relative to siCtrl.

To investigate whether depletion of hnRNP K disrupts SETDB1-dependent repression of genes, we performed mRNA-seq from two biological replicates of TT2 mESCs transfected with control or *Hnrnpk* siRNA (S4C Fig.). A total of 290 genes were consistently misregulated upon hnRNP K KD, 264 genes were upregulated ≥ 2-fold in both KD lines while only 26 were downregulated by ≥50% (Fig. 4A and S1 Table). Gene ontology (GO) analysis revealed that the upregulated genes were enriched for “apoptosis” (S5A Fig.) indicating that although these KD cells do not show high levels of Annexin V staining at this time-point, their progressive proliferation block (S3B Fig.) may coincide with induction of the apoptotic pathway. Although hnRNP K regulates the expression of pro-apoptotic genes *Bcl-Xs* and *Bik* under certain conditions [44], these genes were not upregulated in hnRNP K KD mESCs. Nevertheless, *Btg2*, *Anxa8*, *Perp*, *Trp73*, *Cdkn1a* and *Casp14* were among the 16 apoptosis-associated genes identified by GO analysis. In addition there was an enrichment of genes involved in “lung and respiratory system development” (S5A Fig.) including the primitive endoderm and mesoderm lineage transcription factors *Gata6*, *Gata3*, *Tbx3*, *Tbx20*, *Foxa1*, *Nkx2–9* and *Nkx2–2* (S1 Table). Previous ChIP-seq data indicates that these transcription factor genes harbour the bivalent chromatin state of H3K4me3 and H3K27me3 [45–47] and are subject to polycomb repressive complex 2 (PRC2)-mediated silencing [48]. The de-repression of *Gata6* and *Gata3*, which were upregulated ~15-fold and ~10-fold in hnRNP K KD cells, respectively (Fig. 4B), indicates that hnRNP K KD could eventually lead to a loss of pluripotency, since the overexpression of these transcription factors is sufficient to drive endoderm lineage differentiation [49, 50]. RNA-seq analysis of ERVs in the TT2 hnRNP K KD cells (S1 Table) generally confirmed our qRT-PCR analysis from the same cells (S4D Fig.) in that class I and II elements were only modestly de-repressed (≤2-fold) while MERVL elements were strongly de-repressed (≥14-fold).

We next compared the list of genes upregulated in hnRNP K KD mESCs to our list of upregulated genes in *Setdb1* KO mESCs [17], which revealed 54 genes in common (S2 Table). We previously identified a cohort of 33 germline lineage genes that are directly repressed by SETDB1-dependent H3K9me3 and DNA methylation [17]. Notably, many of these direct SETDB1 target genes were consistently upregulated >2-fold in hnRNP K KD cells (15 of these genes are shown in S5B Fig.) For example, the promoter of the male germline gene *Dazl* harbours a peak of SETDB1 binding and SETDB1-dependent H3K9me3 and is upregulated in both *Setdb1* KO and hnRNP K KD cells (Fig. 4C). In addition, of the 134 SETDB1-bound genes that are upregulated in *Setdb1* KO mESCs [17], 30 were consistently de-repressed in hnRNP K KD cells (Fig. 4D and S2 Table). Quantitative RT-PCR analysis of a subset of these genes, including the male germline-specific genes *Dazl*, *Fkbp6*, *Mael* and *Taf7l* confirmed that they are indeed upregulated in hnRNP K KD cells (Fig. 4B). Furthermore, levels of H3K9me3 at the promoters of these genes were reduced in hnRNP K KD cells to a similar extent as in SETDB1 KD cells (Fig. 4E). A comparison of the genes upregulated in hnRNP K KD and *Kap1* KO cells [14] also revealed a significant overlap (S5C Fig.) and included lineage-restricted genes such as *Gata6*, *Arg2* and *Dkk1*(S2 Table). We identified 33 genes that are commonly de-repressed in *Setdb1* KO, *Kap1* KO and hnRNP K KD mESCs, several of which were direct SETDB1 targets and were expressed in a lineage-dependent fashion, including the imprinted gene *Igf2* and liver-specific gene *Cml2*(S2 Table). The promoter of *Cml2* lies immediately downstream of an intact ETn family retroelement that is bound by SETDB1 and marked by SETDB1-dependent H3K9me3, which spreads into the *Cml2* promoter (S5D Fig.) indicating that this gene is silenced by the spreading of H3K9me3 from the intact ERV. In conclusion, these results support a role for hnRNP K in transcriptional repression of genes regulated by SETDB1 and KAP1 as well as PRC2, the latter via an undefined pathway.

### H3K9me3 enrichment on proviral chromatin is reduced following knockdown of hnRNP K

**Figure 5:**
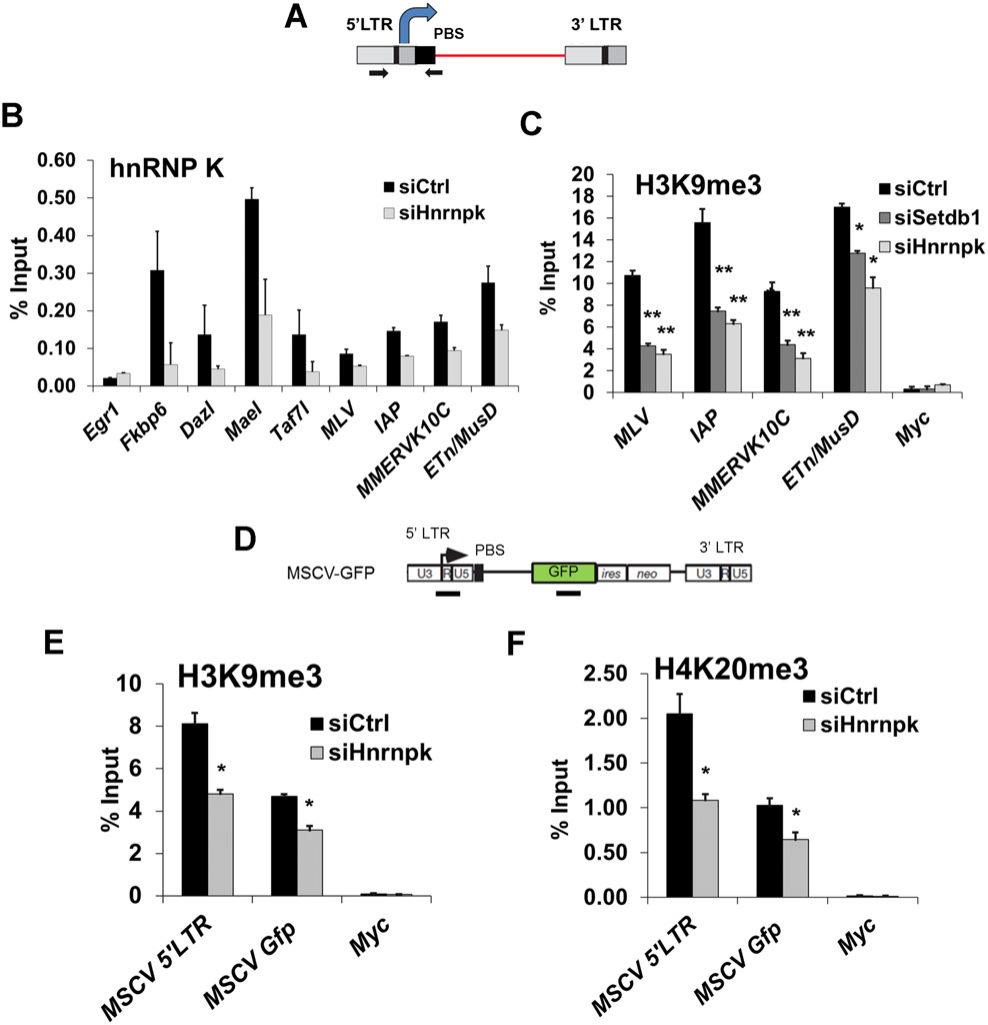
hnRNP K is bound at ERVs and is required for H3K9me3 deposition at proviral chromatin. (A) Schematic of intact ERV structure and 5’LTR-internal sequence amplified (primers shown as arrows). (B) Crosslinked ChIP of hnRNP K in TT2 cells transfected with control or *Hnrnpk* siRNAs at 24 h post-transfection. *Egr1* core promoter and TSS (-50 to +50) was amplified as a negative control locus. Data shown are mean enrichment levels relative to input material from three technical replicates, error bars show s.d. (C) Native ChIP for H3K9me3 at the indicated ERV 5’LTR-internal regions in TT2 wt mESCs transfected with control, *Setdb1*, or *Hnrnpk* siRNAs at 72 h post-transfection with the same primers as in (A). The *Myc* core promoter and TSS (-50 to +50) was amplified as a negative control. (D) Schematic of the MSCV (PBS^Gln^)-GFP vector with black bars showing the positions of ChIP amplicons for the MSCV 5’LTR-PBS region and *Gfp* internal sequence. (E) Native ChIP for H3K9me3 as in (C) except on siRNA KD of *Hnrnpk*in unsorted (GFP^+^ and GFP^-^) MSCV-GFP cells at 24 h post-transfection. (F) Native ChIP from the same KD cells as in (E) except for H4K20me3. *p < 0.001, **p < 0.0001, Student’s two-tailed T-test, relative to siCtrl.

We next determined whether hnRNP K is bound at de-repressed ERVs. Since the presence of NEM increased the sensitivity of KAP1 and SUMO1 chromatin immunoprecipitation (ChIP), improving the enrichment of both at MLV and IAP 5’ LTRs where KAP1 binding is high, but not at MERVL 5’ LTRs where KAP1 binding is low [42] (S6A Fig.), we performed subsequent ChIP assays in the presence of NEM to preclude a refractory effect of SENP activity on the binding of these factors at ERV 5’LTRs and other loci (Fig. 5A). Under these conditions, hnRNP K was enriched at the promoters of the SETDB1-bound, H3K9me3-marked germline genes *Fkbp6*, *Dazl*, *Mael* and *Taf7l*, with the highest level of enrichment detected at *Mael* (Fig. 5B), indicating that these loci are direct targets of hnRNP K in mESCs. Relative to the germline gene promoters, the 5’LTRs of class I and II ERVs showed lower enrichment of hnRNP K, with ETn/MusD and MLV elements showing the highest and lowest levels, respectively (Fig. 5B). Importantly, the signal at ERVs and the germline gene promoters was specific, since it was reduced upon hnRNP K KD. In contrast, there was no enrichment of hnRNP K at the *Egr1* promoter (Fig. 5B), which is active in mESCs and was shown to be bound by hnRNP K only upon serum stimulation in the HCT116 colon cancer cell line [51, 52]. Furthermore, RNAse did not perturb hnRNP K enrichment at ERVs (S6B Fig.), indicating that hnRNP K is recruited to class I and II ERVs in an RNA-independent manner.

We next determined whether hnRNP K is required for SETDB1-dependent H3K9me3 deposition at ERVs. As shown previously [11], SETDB1 KD resulted in depletion of H3K9me3 at MLV, IAP, MMERVK10C and ETn/MusD 5’LTRs (Fig. 5C). Strikingly, this effect was phenocopied upon KD of hnRNP K (Fig. 5C). Importantly, the reduction of H3K9me3 at ERVs was apparent in hnRNP K KD cells as early as 48 h post-transfection, similar to the kinetics of H3K9me3 perturbation in SETDB1 KD cells (S6C Fig.), indicating that this phenotype is not a secondary consequence of the loss of proliferation that commences at ~72 h post-transfection in hnRNP K KD cells (S3B Fig.). Furthermore, as early as 24 h after hnRNP K KD, there was a clear reduction of H3K9me3 at the MSCV 5’LTR-PBS and *Gfp* regions (Fig. 5D and 5E). The levels of H4K20me3, a mark deposited by SUV420H1/2 enzymes in a SETDB1/H3K9me3-dependent manner [11], were also reduced at the MSCV provirus in hnRNP K-depleted cells (Fig. 5F). H3K9me3 was dramatically reduced at both ERVs and the MSCV proviral reporter in MCAF1 KD cells (S6C–S6D Fig.), consistent with its role as a catalytic co-factor of SETDB1 [24]. Importantly, siRNA-mediated depletion of hnRNP K or SETDB1 did not affect global H3K9me2 or H3K9me3 levels (S6E Fig.), indicating that the effect of hnRNP K depletion on H3K9me3 at ERVs is not the result of a general reduction of H3K9me2/3. Thus we concluded that hnRNP K is required for SETDB1-dependent H3K9me3 deposition at proviral chromatin.

### hnRNP K is required for SETDB1 recruitment to ERVs

**Figure 6:**
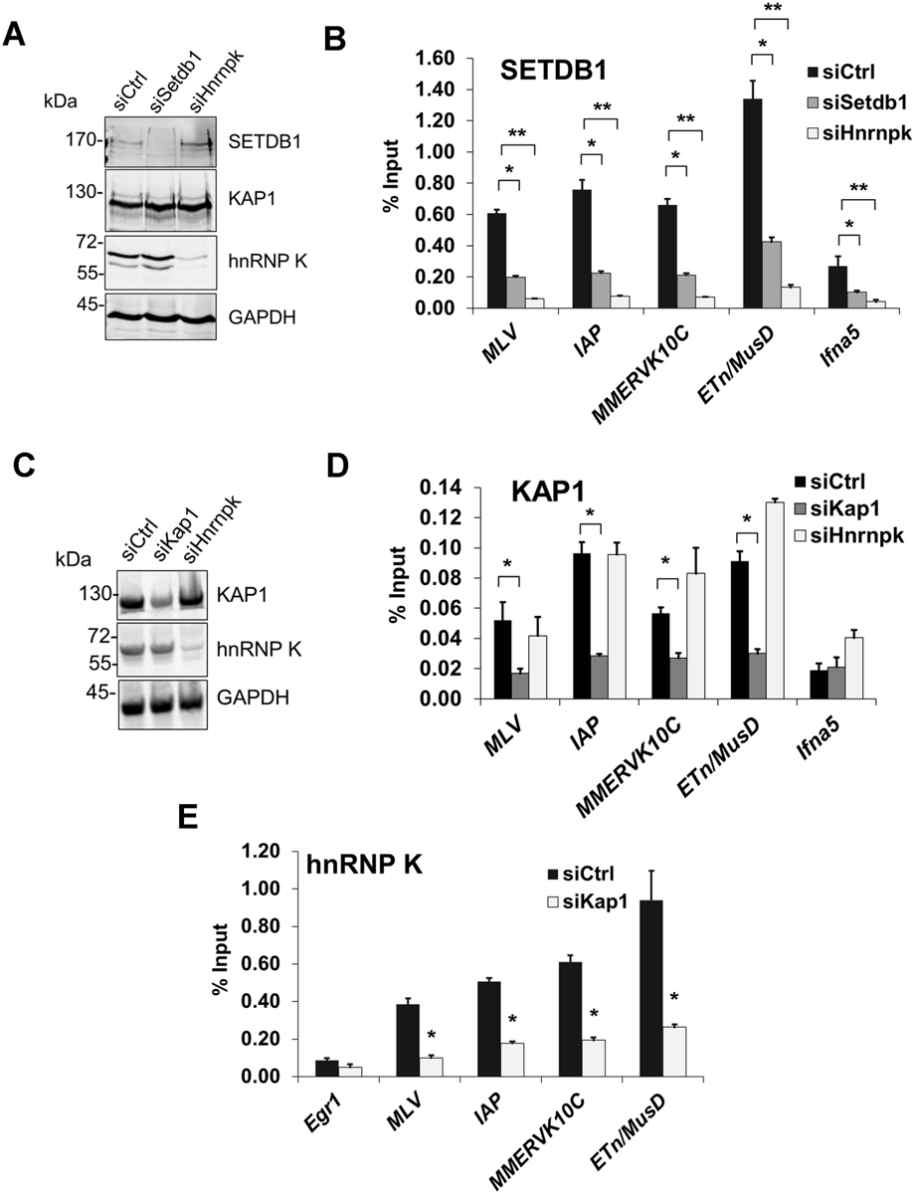
hnRNP K is required for SETDB1 but not KAP1 recruitment to ERVs. (A) Western blot analysis of SETDB1, KAP1 and hnRNP K in TT2 cells transfected with control, *Setdb1* or *Hnrnpk* siRNAs at 24 h post-transfection. GAPDH was detected as a loading control. (B) Crosslinked ChIP of SETDB1 in the same cells as in (B) except at 72 h post-transfection. All qPCR data are mean enrichment relative to input of three technical replicates, error bars are s.d. The *Ifna5* core promoter and TSS (-50 to +50) was amplified as a negative control locus. (C) Western blot analysis of KAP1 and hnRNP K in TT2 cells transfected with control, *Kap1* or *Hnrnpk* siRNAs at 24 h post-transfection. (D) Crosslinked ChIP of KAP1 in the same cells as in (C) except at 72 h post-transfection. (E) Crosslinked ChIP as in (B) and (D) except of hnRNP K in TT2 cells transfected with control or *Kap1* siRNAs at 72 h post-transfection. *p < 0.001, **p < 0.0001, Student’s two-tailed T-test relative to siCtrl.

To determine whether hnRNP K is required for SETDB1 recruitment, we next conducted ChIP analysis of SETDB1 in cells depleted of hnRNP K (Fig. 6A and 6B. A reduction of SETDB1 enrichment was apparent at all class I and II ERV LTRs in SETDB1 KD cells, confirming the specificity of our antibody (Fig. 6B). Strikingly, KD of hnRNP K also reduced the level of SETDB1 enrichment at ERVs (Fig. 6B), likely explaining the reduction of H3K9me3 observed at these loci following hnRNP K KD (Fig. 5C). SETDB1 enrichment was also reduced at the MSCV 5’LTR-PBS and *Gfp* internal region upon depletion of hnRNP K (S7A Fig.), revealing a link between loss of H3K9me3, de-repression of the MSCV proviral reporter and reduced SETDB1 recruitment. In contrast, KD of MCAF1 did not perturb SETDB1 enrichment at ERVs or the MSCV provirus (S7B Fig.). Importantly, neither SETDB1 nor KAP1 protein levels were reduced in hnRNP K KD cells (Fig. 6A) and SETDB1 was still localized to the nucleus in hnRNP K-depleted cells (S7C Fig.).

KAP1 is the only factor known to be required for SETDB1 recruitment to proviral chromatin [11, 14]. While KAP1-depleted cells showed reduced levels of KAP1 enrichment at ERVs confirming antibody specificity, KD of hnRNP K did not affect KAP1 enrichment levels (Fig. 6C and 6D). In contrast, KD of KAP1 substantially reduced hnRNP K enrichment at ERVs (Fig. 6E). Taken together, these data reveal that hnRNP K is recruited in a KAP1-dependent manner and facilitates subsequent SETDB1 binding at proviral chromatin.

### SUMOylation on proviral chromatin is required for SETDB1 recruitment and is reduced upon hnRNP K knockdown

**Figure 7:**
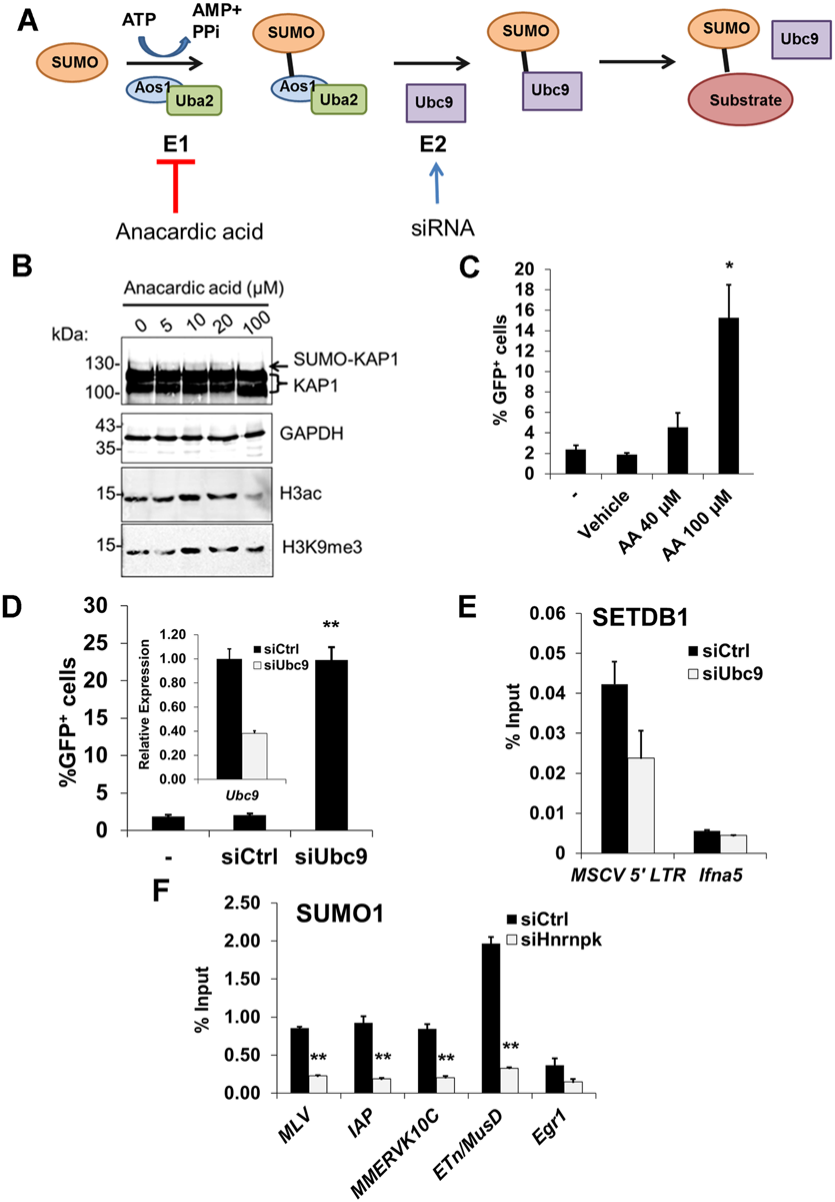
SUMOylation on proviral chromatin is required for SETDB1 recruitment and is compromised upon hnRNP K knockdown. (A) Illustration of the SUMO conjugation pathway including the activities of SUMO E1 activating heterodimer enzyme Aos1/Uba2 and SUMO E2 conjugating enzyme Ubc9. Anacardic acid was used to inhibit E1 activity while siRNAs were used to deplete *Ubc9* transcripts to disrupt SUMO conjugation in the MSCV-GFP cell line. (B) Western blot analysis of KAP1, GAPDH, pan Histone H3 acetylation or H3K9me3 in MSCV-GFP cells incubated in varying concentrations of anacardic acid for 18 h prior to harvest. A mono-SUMOylated KAP1 band is detected at ~130 kDa (arrow). (C) Flow cytometry for GFP^+^ cells in the MSCV-GFP line alone (-), vehicle (DMSO) treated, or treated with 40 or 100 μM anacardic acid for 18 h. Data are mean of three biological replicates for 10,000 PI- cells per sample, error bars are s.d. (D) Flow cytometry data as in (B) except on cells untransfected (-) or transfected with control or *Ubc9* siRNAs at 48 h post-transfection. Inset, qRT-PCR analysis of *Ubc9* expression in cells transfected with control or *Ubc9* siRNAs at 24 h post-transfection. Data are mean relative expression levels determined from three technical replicates, normalized to the level of β-actin (*Actb*) transcripts, error bars are s.d. (E) Crosslinked ChIP of SETDB1 on unsorted (GFP^+^ and GFP^-^) MSCV-GFP cells transfected with control or *Ubc9* siRNAs at 48 h post-transfection. Data are mean enrichment as a percent of input chromatin from three technical replicates, error bars are s.d. (F) Crosslinked ChIP as in (E) except for SUMO1 on TT2 cells transfected with control or *Hnrnpk* siRNAs at 24 h post-transfection. *p < 0.01, **p < 0.001, Student’s two-tailed T-test relative to vehicle or siCtrl.

Previous studies have shown that KAP1 SUMOylation is necessary for recruitment of SETDB1 and H3K9 methylation to promote silencing of heterologous promoters in transformed cell lines [18, 19, 53]. To determine whether a functional SUMOylation pathway is also necessary for SETDB1 recruitment to ERVs in pluripotent stem cells, we used either anacardic acid to inhibit SUMO E1 activating enzyme [54] or siRNAs to KD *Ubc9* (also called *Ube2i*) in the MSCV-GFP cell line and assayed for de-repression of the proviral LTR by flow cytometry (Fig. 7A). In accord with the inhibitory effect of anacardic acid on the activity of SUMO E1 activating enzyme Aos1/Uba2 and histone H3 acetyltransferases such as p300 [55], this compound blocked both KAP1 SUMOylation and bulk histone H3 acetylation (Fig. 7B). While, anacardic acid treatment did not affect bulk H3K9me3 (Fig. 7B), it consistently de-repressed the proviral reporter in a dose-dependent manner, resulting in ~15% GFP^+^ cells at 100 μM (Fig. 7C).

Using siRNAs, we depleted *Ubc9* mRNA to ~35% of the control (Fig. 7D, inset graph). As *Ubc9* is essential for early embryogenesis [56], we monitored changes in MSCV expression at 48 h post siRNA transfection. KD of *Ubc9* expression consistently de-repressed the proviral reporter resulting in an average of 23% GFP^+^ cells (Fig. 7D). Notably, ChIP analysis revealed that SUMO1 levels at the MSCV 5’ LTR were dramatically reduced in Ubc9-depleted cells (S8A Fig.) indicating that the loss of SUMOylation on proviral chromatin correlates with de-repression. Furthermore, there was a reduction of SETDB1 enrichment at the MSCV provirus in *Ubc9* KD cells (Fig. 7E), confirming that SUMOylation of chromatin proteins associated with ERVs enhances SETDB1 recruitment. Strikingly, SUMO1 levels were greatly reduced at the 5’ LTRs of MLV, IAP, MMERVK10C and ETn/MusD elements by 24 h post-transfection of hnRNP K siRNAs (Fig. 7F). Moreover, this effect persisted in hnRNP K KD cells at 72 h post-transfection both at ERVs and the MSCV provirus (S8B–S8C Fig.), coinciding with the timeframe in which SETDB1 recruitment to proviral chromatin was compromised (Fig. 6B). Although the loss of SUMOylation at ERV chromatin upon hnRNP K KD could be a consequence rather than a cause of reduced SETDB1 recruitment, KD of SETDB1, which was sufficient to de-repress the MSCV LTR (S8D Fig.), did not concomitantly attenuate SUMOylation on proviral chromatin (S8E Fig.). Taken together these results are consistent with the model that hnRNP K is necessary for SUMOylation of proteins such as KAP1 on ERV chromatin, which is required for SETDB1 recruitment and in turn proviral silencing.

## Discussion

KRAB-ZFP/KAP1 complexes [9, 57] are thought to play a central role in repression of ERV transcription in pluripotent stem cells via SETDB1 recruitment [11, 14]. In this work, we have identified hnRNP K as a novel co-factor, which is required for recruitment of SETDB1 to proviral chromatin and in turn for efficient proviral silencing. HnRNP K is a highly conserved, multi-functional protein involved in transcription regulation, mRNA splicing and translation [25]. Studies in flies, yeast and in mammalian cell lines reveal that hnRNP K plays important roles in development and gene regulation [58, 59]. HnRNP K was reported to directly interact with chromatin regulatory proteins, such as the PRC2 subunit EED [60] and KRAB-ZFPs Zik1 and Kid1 [25, 26], indicating that it may regulate Polycomb and/or KRAB-ZFP/KAP1 complexes.

**Figure 8:**
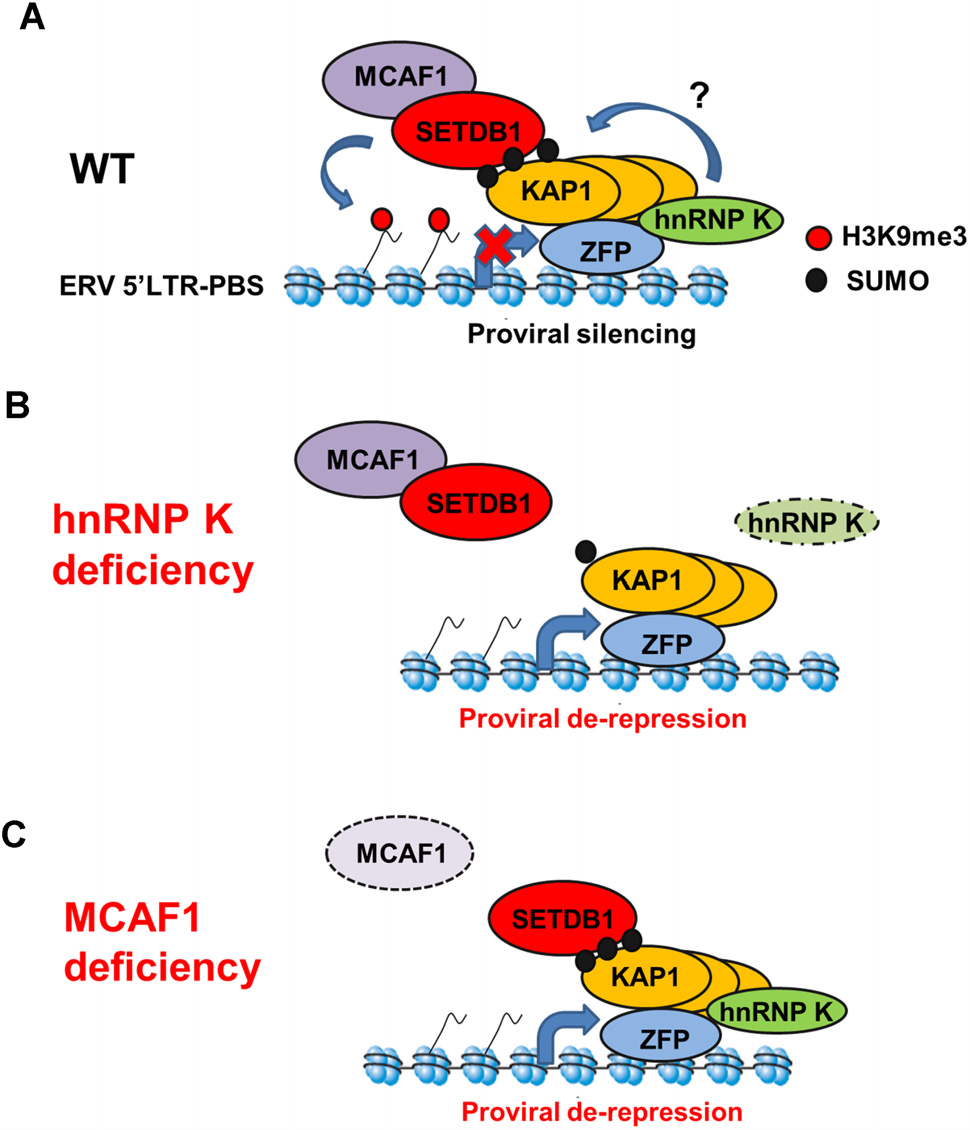
Model for SETDB1/KAP1-mediated proviral silencing pathway. (A) In wt mESCs, KRAB-ZFPs recruit KAP1 subunits and unmodified KAP1 recruits hnRNP K. SUMOylation of KAP1 and/or other proteins on chromatin is promoted by hnRNP K, resulting in recruitment of SETDB1/MCAF1 and deposition of H3K9me3. (B) In hnRNP K-deficient cells, the SUMOylation of KAP1 on chromatin is compromised, leading to loss of SETDB1 recruitment, loss of H3K9me3 and induction of proviral expression. (C) In MCAF1-deficient cells, SETDB1 recruitment is maintained but H3K9me3 is no longer deposited efficiently, leading to proviral de-repression.

Our results reveal a role for hnRNP K in the KRAB-ZFP/KAP1-based silencing pathway acting on ERVs and retroviral vectors in pluripotent stem cells. Based on these findings, we propose a novel model for the SETDB1/KAP1 proviral silencing pathway incorporating hnRNP K (Fig. 8). In wt mESCs, KRAB-ZFPs recruit KAP1 in an oligomeric state, possibly as a homotrimer [29, 36], to proviral chromatin and unmodified KAP1 may recruit hnRNP K. HnRNP K may promote KAP1 SUMOylation on chromatin, which then serves as a ligand for the SETDB1/MCAF1 complex [18, 61], eliciting SETDB1-dependent H3K9me3 deposition at SUMOylated KAP1-bound regions (Fig. 8A). In hnRNP K-deficient cells, SUMOylation of KAP1 on chromatin may be compromised, leading to reduced SETDB1 recruitment at ERVs, diminution of H3K9me3 and eventual transcriptional de-repression (Fig. 8B). This model is consistent with recent ChIP-seq analyses of SUMO1, SUMO2 and Ubc9 in human fibroblasts [62], which show co-occupancy with sites of KAP1, SETDB1 and H3K9me3 at the 3’ ends of KRAB-ZFP genes [63] indicating that these SETDB1/KAP1-bound, SUMOylated loci are sites of active KAP1 SUMOylation on chromatin [62]. Although a possible contraindication to this model is our observation of the differing binding affinities of SETDB1 and hnRNP K for SUMOylated KAP1 *in vitro* (Fig. 2C and 2D), this could be rationalized by: 1) the existence of multiple KAP1 subunits in each complex such that some are SUMOylated while others are unmodified, providing binding sites for both SETDB1 and hnRNP K simultaneously, and/or 2) the observation that hnRNP K directly binds to certain KRAB-ZFPs [25, 26] and therefore may still indirectly interact with SUMOylated KAP1. Indeed, consistent with the former possibility, rather than solely containing SUMOylated KAP1, we found that SETDB1 complexes contained predominantly unmodified KAP1 with only a minority SUMOylated KAP1 under conditions where we could preserve mono- and di-SUMOylated KAP1 in mESC nuclear extracts (S2A Fig.). This observation indicates that SETDB1 binding to KRAB-ZFP/KAP1 complexes *in vivo* may only require a small proportion of the total KAP1 in the complex to be SUMOylated. KAP1 SUMOylation is highly dynamic and previous investigations have relied on overexpression of SUMO paralogues to detect it [19, 20, 38, 64]. Therefore, it is also possible that hnRNP K facilitates transient KAP1 SUMOylation events in a cell cycle-dependent manner, such as during S-phase when chromatin modifications must be re-established. How hnRNP K promotes the SUMOylation of KAP1 remains to be determined, although given that hnRNP K is a SUMO target itself and can directly interact with Ubc9 [32, 33], it may facilitate recruitment of this SUMO E2 enzyme to KAP1-bound loci. Alternatively, hnRNP K might also counteract SENP activity toward KAP1 providing an additional layer of regulation over KAP1de-SUMOylation. Since KAP1 is constitutively phosphorylated at Ser824 in pluripotent stem cells [65], another intriguing possibility is that hnRNP K counteracts the activity of the SUMO-targeted ubiquitin ligase RNF4, which conjugates ubiquitin to Lys676 SUMOylated, Ser824 phosphorylated KAP1 promoting its degradation [64].

Similar to SETDB1 KD mESCs [17], KD of hnRNP K only resulted in modest upregulation of class I and II ERVs in wt mESCs cultured in serum. A likely explanation for this observation is the relatively high level of DNA methylation in mESCs cultured in serum relative to two-inhibitor (2i) media. Under the latter conditions, mESCs adopt a “naïve” hypomethylated state, more reflective of the inner cell mass of the E3.5 blastocyst [46]. Consistent with this model, depletion of hnRNP K in DNA methylation-deficient cells led to a more robust upregulation of class I and II ERVs as compared with wt cells and previous work has shown that IAP elements are synergistically upregulated upon KD of both SETDB1 and DNMT1 in serum-cultured mESCs [17]. Thus siRNA KDs in serum-cultured mESCs are likely not robust enough to elicit loss of DNA methylation at ERVs controlled by SETDB1, despite losses of H3K9me3. In contrast with ERVs, for reasons that are not entirely clear, knocking down SETDB1 in serum-cultured mESCs harbouring a newly integrated silent MSCV provirus results in losses of both H3K9me3 and DNA methylation at the 5’LTR and subsequent de-repression [11, 41].

In addition to hnRNP K, we also identified a crucial role for MCAF1 in SETDB1-mediated proviral silencing, consistent with its role in enhancing SETDB1 catalytic activity towards H3K9me2 to generate H3K9me3 [24]. In contrast to hnRNP K- and KAP1-depleted cells, SETDB1 recruitment is maintained but H3K9me3 is no longer efficiently deposited at proviral chromatin in MCAF1-deficient mESCs (Fig. 8C). This phenotype is consistent with the observation that the catalytic activity of SETDB1 is crucial for full ERV repression [11, 41] and a previous report showing that the MCAF1 orthologue Windei is necessary for dSETDB1/Eggless function in the *Drosophila* germline [66].

Intriguingly, class III MERVL elements, which are silenced by H3K9me2 deposited by the lysine methyltransferases G9a/GLP [42], were strongly induced in hnRNP K KD cells. Since these elements are also de-repressed in *Kap1* KO but not *Setdb1* KO mESCs [14, 42], hnRNP K may play a role in SETDB1-independent chromatin regulatory pathways with KAP1 and G9a/GLP. Further experiments are required to address whether hnRNP K has direct role in MERVL silencing in mESCs.

In addition to ERVs, a cohort of SETDB1/H3K9me3-repressed male germline-specific genes [17] are bound at their promoters by hnRNP K, show reduced H3K9me3 and increased expression upon hnRNP K KD, indicating a role for hnRNP K in SETDB1/H3K9me3-mediated gene repression. How SETDB1 may be targeted to these promoters by hnRNP K remains unclear, since these genes are not upregulated in *Kap1* KO cells [14]. One possibility is that hnRNP K promotes SUMOylation of proteins other than KAP1 on chromatin, leading to SETDB1 binding and transcriptional silencing. In addition to SETDB1/H3K9me3, hnRNP K may also promote PRC2/H3K27me3-mediated gene repression in mESCs, since a cohort of PRC2 target genes, including *Gata6* and *Nkx2–9*were strongly upregulated in hnRNP K KD cells. This is consistent with a previous report showing that hnRNP K can promote PRC2-dependent repression via recruitment of the subunit EED to a heterologous promoter [60]. Further studies will be necessary to clarify the contribution of hnRNP K to SETDB1- and PRC2-mediated transcriptional silencing at specific genes in mESCs.

In conclusion, our results reveal novel mechanistic insights into the transcriptional silencing of class I and II LTR retrotransposons and genes by SETDB1/H3K9me3 in pluripotent stem cells. Notably, both hnRNP K and SETDB1 have been identified as bona fide oncogenes and are aberrantly overexpressed in a variety of human cancers including melanoma [67, 68], prostate carcinoma [69, 70] and lung carcinoma [71, 72]. A greater understanding of how hnRNP K regulates the recruitment of SETDB1 to promoters may ultimately provide new targets for anti-oncogenic therapeutics.

## Materials and Methods

### Cell culture, RNAi and plasmid transfection

Mouse ES cell lines used in this study included: TT2 wt, TT2 33#6 *Setdb1^lox/-^*harbouring the randomly integrated silent MSCV (PBS^Gln^)-GFP [11], TT2 33#6 *Setdb1^lox/-^*expressing 3XFLAG-*Setdb1* [11], HA36 harboring the silent IAP LTR-GFP construct [41], J1 wt and *Dnmt3a^-/-^; Dnmt3b^-/-^; Dnmt1^-/-^*[43]. All ES cell lines were cultured under standard feeder-free conditions on gelatinized tissue culture dishes in standard mESC media: DMEM high glucose containing 15% fetal bovine serum, 20 mM HEPES, 1 mM L-glutamine, 100 U/ml penicillin-streptomycin, 1 mM nonessential amino acids, 1 mM sodium pyruvate, recombinant LIF and 0.1 mM β-mercaptoethanol. HEK293T cells were cultured in DMEM high glucose containing 10% fetal bovine serum and 100 U/ml penicillin-streptomycin. All cell lines were cultured at 37°C with 5% CO_2_. RNAi was performed essentially as described [41] using predesigned siRNA SMARTpools from Dharmacon (ThermoFisher). Briefly, cells were seeded in ES media lacking antibiotics to achieve 70–80% confluence and 24 h later were transfected with 100 nM of SMARTpools for *Setdb1*, *Hnrnpk*, *Mcaf1* (also called *Atf7ip*), *Kap1*, *Ubc9* or non-targeting siRNA #2 (control siRNA) using DharmaFECT Reagent #1. A second round of transfection was performed 48 h later using 100 or 50 nM siRNAs. Plasmids were transfected into HEK293T cells in antibiotic-free media using lipofectamine 2000 (Life Technologies) and harvested 48 h post-transfection. For blocking SUMO E1 activity, anacardic acid (Sigma-Aldrich) diluted to 5–100 μM in DMSO was incubated with cells in complete ES media for 18 h prior to harvest.

### Immunofluorescence and flow cytometry

Indirect immunofluorescence staining was performed using standard methods. Cells were grown on coverslips or harvested by trypsinization were crosslinked with 4% formaldehyde, permeabilized with 0.25% triton-X-100 and blocked with 1% bovine serum albumin (Sigma-Aldrich). Cells were then incubated with anti-SETDB1 H300 (Santa Cruz Biotechnology sc-66884), anti-hnRNP K 3C2 (Abcam 39975), anti-hnRNP K (Abcam 70492) or anti-KAP1 20C1 (Abcam 22553) at 37°C for 1 h or overnight at 4°C and subsequently incubated with Alexa Fluor 488 and 594-labeled secondary antibodies (Life Technologies). DNA was counterstained with Hoescht 33342 (Sigma-Aldrich). Flow cytometry analysis of GFP-fluorescing cells was performed as previously described [41]. Briefly, cells were resuspended in 0.5 μg/ml propidium iodide (Sigma-Aldrich) in FACS buffer (phosphate buffered saline containing 3% fetal bovine serum) and analyzed on a BD LSRII flow cytometer using BD FACS Diva software. Cells were successively gated on forward and side scatter, then PI- (live cells) and lastly GFP^+^ cells, using the untransfected mESC line (either MSCV-GFP or IAP-GFP) as a GFP- population to set the gates. SSEA1 and Annexin V staining were detected on mESCs using 1:400 anti-SSEA1 PE-conjugate (BD Pharmigen) or 1:1000 anti-Annexin V Alexa Fluor 488-conjugate (Life Technologies). Where indicated, cells were gated for the SSEA1^+^ population prior to GFP gating to identify the SSEA1^+^; GFP^+^ (double-positive) population. Cell cycle analysis was performed according to standard methods where cells were harvested and fixed for >2 h in ice-cold 70% ethanol, permeabilized with 0.25% triton-X-100 and stained with 10 μg/ml propidium iodide (Sigma-Aldrich). Cell cycle profiles were analyzed by the Dean-Jett-Fox Model using FlowJo software (Tree Star).

### Mass spectrometry of SETDB1 complexes

Nuclear extracts were prepared from mESCs as previously described [42] with or without 10 or 20 mM NEM and clarified by centrifugation. For immunoprecipitation of SETDB1 complexes after anionic column fractionation, approximately 12–15 mg of TT2 mESC nuclear extract (4 ml) was prepared without NEM, diluted with 2 volumes with 56 mM HEPES pH 7.9, 5% glycerol and passed over a 2 ml column of Macro HiQ anionic exchange media (BioRad) in an equilibration buffer (50 mM HEPES pH 7.9, 100 mM KCl, 10% glycerol). Bound proteins were washed with 5 column volumes of equilibration buffer and then eluted stepwise in 2 volumes of buffer containing 250 mM KCl, then 2 volumes of buffer containing 500 mM KCl. The 500 mM KCl fraction containing SETDB1 and depleted of SENP1 (4 ml) was then diluted with 2 volumes IP dilution buffer (20 mM HEPES pH 7.9, 0.5% NP-40, 10% glycerol containing 2 mM PMSF) and divided into two equal aliquots and immunoprecipitated overnight at 4°C with protein G sepharose beads crosslinked with ~100 μg of rabbit IgG (Sigma Aldrich) or rabbit anti-SETDB1 H300 (Santa Cruz Biotechnology) using dimethylpimelimidate. Beads were washed extensively with a wash buffer (20 mM HEPES pH 7.9, 200 mM KCl, 1% NP-40, 0.1% sodium deoxycholate, 10% glycerol) and eluted by boiling in SDS-PAGE loading buffer. For direct SETDB1 IP from mESC nuclear extract, ~7–8 mg of nuclear extract (1.5 ml) was diluted with 2 volumes of IP dilution buffer as above and incubated with 30 μg rabbit IgG or anti-SETDB1 H300 overnight at 4°C. Immunocomplexes were captured on protein G dynabeads, washed extensively with wash buffer as described above except omitting deoxycholate, eluted with 0.1 M glycine pH 2.5 and neutralized with 1.5 M Tris pH 8.8. Immunoprecipitated samples were analyzed by SDS-PAGE, western blot and silver staining.

For mass spectrometry, IgG and SETDB1 IP samples were resolved by SDS-PAGE and stained with colloidal coomassie. The IgG heavy and light chain bands were removed first and discarded then the rest of each gel lane was excised and subjected to in-gel digestion [73]. Extracted peptides were then analyzed by nano-flow liquid chromatography-tandem mass spectrometry (LC-MS/MS) on a LTQ-Orbitrap Velos Pro mass spectrometer (ThermoFisher) [74]. Tandem mass spectra were searched against the UniProt mouse database using Mascot (v2.4, Matrix Science). Each IP sample was analyzed independently twice. The final refined hit list of proteins was filtered for nuclear proteins with enrichment ratios of SETDB1 IP/IgG IP (medium/light) of >2, >2 unique peptides and >2 independent spectra.

### Protein extraction, immunoprecipitation and western blotting

Native whole-cell extracts for immunoprecipitation were prepared from mESCs and 293T cells by lysing cells in 20 mM HEPES, 200 mM KCl, 1% NP-40, 1 mM EDTA, 10% glycerol, containing 1 mM DTT, 10 or 20 mM NEM, complete EDTA-free protease inhibitor cocktail (Roche) and PhosStop phosphatase inhibitor cocktail (Roche). For preparing cell extracts for western blotting, cells were lysed in RIPA buffer (50 mM Tris pH 8.0, 150 mM NaCl, 1% NP-40, 0.25% deoxycholate, 0.1% SDS). Histones were isolated from mESCs for westerns by acid-extraction of nuclei with 0.2 M HCl or by boiling cells in SDS-PAGE loading buffer. For IP, nuclear extract (100 μl) was diluted with 2 volumes of IP dilution buffer (20 mM HEPES pH 7.9, 1.5 mM MgCl_2_, 0.5% NP-40, 10% glycerol). Protein samples were immunoprecipitated overnight at 4°C with anti-SETDB1 (kind gift from H.H. Ng reported previously [28]), anti-hnRNP K 3C2 (Abcam), anti-KAP1 20C1 (Abcam), anti-DYKDDDDK (FLAG, GenScript A00187–200), or rabbit or mouse IgG (Sigma-Aldrich I8140 and I8765). Whole-cell extracts were immunoprecipitated by adding antibodies and incubating overnight. Immunocomplexes were captured on protein A or protein G dynabeads (Life Technologies), washed three times in IP wash/whole-cell extraction buffer and eluted by boiling in SDS-PAGE loading buffer. For IP of FLAG-SETDB1 complexes from mESCs, *Setdb1* deletion was induced with tamoxifen in the 33#6 cell line expressing 3XFLAG-*Setdb1*, as previously [11]. As a negative control, the 33#6 line lacking the *Setdb1* transgene was used without inducing *Setdb1* deletion. Nuclear extracts were prepared as above with 10 mM NEM and immunoprecipitated overnight with anti-DYKDDDDK (FLAG) antibodies (GenScript). Immunocomplexes were captured on protein G dynabeads, washed with IP wash buffer containing 0.1% NP-40 and eluted with phosphate-buffered saline (Dulbecco) containing 0.1% Tween-20 and 500 μg/ml 3XFLAG peptide (Sigma Aldrich).

Western blotting was performed as previously described [42] using anti-SETDB1 H300 (Santa Cruz Biotechnology), anti-hnRNP K 3C2 (Abcam), anti-KAP1 20C1 (Abcam), anti-GAPDH (Millipore AB2302), anti-Ubc9 (Santa Cruz sc-5231), anti-SUMO1 (Santa Cruz sc-9060), anti-SENP1 (Novus Biologicals NB100–92101), anti-H3K9me2 (Abcam ab1221), anti-H3K9me3 (Active Motif 39161), anti-pan H3ac (Millipore 06–599), anti-H4 (Millipore 04–858), anti-GST (GenScript A00097–100), anti-DYKDDDDK (FLAG, GenScript) and anti-T7 (Millipore 59622). Primary antibodies were detected using IRDYE-conjugated secondary antibodies and scanning on the Odyssey imager (LiCOR Biosciences).

### Expression plasmids and recombinant proteins

The pSG5 plasmid harbouring FLAG-tagged mouse *Setdb1* cDNA [75] was a kind gift from L. Yang. The pcDNA3.3-T7-*HNRNPK* plasmid [32] was kindly provided by A. Srebrow. The pET16b-*HNRNPK* plasmid expressing 6X-His-tagged human hnRNP K was a kind gift from A. Ostareck-Lederer and was expressed and purified from the BL21 (DE3) *E.coli* strain as previously [76]. GST-tagged hnRNP K and GST-tagged KAP1 and KAP1^PxVxL^ (residues 379–524) were purchased from Novus Biologicals. GST-KAP1^PB^ (residues 624–811) was from Cayman Chemical. Purified GST was from Sigma-Aldrich, the C-terminal GST-RanGAP1 fragment (residues 419–587) was from Enzo Life Sciences and GST-p53 was from Millipore. Purified FLAG-tagged SETDB1 protein was from Active Motif.

### *In vitro* SUMOylation and GST pulldown assays

*In vitro* SUMOylation assays were performed according to previous methods [27] with minor modifications. Approximately 500 ng of GST-fused proteins were mixed with 125 ng Aos1/Uba2 heterodimer (Enzo Life Sciences BML-UW9330–0025), 500 ng Ubc9 (Enzo Life Sciences BML-UW9320–0100) and 2 μg 6X-His-tagged SUMO1 (Enzo Life Sciences ALX-201–045-C500) and incubated in 20 μl of 1X SUMOylation buffer (50 mM Tris pH 8.0, 50 mM KCl, 5 mM MgCl_2_, 1 mM DTT, 1 mM ATP) for 90 minutes at 30°C. Negative control reactions were performed by omitting SUMO1. Following this, reactions were stopped either by addition of SDS-PAGE loading buffer for western blotting or prepared for pulldown assays. For pulldown assays, GST-tagged proteins were immobilized on glutathione magnetic beads (GenScript), washed twice with pulldown buffer (50 mM Tris pH 8.0, 100 mM NaCl, 0.1 mM EDTA, 1 mM DTT, 0.01% Tween-20, 10% glycerol) incubated with 0.5–5 μg recombinant prey proteins (SETDB1, Ubc9 or hnRNP K) in 150 μl pulldown buffer for 1.5 h at 4°C. For pull-downs with GST-tagged KAP1 mutant baits and 6X-His-tagged hnRNP K prey, BSA was included in the binding reaction at 1 mg/ml. Beads were washed again three times pulldown buffer for SETDB1 and Ubc9 or in pulldown buffer containing 300 mM NaCl for hnRNP K and subsequently eluted with SDS-PAGE loading buffer for western blotting. Alternatively, glutathione elution buffer was used to elute bound proteins (50 mM Tris pH 8.0, 20 mM reduced L-glutathione, 1 mM DTT).

### Sucrose gradient sedimentation

Ultracentrifugation of proteins over sucrose gradients was performed according to previous methods [77, 78]. Approximately ~2 mg of mESC nuclear extract or ~4 μg of recombinant proteins in 500 μl was layered onto a 5 ml linear 5–50% gradient and centrifuged in parallel with identical gradients containing purified molecular weight standards (blue dextran 52.6S/~2 MDa, thyroglobulin 19.4S/670 kDa, catalase 11.4S/250 kDa, BSA 4.3S/67 kDa all from Sigma-Aldrich) at 27,500 rpm (~91,900 g) in a SW-55Ti rotor (Beckman Coulter) at 4°C for 18.5 h. Fractions of 200 μl were collected from top to bottom including the pellet fraction and 20 μl samples were assayed by western blot. Peaks for migration of the molecular weight standards were determined by absorbance at 280 nm.

### Chromatin immunoprecipitation

For native ChIP, mESCs were harvested by trypsinization and lysed in NChIP lysis buffer (20 mM HEPES pH 7.9, 50 mM KCl, 1 mM MgCl_2_, 3 mM CaCl_2_, 1 mM DTT, 0.5% NP-40, 10% glycerol) containing protease inhibitors on ice. Chromatin was digested with MNase (Worthington Biochemicals) to produce predominantly mono and di-nucleosomes and stopped by addition of EDTA and EGTA to 5 mM each, respectively. Salt concentration was adjusted to 150 mM KCl and native chromatin was immunoprecipitated overnight with anti-H3K9me3 (Active Motif 39161) or anti-H4K20me3 (Active motif 39180). Immunocomplexes were captured on protein A and G dynabeads (Life Technologies) washed extensively in RIPA buffer and eluted with 100 mM sodium bicarbonate buffer containing 1% SDS, 20 mM DTT. DNA was RNAse A-treated and purified over spin columns and analyzed by qPCR using primers indicated in S3 Table.

Crosslinked ChIP of SETDB1, KAP1 and SUMO1 was performed according to a previously described method [42] with or without 10 mM NEM. Chromatin was immunoprecipitated overnight at 4°C using anti-SETDB1 H300, anti-KAP1 20C1 or anti-SUMO1 (Santa Cruz sc-9060). ChIP for hnRNP K was performed according to a previous method with minor changes [51]. Briefly, cells were crosslinked in 1.45% formaldehyde for 15 minutes at room temperature, quenched with glycine and collected by centrifugation. Cells were lysed in modified RIPA (50 mM Tris pH 8.0, 150 mM NaCl, 1% NP-40, 0.5% triton-X-100, 5 mM EDTA, 1 mM DTT, 10 mM NEM, 10% glycerol containing protease inhibitors) and sonicated to yield predominantly 150–600 bp fragments. Chromatin lysate was precipitated overnight with 10 μg/ml anti-hnRNP K (Abcam ab70492). RNAse treatment of sonicated chromatin prior to ChIP was performed according to a previous method [79]. Samples were washed and eluted as described above and purified DNA was analyzed by quantitative PCR using ChIP primers indicated in S3 Table.

### Quantitative reverse-transcriptase PCR and RNA-seq analysis

Total RNA was extracted from mESCs with the GenElute RNA kit (Sigma-Aldrich) and reverse transcribed with SuperScript III (Life Technologies). Quantitative RT-PCR was performed as previously [42]. Expression levels were normalized to endogenous control genes *Gapdh* or β-actin (*Actb*). Primers used for qRT-PCR are listed in S3 Table. Strand-specific, paired-end mRNA-seq on poly(A) RNA was performed as previously described [42]. Libraries were sequenced on the Illumina HiSeq 2000. Reads per kilobase per million mapped reads (RPKM) was calculated and genes up- or downregulated relative to control siRNA KD cells were determined by applying fold-change threshold of 2 and minimum read count of 25 for genes up (down) regulated in hnRNP K KD (control) cell lines. Gene ontology analysis was performed with DAVID bioinformatic resource version 6.7 at http://david.abcc.ncifcrf.gov/home.jsp.

## Supporting Information

S1 Fig Identification of hnRNP K as a novel binding partner of SETDB1. (A) Silver stained gel and western blot of IgG and SETDB1 IP directly from TT2 mESC nuclear extract. Shown on right is the list of the top 10 nuclear proteins enriched >2-fold, with >2 unique peptides in the SETDB1 IP versus IgG IP detected by mass spectrometry. SETDB1 and known SETDB1-interacting protein MCAF1 are shown in red. “>10” for Medium/Light ratio indicates that there were no peptides detected in the Light (IgG IP) sample. (B) Co-IP assay of KAP1 and hnRNP K with SETDB1 from TT2 mESC nuclear extract in the presence of NEM, with or without 50 U/ml DNase I and 50 μg/ml RNase A. ‘NE’ represents ~10% of nuclear extract input. (C) Immunofluorescence staining of SETDB1 and hnRNP K in TT2 mESCs either untreated (-NEM) or incubated in 5 mM NEM for 30 min to block SENP activity in the cells prior to harvest. DNA is counterstained with Hoescht 33342. Merge is taken from all three stained images. Scale bar = 10 μm. (D) Western blot of sucrose gradient sedimentation (linear 5–50%) fractions for purified GST-KAP1 and GST-hnRNP K. Size standards were run in parallel: BSA = 4.3S/67 kDa, Thyroglobulin = 19.2S/670 kDa, Blue Dextran = 52.6S/2 MDa. (E) 5–50% linear sucrose gradient sedimentation as in (D) except of native mESC nuclear extracts prepared with or without NEM and analysed by western blot for SETDB1, KAP1 and hnRNP K. Density markers indicate peak positions of purified protein standards run in parallel, BSA = 4.3S/67 kDa, Catalase = 11.3S/250 kDa, Thyroglobulin = 19.2S/670 kDa, Blue Dextran = 52.6S/2 MDa. ‘P’ is the pellet fraction.
(TIF)

S2 Fig Analysis of interactions between SETDB1, KAP1 and hnRNP K. (A) Western blot analysis of KAP1 and hnRNP K in mESC nuclear extract where nuclei were isolated in 10 mM NEM and extracted with buffer containing 20 mM NEM (NE) and in a SETDB1 IP from the same extract. Slower migrating bands indicating SUMO-KAP1 were detected with KAP1 antibodies. Under these conditions, the majority of KAP1 proteins that are associated with SETDB1 are non-SUMOylated. (B) Co-IP assay of T7-tagged hnRNP K with FLAG-tagged SETDB1 upon in 293T cells either mock transfected (-) or transfected with the indicated expression constructs and subject to FLAG antibody IP at 48 h post-transfection. ‘IN’ represents 10% input whole-cell extract. Protein extract and IP were performed with 20 mM NEM. (C) Co-IP assay of KAP1 and SETDB1 with hnRNP K from TT2 whole-cell protein extracts either untransfected (Mock) or transfected with *Setdb1* siRNA at 24 h post-transfection. ‘Input’ represents 10% of whole-cell extract, GAPDH was a loading control. (D) Co-IP assay of endogenous KAP1 with hnRNP K from 293T whole-cell extracts prepared with 20 mM NEM. ‘IN’ represents 10% of whole cell extract, ‘IgG’ is the non-specific control IP. (E) Immunofluorescence staining of hnRNP K and KAP1 in mESCs. DNA is counterstained with Hoescht 33342. Merge is taken from the hnRNP K and KAP1 images only. Scale bar = 10 μm.
(TIF)

S3 Fig Knockdown of hnRNP K abolishes mESC proliferation, but minimally affects SSEA1 and Annexin V staining. (A) Western blot of hnRNP K in TT2 mESCs transfected with control or hnRNP K siRNA at 24 h post-transfection. GAPDH served as a loading control. (B) Growth curve of TT2 cells treated with control or *Hnrnpk* siRNA. Twenty-four hours after siRNA treatment, cells were seeded at 30,000 cells/well in a 24-well plate and viable (trypan blue-excluding) cells were counted every 24 h. Data are means (± s.d.) of three biological replicates. *p < 0.001, **p < 0.01, Student’s two-tailed T-test. (C) Cell cycle distributions in control and *Hnrnpk* siRNA transfected cells determined by flow cytometry at 72 h post-transfection. Approximately 10,000 cells were analyzed in each. (D) Percentages of SSEA1^+^ or Annexin V^-^ cells in PI^-^ populations of control or *Hnrnpk* siRNA-transfected cells at 72 h post-transfection. Approximately 10,000 PI^-^ cells were sampled in each.
(TIF)

S4 Fig Analysis of proviral de-repression upon KD of SETDB1, MCAF1 and hnRNP K. (A) qRT-PCR validation of *Setdb1* and *Mcaf1* mRNA knockdowns at 24 h post-transfection in *Setdb1^lox/-^*(33#6) MSCV-GFP cells. (B) Flow cytometry analysis of GFP^+^ cells of in the untransfected MSCV-GFP cells (-) or cells transfected with indicated siRNAs, at 72 h post-transfection. Data represent the percent of GFP^+^ of cells from a population of 10,000 viable PI^-^ cells. (C) Western blot analysis of SETDB1, hnRNP K and KAP1 in TT2 wt mESCs transfected with indicated siRNAs in two biological replicates per siRNA, at 24 h post-transfection. GAPDH was a loading control. (D) qRT-PCR analysis of intact class I MLV, class II IAP, MusD and MMERVK10C and class III MERVL and MTA ERVs in TT2 cells transfected with control, *Setdb1* or *Hnrnpk* siRNAs in two biological replicates each at 72 h post-transfection. Data are means of three technical replicates, error bars are s.d. (E) qRT-PCR analysis as in (D) except of class I and II ERV expression in J1 wt or *Dnmt* TKO cells transfected with indicated control or *Mcaf1* siRNA at 96 h post-transfection. (F) qRT-PCR analysis as in (E) except of *Oct4* expression at 96 h post-transfection in the indicated KD cultures from Fig. 3D.
(TIF)

S5 Fig RNA-seq analysis of *Hnrnpk* KD mESCs. (A) GO analysis from DAVID v6.7 of upregulated genes (264 total) in common between *Hnrnpk* KD biological replicates. (B) Table of 15 of the top 33 genes marked with SETDB1-dependent H3K9me3 (from Karimi et al. 2011) upregulated in both *Hnrnpk* KD and *Setdb1* KO cells. Fold-change data are derived from genic reads per kilobase per million mapped reads (RPKM) values and are ordered by magnitude of fold-change in *Setdb1* KO relative to corresponding control siRNA or TT2 wt cells for KD and KO, respectively. Highlighted in yellow are genes validated by qRT-PCR for upregulated expression and native ChIP for H3K9me3 in *Hnrnpk* KD cells, see also Fig. 4B and 4E. (C) Venn diagrams of the overlap between *Hnrnpk* KD RNA-seq upregulated genes (264) and *Kap1* KO (1300) RNA-seq from Rowe et al. (2010). p = 1.3×10^-36^, Fisher’s exact test (n = 22,138 ENSEMBL-annotated genes). (D) UCSC genome browser screenshot of the *Cml2* gene showing tracks from SETDB1 ChIP-seq in wt (Yuan et al. 2009) and H3K9me3 ChIP-seq from TT2 wt and *Setdb1* KO mESCs (Karimi et al. 2011) along with total coverage control siRNA and *Hnrnpk* siRNA RNA-seq and *Setdb1* wt and KO RNA-seq (Karimi et al. 2011). The *Cml2* promoter is downstream of an ETn family retroelement (ETnERV-int) that is marked by SETDB1-dependent H3K9me3. Numbers on the right indicate y-axis scale for each track.
(TIF)

S6 Fig hnRNP K and MCAF1 are required for H3K9me3 on proviral chromatin. (A) Crosslinked ChIP of TT2 chromatin +/- SENP inhibitor NEM with IgG, KAP1 or SUMO1 antibodies. Data are mean enrichment relative to input of three technical replicates, error bars are s.d. (B) Crosslinked ChIP of hnRNP K from TT2 chromatin extracts untreated or treated with RNase A/T1 mix (see Methods). Although less efficient, there was no change in hnRNP K enrichment at these ERVs upon RNase treatment. (C) Native ChIP for H3K9me3 in TT2 wt mESCs transfected with the indicated siRNAs at 48 h post-transfection. (D) Native ChIP for H3K9me3 at ERV 5’LTRs, MSCV 5’ LTR and the *Myc* core promoter and TSS (-50 to +50) on unsorted (GFP^+^ and GFP^-^) MSCV-GFP cells transfected with the indicated siRNAs at 72 h post-transfection. (E) Western blot of H3K9me3, H3K9me2 and total H4 on acid-extracted histones from TT2 wt cells transfected with indicated siRNAs, the same cells as in Fig. 5C.
(TIF)

S7 Fig hnRNP K but not MCAF1 is required for SETDB1 recruitment to proviral chromatin. (A) Crosslinked ChIP for SETDB1 on unsorted (GFP^+^ and GFP^-^) MSCV-GFP cells transfected with control or *Hnrnpk* siRNAs at 72 h post-transfection. All data are mean enrichment relative to input of three technical replicates, error bars are s.d. *Ifna5* core promoter and TSS (-50 to +50) was a negative control locus. (B) Crosslinked ChIP for SETDB1 on unsorted MSCV-GFP cells as in (A), except transfected with control, *Setdb1* or *Mcaf1* siRNAs at 72 h post-transfection.(C)Immunofluorescence staining of hnRNP K and SETDB1 in TT2 cells at transfected with control or *Hnrnpk* siRNAs at 72 h post-transfection. DNA was counterstained with Hoescht 33342. Scale bar = 20 μm.
(TIF)

S8 Fig SUMO1 ChIP analysis in Ubc9, hnRNP K and SETDB1 KD cells. (A) Crosslinked ChIP of SUMO1 from unsorted (GFP^+^ and GFP^-^) MSCV-GFP cells transfected with control and *Ubc9* siRNAs at 48 h post-transfection. N.D. = not detected in 40 cycles. Data are mean enrichment as a percent of input chromatin from three technical replicates, error bars are s.d. (B) Crosslinked ChIP as in (A) except on unsorted MSCV-GFP cells transfected with control or *Hnrnpk* siRNAs at 72 h post-transfection. (C) Crosslinked ChIP as in (A) except on TT2 cells transfected with control or *Hnrnpk* siRNAs at 72 h post-transfection. (D) Flow cytometry of MSCV-GFP cells transfected with control or *Setdb1* siRNAs at 72 h post-transfection. Data are percent of GFP^+^ cells in a population of 10,000 PI-negative viable cells for each. Inset, qRT-PCR of *Setdb1* transcripts in MSCV-GFP cells transfected with control or *Setdb1* siRNAs at 24 h post-transfection. Data are mean fold-change from three technical replicates, normalized siCtrl, relative to level of *Gapdh* transcripts. Error bars are s.d. (E) Crosslinked SUMO1 ChIP as in (A) except in the control or *Setdb1* KD MSCV-GFP cells at 72 h post-transfection.
(TIF)

S1 Table Genes misregulated (≥2-fold upregulated or ≥50% downregulated) in common between hnRNP K KD biological replicates and expression of ERVs in hnRNP K KD cells. (PDF)

S2 Table Genes upregulated (≥2-fold) in common among hnRNP K KD, *Setdb1* KO and *Kap1* KO mESCs. (PDF)

S3 Table List of PCR primers used in this study. (PDF)
