# Correction: hnRNP K Coordinates Transcriptional Silencing by SETDB1 in Embryonic Stem Cells

**DOI:** 10.1371/journal.pgen.1006390

**Published:** 2016-10-14

**Authors:** Peter J. Thompson, Vered Dulberg, Kyung-Mee Moon, Leonard J. Foster, Carol Chen, Mohammad M. Karimi, Matthew C. Lorincz

The Data Availability statement for this paper does not include a GEO accession number for the RNA-seq data presented. These data are available from the GEO database (http://www.ncbi.nlm.nih.gov/geo/query/acc.cgi?acc=GSE84386). The complete statement is:

The RNA-seq data are available from the GEO database (http://www.ncbi.nlm.nih.gov/geo/query/acc.cgi?acc=GSE84386). All other relevant data are within the paper and its Supporting Information files.
