# Supplementary figures and images for: hnRNP K Coordinates Transcriptional Silencing by SETDB1 in Embryonic Stem Cells
Source: PLoS Genet. 2015 Jan 22;11(1):e1004933. doi: 10.1371/journal.pgen.1004933 (PMC4303303; doi:10.1371/journal.pgen.1004933)

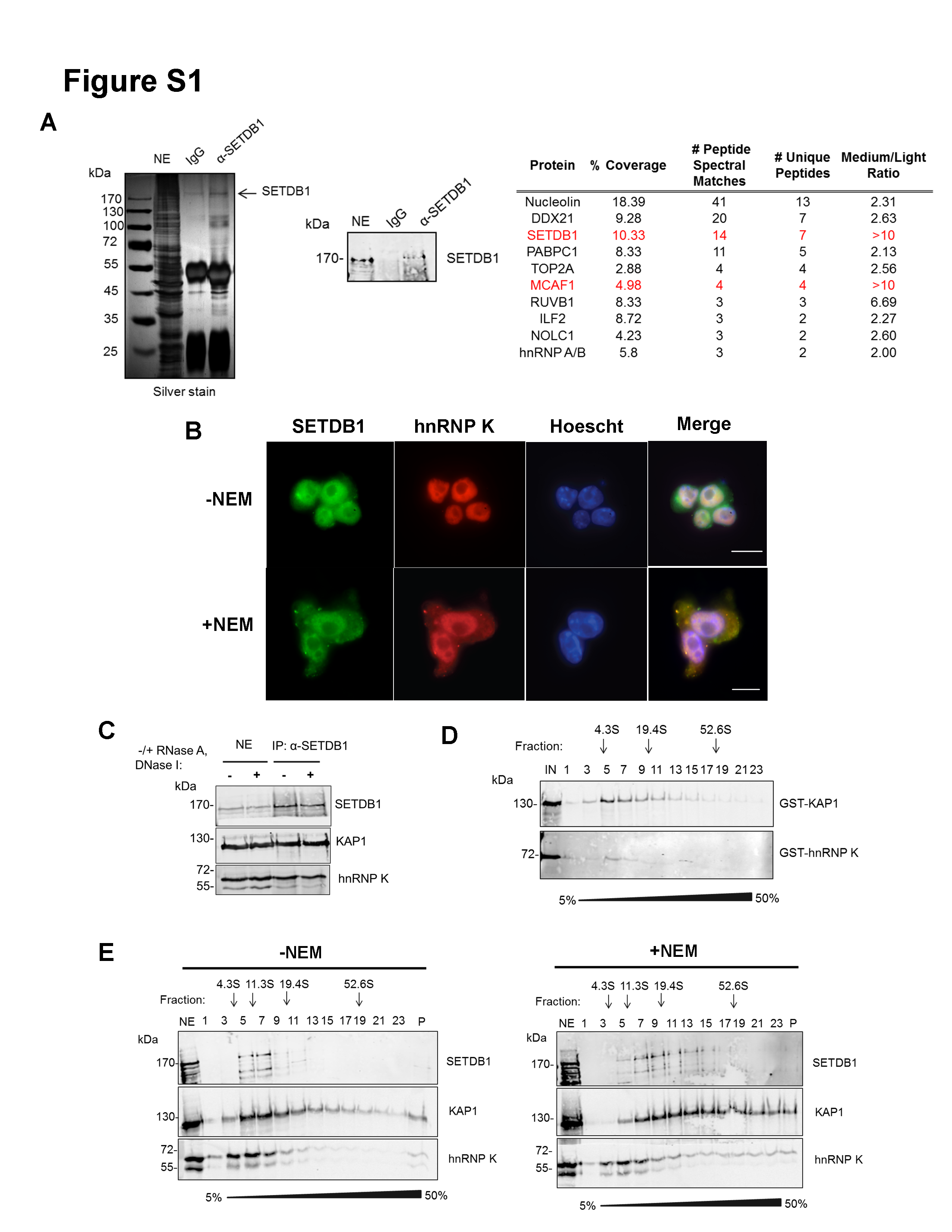

Supplement: S1 Fig — (A) Silver stained gel and western blot of IgG and SETDB1 IP directly from TT2 mESC nuclear extract. Shown on right is the list of the top 10 nuclear proteins enriched >2-fold, with >2 unique peptides in the SETDB1 IP versus IgG IP detected by mass spectrometry. SETDB1 and known SETDB1-interacting protein MCAF1 are shown in red. “>10” for Medium/Light ratio indicates that there were no peptides detected in the Light (IgG IP) sample. (B) Co-IP assay of KAP1 and hnRNP K with SETDB1 from TT2 mESC nuclear extract in the presence of NEM, with or without 50 U/ml DNase I and 50 μg/ml RNase A. ‘NE’ represents ~10% of nuclear extract input. (C) Immunofluorescence staining of SETDB1 and hnRNP K in TT2 mESCs either untreated (-NEM) or incubated in 5 mM NEM for 30 min to block SENP activity in the cells prior to harvest. DNA is counterstained with Hoescht 33342. Merge is taken from all three stained images. Scale bar = 10 μm. (D) Western blot of sucrose gradient sedimentation (linear 5–50%) fractions for purified GST-KAP1 and GST-hnRNP K. Size standards were run in parallel: BSA = 4.3S/67 kDa, Thyroglobulin = 19.2S/670 kDa, Blue Dextran = 52.6S/2 MDa. (E) 5–50% linear sucrose gradient sedimentation as in (D) except of native mESC nuclear extracts prepared with or without NEM and analysed by western blot for SETDB1, KAP1 and hnRNP K. Density markers indicate peak positions of purified protein standards run in parallel, BSA = 4.3S/67 kDa, Catalase = 11.3S/250 kDa, Thyroglobulin = 19.2S/670 kDa, Blue Dextran = 52.6S/2 MDa. ‘P’ is the pellet fraction. (TIF) [file pgen.1004933.s001.tif]

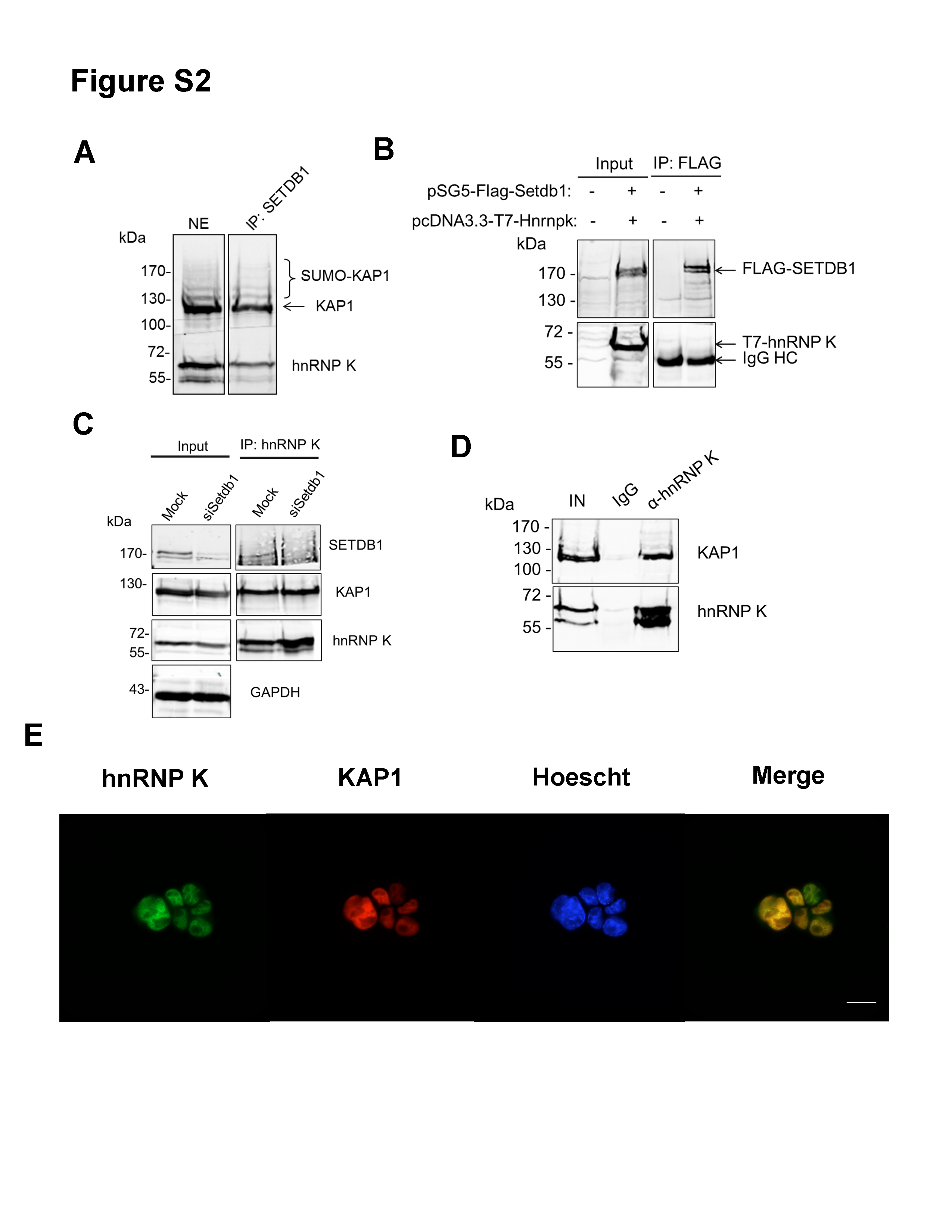

Supplement: S2 Fig — (A) Western blot analysis of KAP1 and hnRNP K in mESC nuclear extract where nuclei were isolated in 10 mM NEM and extracted with buffer containing 20 mM NEM (NE) and in a SETDB1 IP from the same extract. Slower migrating bands indicating SUMO-KAP1 were detected with KAP1 antibodies. Under these conditions, the majority of KAP1 proteins that are associated with SETDB1 are non-SUMOylated. (B) Co-IP assay of T7-tagged hnRNP K with FLAG-tagged SETDB1 upon in 293T cells either mock transfected (-) or transfected with the indicated expression constructs and subject to FLAG antibody IP at 48 h post-transfection. ‘IN’ represents 10% input whole-cell extract. Protein extract and IP were performed with 20 mM NEM. (C) Co-IP assay of KAP1 and SETDB1 with hnRNP K from TT2 whole-cell protein extracts either untransfected (Mock) or transfected with Setdb1 siRNA at 24 h post-transfection. ‘Input’ represents 10% of whole-cell extract, GAPDH was a loading control. (D) Co-IP assay of endogenous KAP1 with hnRNP K from 293T whole-cell extracts prepared with 20 mM NEM. ‘IN’ represents 10% of whole cell extract, ‘IgG’ is the non-specific control IP. (E) Immunofluorescence staining of hnRNP K and KAP1 in mESCs. DNA is counterstained with Hoescht 33342. Merge is taken from the hnRNP K and KAP1 images only. Scale bar = 10 μm. (TIF) [file pgen.1004933.s002.tif]

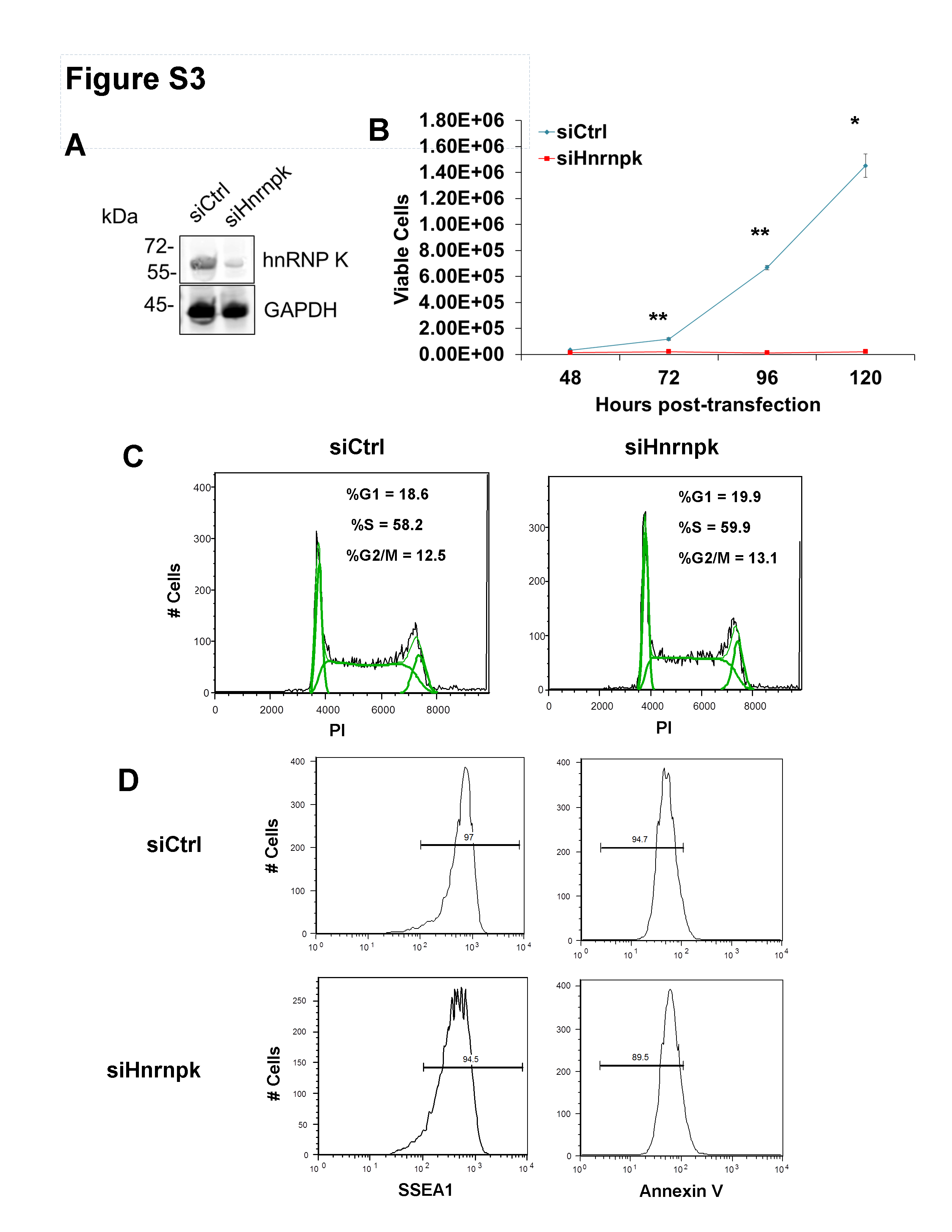

Supplement: S3 Fig — (A) Western blot of hnRNP K in TT2 mESCs transfected with control or hnRNP K siRNA at 24 h post-transfection. GAPDH served as a loading control. (B) Growth curve of TT2 cells treated with control or Hnrnpk siRNA. Twenty-four hours after siRNA treatment, cells were seeded at 30,000 cells/well in a 24-well plate and viable (trypan blue-excluding) cells were counted every 24 h. Data are means (± s.d.) of three biological replicates. *p < 0.001, **p < 0.01, Student’s two-tailed T-test. (C) Cell cycle distributions in control and Hnrnpk siRNA transfected cells determined by flow cytometry at 72 h post-transfection. Approximately 10,000 cells were analyzed in each. (D) Percentages of SSEA1+ or Annexin V- cells in PI- populations of control or Hnrnpk siRNA-transfected cells at 72 h post-transfection. Approximately 10,000 PI- cells were sampled in each. (TIF) [file pgen.1004933.s003.tif]

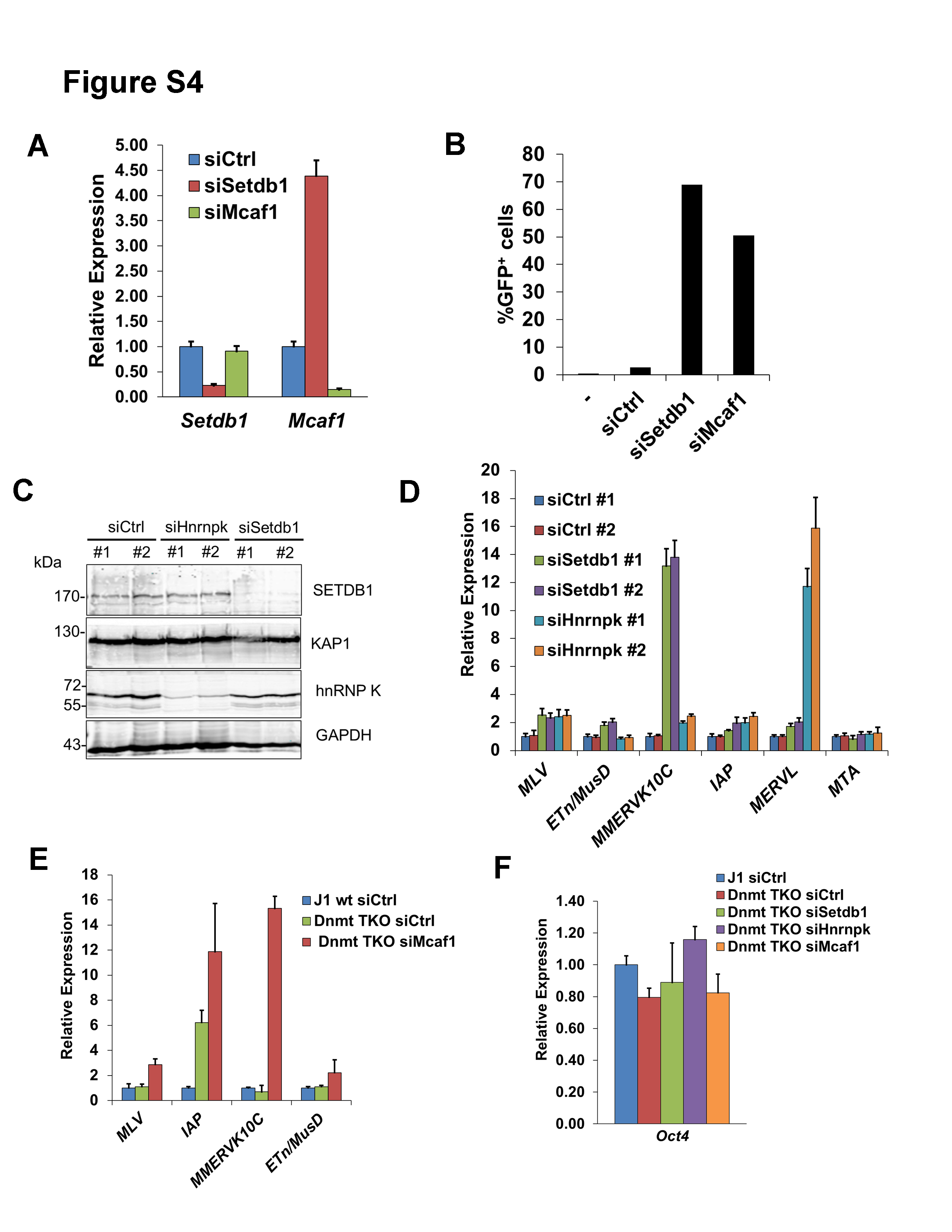

Supplement: S4 Fig — (A) qRT-PCR validation of Setdb1 and Mcaf1 mRNA knockdowns at 24 h post-transfection in Setdb1lox/-(33#6) MSCV-GFP cells. (B) Flow cytometry analysis of GFP+ cells of in the untransfected MSCV-GFP cells (-) or cells transfected with indicated siRNAs, at 72 h post-transfection. Data represent the percent of GFP+ of cells from a population of 10,000 viable PI- cells. (C) Western blot analysis of SETDB1, hnRNP K and KAP1 in TT2 wt mESCs transfected with indicated siRNAs in two biological replicates per siRNA, at 24 h post-transfection. GAPDH was a loading control. (D) qRT-PCR analysis of intact class I MLV, class II IAP, MusD and MMERVK10C and class III MERVL and MTA ERVs in TT2 cells transfected with control, Setdb1 or Hnrnpk siRNAs in two biological replicates each at 72 h post-transfection. Data are means of three technical replicates, error bars are s.d. (E) qRT-PCR analysis as in (D) except of class I and II ERV expression in J1 wt or Dnmt TKO cells transfected with indicated control or Mcaf1 siRNA at 96 h post-transfection. (F) qRT-PCR analysis as in (E) except of Oct4 expression at 96 h post-transfection in the indicated KD cultures from Fig. 3D. (TIF) [file pgen.1004933.s004.tif]

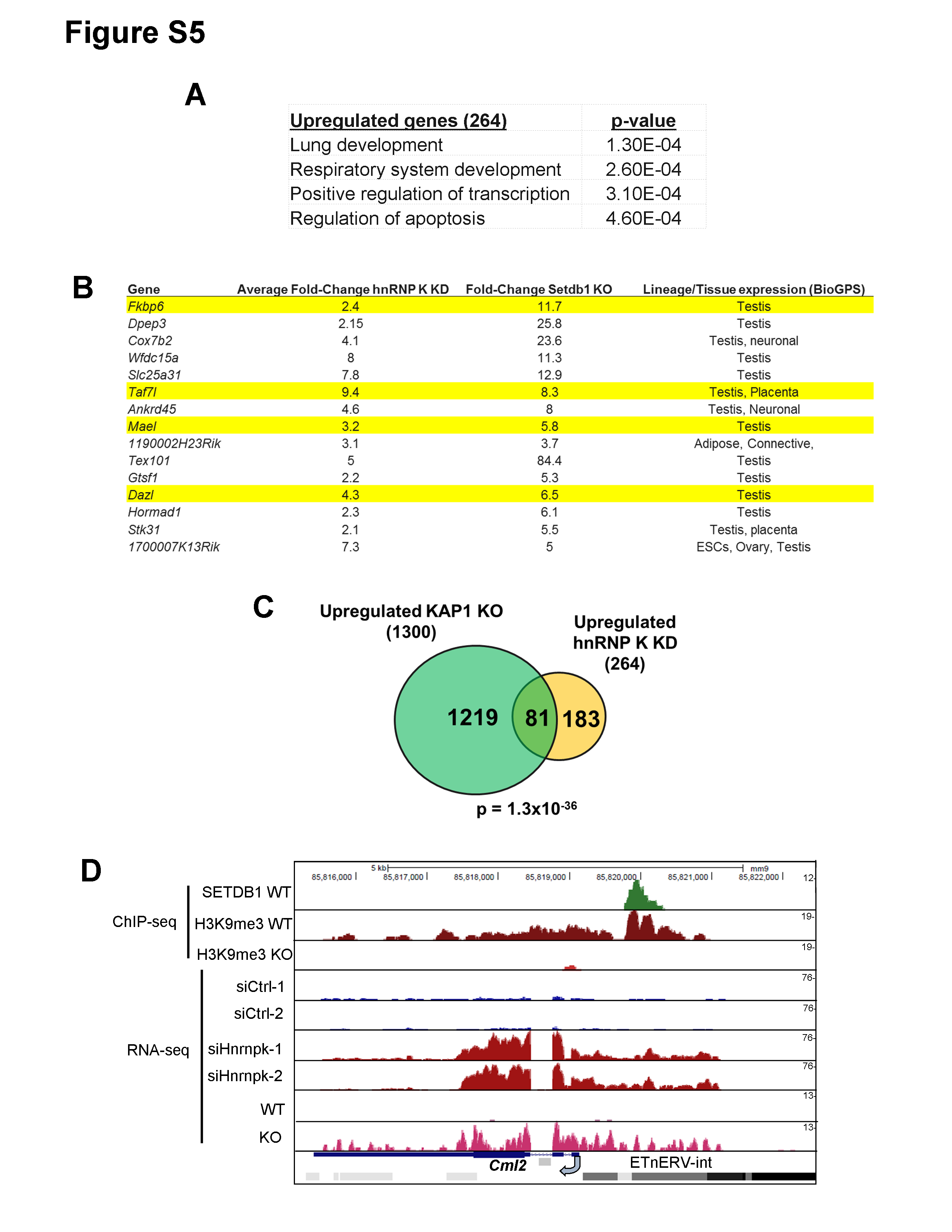

Supplement: S5 Fig — (A) GO analysis from DAVID v6.7 of upregulated genes (264 total) in common between Hnrnpk KD biological replicates. (B) Table of 15 of the top 33 genes marked with SETDB1-dependent H3K9me3 (from Karimi et al. 2011) upregulated in both Hnrnpk KD and Setdb1 KO cells. Fold-change data are derived from genic reads per kilobase per million mapped reads (RPKM) values and are ordered by magnitude of fold-change in Setdb1 KO relative to corresponding control siRNA or TT2 wt cells for KD and KO, respectively. Highlighted in yellow are genes validated by qRT-PCR for upregulated expression and native ChIP for H3K9me3 in Hnrnpk KD cells, see also Fig. 4B and 4E. (C) Venn diagrams of the overlap between Hnrnpk KD RNA-seq upregulated genes (264) and Kap1 KO (1300) RNA-seq from Rowe et al. (2010). p = 1.3×10^-36^, Fisher’s exact test (n = 22,138 ENSEMBL-annotated genes). (D) UCSC genome browser screenshot of the Cml2 gene showing tracks from SETDB1 ChIP-seq in wt (Yuan et al. 2009) and H3K9me3 ChIP-seq from TT2 wt and Setdb1 KO mESCs (Karimi et al. 2011) along with total coverage control siRNA and Hnrnpk siRNA RNA-seq and Setdb1 wt and KO RNA-seq (Karimi et al. 2011). The Cml2 promoter is downstream of an ETn family retroelement (ETnERV-int) that is marked by SETDB1-dependent H3K9me3. Numbers on the right indicate y-axis scale for each track. (TIF) [file pgen.1004933.s005.tif]

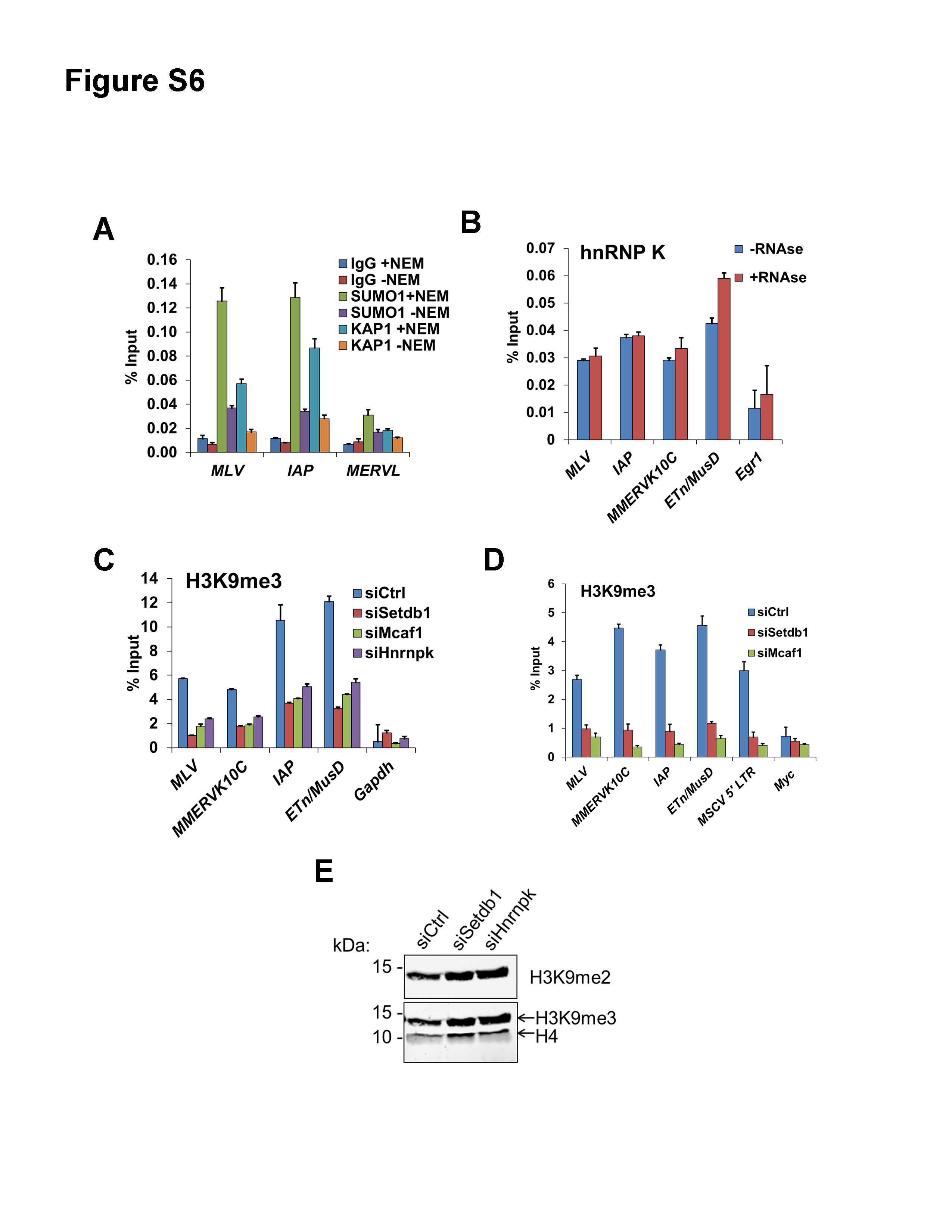

Supplement: S6 Fig — (A) Crosslinked ChIP of TT2 chromatin +/- SENP inhibitor NEM with IgG, KAP1 or SUMO1 antibodies. Data are mean enrichment relative to input of three technical replicates, error bars are s.d. (B) Crosslinked ChIP of hnRNP K from TT2 chromatin extracts untreated or treated with RNase A/T1 mix (see Methods). Although less efficient, there was no change in hnRNP K enrichment at these ERVs upon RNase treatment. (C) Native ChIP for H3K9me3 in TT2 wt mESCs transfected with the indicated siRNAs at 48 h post-transfection. (D) Native ChIP for H3K9me3 at ERV 5’LTRs, MSCV 5’ LTR and the Myc core promoter and TSS (-50 to +50) on unsorted (GFP+ and GFP-) MSCV-GFP cells transfected with the indicated siRNAs at 72 h post-transfection. (E) Western blot of H3K9me3, H3K9me2 and total H4 on acid-extracted histones from TT2 wt cells transfected with indicated siRNAs, the same cells as in Fig. 5C. (TIF) [file pgen.1004933.s006.tif]

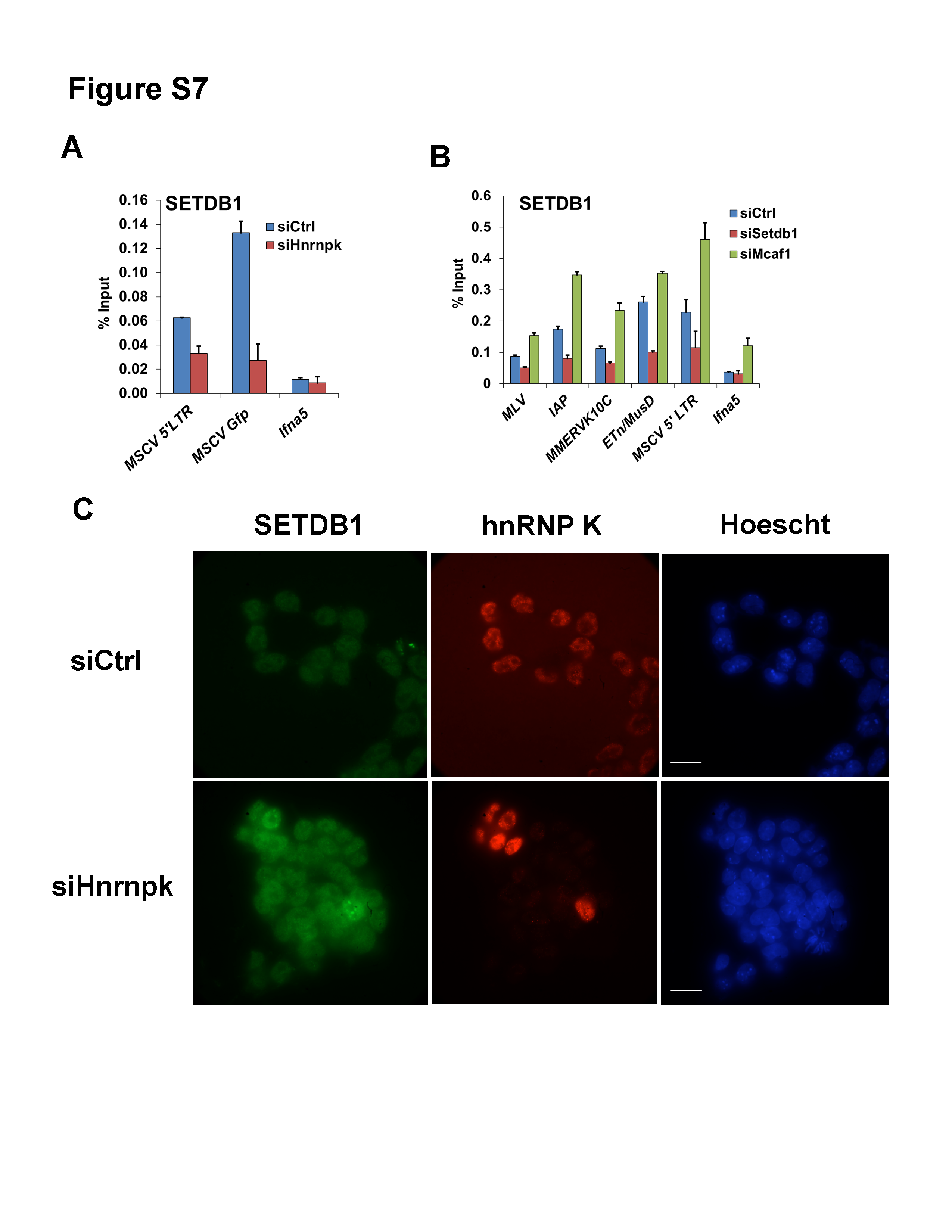

Supplement: S7 Fig — (A) Crosslinked ChIP for SETDB1 on unsorted (GFP+ and GFP-) MSCV-GFP cells transfected with control or Hnrnpk siRNAs at 72 h post-transfection. All data are mean enrichment relative to input of three technical replicates, error bars are s.d. Ifna5 core promoter and TSS (-50 to +50) was a negative control locus. (B) Crosslinked ChIP for SETDB1 on unsorted MSCV-GFP cells as in (A), except transfected with control, Setdb1 or Mcaf1 siRNAs at 72 h post-transfection.(C)Immunofluorescence staining of hnRNP K and SETDB1 in TT2 cells at transfected with control or Hnrnpk siRNAs at 72 h post-transfection. DNA was counterstained with Hoescht 33342. Scale bar = 20 μm. (TIF) [file pgen.1004933.s007.tif]

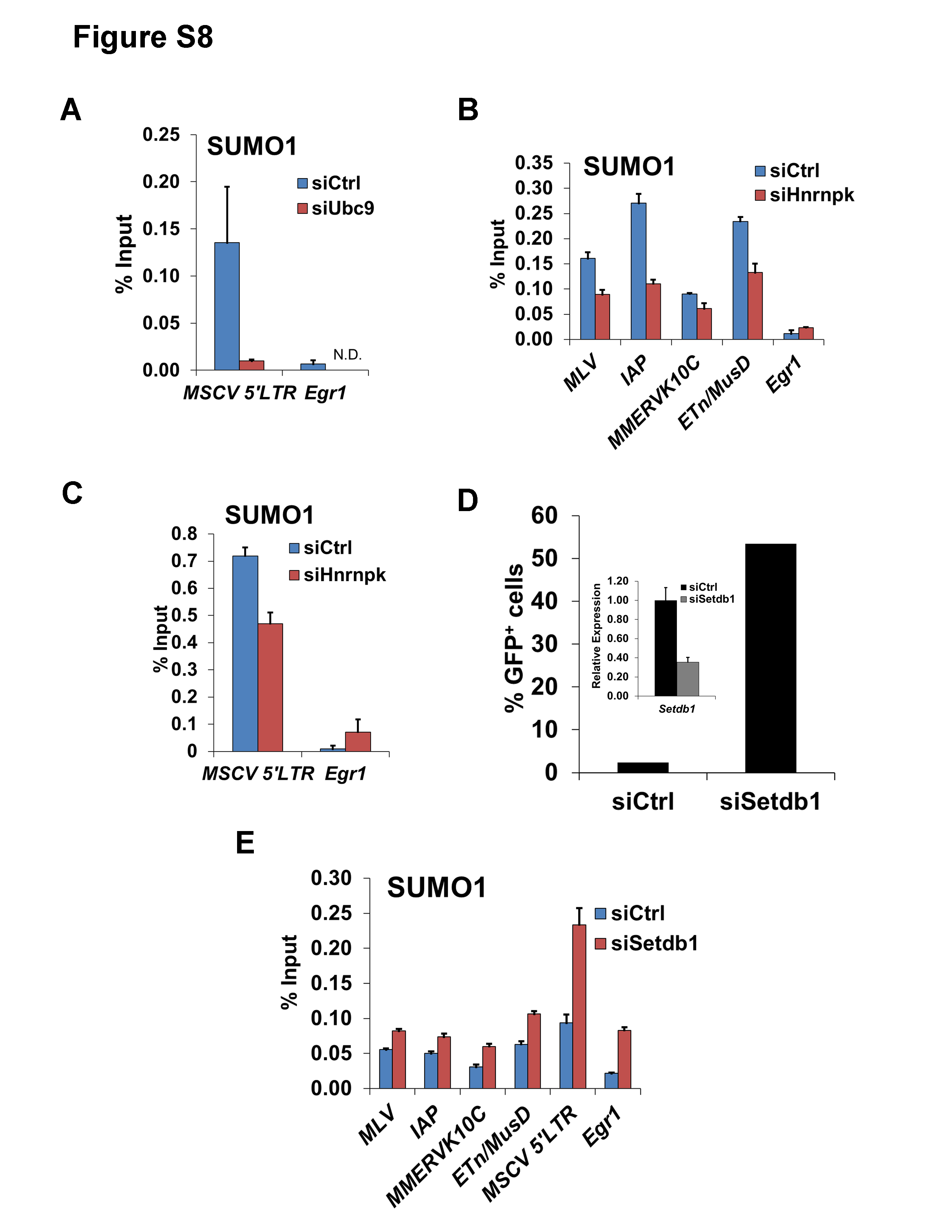

Supplement: S8 Fig — (A) Crosslinked ChIP of SUMO1 from unsorted (GFP+ and GFP-) MSCV-GFP cells transfected with control and Ubc9 siRNAs at 48 h post-transfection. N.D. = not detected in 40 cycles. Data are mean enrichment as a percent of input chromatin from three technical replicates, error bars are s.d. (B) Crosslinked ChIP as in (A) except on unsorted MSCV-GFP cells transfected with control or Hnrnpk siRNAs at 72 h post-transfection. (C) Crosslinked ChIP as in (A) except on TT2 cells transfected with control or Hnrnpk siRNAs at 72 h post-transfection. (D) Flow cytometry of MSCV-GFP cells transfected with control or Setdb1 siRNAs at 72 h post-transfection. Data are percent of GFP+ cells in a population of 10,000 PI-negative viable cells for each. Inset, qRT-PCR of Setdb1 transcripts in MSCV-GFP cells transfected with control or Setdb1 siRNAs at 24 h post-transfection. Data are mean fold-change from three technical replicates, normalized siCtrl, relative to level of Gapdh transcripts. Error bars are s.d. (E) Crosslinked SUMO1 ChIP as in (A) except in the control or Setdb1 KD MSCV-GFP cells at 72 h post-transfection. (TIF) [file pgen.1004933.s008.tif]
