# Supplementary material for: hnRNP K Coordinates Transcriptional Silencing by SETDB1 in Embryonic Stem Cells
Source: PLoS Genet. 2015 Jan 22;11(1):e1004933. doi: 10.1371/journal.pgen.1004933 (PMC4303303; doi:10.1371/journal.pgen.1004933)
Supplement: S1 Table — (PDF) [file pgen.1004933.s009.pdf]

Genes upregulated in common in hnRNP K KD biological replicates (264)

Ngfr
Clec10a
Ccl3
Ckb
Nkx2-1
Sfpi1
Cd97
Apoe
Hspb1
Casp14
Hmox1
Gata6
Calcoco2
Ctsd
Sertad1
Kcnq1
Taf7l
Dazl
Gadd45b
Gata3
Lama5
Col20a1
Sdc4
Pltp
Mapt
Tbx3
Cd68
Atp6ap1
Perp
Mgat4c
Ctgf
Mdm2
Phlda1
Ccng1
Btg2
Id2
Arg2
Fbp2
Plk2
Anxa8
1190002H23Rik
Dab2
Alcam

Pros1
Zfp641
Cdkn1a
Cpn2
Cndp2
Ankrd22
Dkk1
Cpt1a
Gsto1
Hexa
Cd63
Ddit3
Eps8l2
Pramel6
Pdgfa
Tcfcp2l1
Cd55
Chit1
1700007K13Rik
Dpp7
Nkx2-2
8030411F24Rik
Cst9
Mfsd1
Mme
Tacr3
Wls
Cyr61
Trp53inp1
Aqp3
4930579C15Rik
Laptm5
Fabp3
Tinagl1
Gpatch3
Abcb1b
Trp73
Lrpap1
Agpat9
Slc6a12
Tead4
Mfge8
Spns1
Dkkl1
Tpp1
Bgn

Renbp
Lamp1
Col4a1
Col4a2
Slc25a4
Ces2e
Gabarapl2
Tbx20
Cryab
1600029D21Rik
Ankk1
Rbp2
Glt25d2
Wnt6
Crlf2
Cml2
Cfhr2
Tmem132c
AI661453
Grn
Rtkn
B020004J07Rik
A430110N23Rik
Foxa1
Colec11
Pramel5
Gm12800
Nodal
Vnn1
Atg14
Kcnk2
Satb2
Pmepa1
Gdf15
Sct
Tex19.1
Tnrc18
Fam190a
Cpeb2
Cited2
Havcr1
Timd2
Ltb4r2
Matn1
Mael
Erf
Rnasek

Phlda3
Svop
4933440N22Rik
S100a13
Snai1
Dub3
Gm14492
Ly6k
Phf11
Ankrd45
Cirbp
Dcaf12l1
Chrm3
Gm13078
Gprc5a
8030474K03Rik
Ddit4l
Spz1
H2-M10.4
Igf2
Lgals3
Ftl1
Ptges
Islr2
AU021092
Wfdc15a
C130026I21Rik
Krt15
Zscan4c
Dub1
Gm9982
Foxi3
Dub2a
Gm15698
Olfr787
Tdpoz3
Nkx2-9
Zfp353
Usp17l5
Tdpoz1
Tdpoz4
Gm8994
Olfr815
Dub1a
Tex101
Gm6406
Slc39a4

Gm5576
Arl9
Olfr376
Gm11232
Iqcf1
Gm13083
Cyp2b19
Trim43a
Tlx2
Aard
Flnc
Slc25a31
Zbtb10
Fbp1
Ahnak
Sp110
BC080695
Gm13119
Zscan4-ps2
Zscan4f
Gm12794
Zfp352
Gm428
BB287469
Abcb5
Ahnak2
Gm6468
Gm10424
Gm5662
Nup62cl
C4b
AA792892
Gm6502
Gm16522
Gm7647
Gm10526
Gm10527
Gm10528
Btbd19
Gm6509
Gm3147
Gm7682
Gm10696
Gm4858
Ubtfl1
Bhmt
Gm10775

Gm10807
Bex6
Tmem92-ps
Ass1
Gm11564
Gm13128
Pramef17
Gm13109
Pramef6
Gm2016
Gm2046
Gm8300
C86695
Trim43b
Glb1
Gm5698
Gm5699
Gm4778
Acad11
Gm16478
Gm7957
Gm6189
Zscan4d
Gm4340
Gm4840
Vgll3
Gm8766
Zscan4b
Gm4902
Gm2075
Zscan4e
Gm8038
Gm4027
Gm6804

Genes downregulated in common between hnRNP K KD biological replicates (26)

Pdk1
Lphn1
Cenpv
Rad50
Hnrnpk
Tpi1
Pycr1
Scd2
Ifitm1
Pecr
Tuba4a
Plac8
Mt3
Nedd4
Scd1
Egr1
Foxk2
Bag2
Phgdh
U2af1
Tubb4
Ldha
Gm7964
Slfn9
Cisd3
2310009B15Rik

TE family	Number of elements	siCtrl #1 RNA-seq (RPKM)	siCtrl #2 RNA-seq (RPKM)	Hnrnpk KD #1 RNA-seq (RPKM)	Hnrnpk KD #2 RNA-seq (RPKM)	Fold Change (Hnrnpk KD #1/siCtrl #1)	Fold Change (Hnrnpk KD #2/siCtrl #2)
LTR:ERVL:MERVL-int	2599	0.013	0.015	0.217	0.442	16.661	30.143
LTR:ERVL:MT2_Mm	2671	0.016	0.018	0.221	0.463	14.206	26.328
LTR:ERV1:MER67D	462	0.000	0.000	0.001	0.002	10.861	16.131
LTR:ERVK:ETnERV3-int	945	0.005	0.006	0.019	0.026	4.043	4.161
LTR:ERV1:RLTR6_Mm	1202	0.014	0.016	0.052	0.073	3.764	4.667
LTR:ERVK:RLTR13B2	320	0.008	0.010	0.029	0.046	3.479	4.823
LTR:Gypsy?:LTR85a	236	0.009	0.008	0.030	0.017	3.457	2.082
LTR:ERVL:LTR84b	185	0.012	0.011	0.039	0.063	3.328	5.776
LTR:Genie?:LTR88c	126	0.001	0.003	0.002	0.016	3.254	5.357
LTR:ERVL:MER70B	257	0.001	0.003	0.004	0.003	3.200	0.885
LTR:ERVL:LTR80B	113	0.001	0.002	0.003	0.002	3.200	0.860
LTR:ERV1:MER67C	617	0.002	0.002	0.005	0.004	2.987	1.801
LTR:ERV1:RLTR4_MM-int	502	0.034	0.034	0.095	0.316	2.794	9.172
LTR:ERVL:LTR75	105	0.005	0.008	0.014	0.020	2.760	2.589
LTR:MaLR:MLT1E1	581	0.003	0.003	0.008	0.003	2.619	1.041
LTR:ERVK:RLTR13A	377	0.013	0.015	0.034	0.036	2.567	2.384
LINE:L1:L1MEb	375	0.031	0.036	0.079	0.005	2.520	0.128
LTR:MaLR:MTE2b-int	725	0.009	0.010	0.021	0.021	2.363	2.153
LTR:ERV1:LTR68	112	0.004	0.006	0.009	0.005	2.337	0.811
LTR:Gypsy?:LTR85c	128	0.003	0.002	0.007	0.029	2.337	11.957
LINE:L1:L1M6	370	0.002	0.002	0.004	0.003	2.327	1.658
LTR:ERVK:IAPLTR4_I	246	0.000	0.001	0.001	0.003	2.326	5.488
LTR:ERV1:RLTR4_Mm	222	0.196	0.217	0.452	0.701	2.302	3.235
LTR:ERVK:RLTR19	1023	0.030	0.029	0.068	0.053	2.302	1.825
LTR:Gypsy:MamGypLTR1b	145	0.009	0.008	0.021	0.011	2.297	1.278
LTR:ERVK:RNLTR23	503	0.002	0.004	0.005	0.005	2.252	1.306
LTR:MaLR:ORR1A3-int	2455	0.025	0.026	0.056	0.095	2.241	3.657
LTR:ERVK:RLTR13D4	276	0.007	0.006	0.016	0.012	2.197	1.864

LTR:ERVK:RLTR13D5	705	0.006	0.006	0.013	0.013	2.168	2.107
LTR:ERV1:RMER21B	1262	0.010	0.010	0.022	0.026	2.114	2.614
LTR:MaLR:MLT1C-int	127	0.010	0.010	0.020	0.000	2.067	0.038
LTR:ERV1:LTR31	199	0.006	0.010	0.011	0.008	2.018	0.820
LINE:L1:L1Md_T	23234	0.007	0.008	0.015	0.023	2.010	2.841
LINE:L1:L1MEe	1227	0.062	0.064	0.121	0.007	1.974	0.105
LTR:ERVK:RLTR44A	240	0.037	0.037	0.072	0.003	1.964	0.090
LTR:ERVK:RLTR26	2048	0.011	0.012	0.021	0.021	1.940	1.717
LTR:ERVK:RLTR19B	228	0.006	0.007	0.012	0.016	1.935	2.202
LTR:ERVK:RMER17D	1178	0.066	0.063	0.125	0.130	1.913	2.066
LINE:L1:L1M	470	0.008	0.008	0.015	0.012	1.907	1.555
LTR:ERVL:ERVL-E-int	1116	0.009	0.009	0.018	0.003	1.894	0.336
LTR:ERVK:RLTR10D	882	0.007	0.007	0.014	0.036	1.872	5.236
LTR:ERVL:MLT2D	1564	0.004	0.004	0.007	0.012	1.834	2.954
LTR:ERVL:MER70A	181	0.003	0.004	0.006	0.003	1.833	0.737
LINE:L1:MusHAL1	5895	0.003	0.003	0.005	0.005	1.833	1.463
LTR:ERV1:RLTR1D	511	0.011	0.011	0.020	0.030	1.823	2.791
LTR:MaLR:MLT1J-int	509	0.004	0.005	0.008	0.003	1.814	0.565
LINE:L1:L1Md_Gf	994	0.004	0.005	0.008	0.021	1.809	4.574
LINE:L1:Lx2A	1394	0.001	0.001	0.002	0.002	1.802	2.770
LTR:ERVL:MER73	136	0.001	0.002	0.002	0.001	1.802	0.455
LTR:ERVK:RLTR13D6	1237	0.005	0.006	0.010	0.011	1.787	1.935
LINE:L1:L1M3de	170	0.027	0.027	0.048	0.001	1.777	0.043
LTR:MaLR:ORR1D2-int	1364	0.009	0.010	0.015	0.018	1.771	1.827
LTR:ERVK:IAPEz-int	7826	0.018	0.017	0.032	0.053	1.762	3.070
LTR:ERVK:RLTR42-int	2453	0.006	0.006	0.011	0.058	1.741	9.636
LTR:ERVK:RMER20A	1693	0.005	0.006	0.009	0.024	1.734	4.057
LTR:ERV1:RLTR47_MM	663	0.013	0.015	0.023	0.032	1.724	2.172
LTR:ERVK:MurERV4_19-int	988	0.003	0.003	0.005	0.035	1.722	11.348
LTR:ERVK:IAPEy-int	727	0.001	0.001	0.002	0.002	1.717	1.353
LTR:ERV1:MER52-int	199	0.004	0.006	0.006	0.015	1.708	2.475
LTR:ERVK:MMERVK10C-int	3437	0.008	0.009	0.014	0.021	1.706	2.273

LTR:ERVL:MLT2C1	870	0.003	0.003	0.006	0.004	1.697	1.276
LINE:L1:L1M1	927	0.008	0.007	0.013	0.004	1.694	0.586
LTR:ERVK:IAPEY_LTR	1087	0.002	0.002	0.003	0.007	1.693	3.022
SINE:Alu:AluG_3	282	0.229	0.246	0.387	0.413	1.688	1.679
LTR:ERVK:BGLII_Mus	624	0.004	0.004	0.007	0.003	1.688	0.707
LINE:L1:L1MCb	729	0.002	0.002	0.004	0.002	1.685	1.362
LTR:MaLR:MT-int	431	0.016	0.017	0.026	0.023	1.664	1.338
LINE:L1:L1VL4	6954	0.005	0.005	0.008	0.009	1.661	1.716
LTR:ERV1:LTRIS2	1439	0.086	0.084	0.142	0.277	1.656	3.302
LTR:ERVK:IAPLTR1_Mm	1511	0.056	0.052	0.093	0.132	1.654	2.552
LTR:ERVL:LTR40a	720	0.003	0.004	0.006	0.005	1.645	1.213
LTR:ERVK:RMER13B	2196	0.004	0.004	0.007	0.007	1.645	1.900
LTR:ERVK:RLTR44-int	635	0.024	0.024	0.040	0.005	1.644	0.201
LINE:L1:Lx3C	8467	0.002	0.002	0.004	0.003	1.643	1.128
LTR:ERVK:RLTR10C	1788	0.012	0.013	0.019	0.010	1.639	0.768
LINE:L1:L1M3d	162	0.006	0.010	0.010	0.004	1.634	0.364
LINE:L1:L1MA5	4975	0.007	0.007	0.012	0.006	1.630	0.872
LTR:ERVK:RLTR22_Mus	1258	0.013	0.013	0.020	0.018	1.614	1.417
LTR:ERVK:RMER16-int	3621	0.020	0.020	0.032	0.040	1.613	1.950
LTR:MaLR:MLT1D-int	176	0.010	0.012	0.015	0.004	1.610	0.310
LTR:ERVL:LTR84a	138	0.006	0.004	0.009	0.003	1.605	0.802
LTR:ERVL:HERVL74-int	151	0.013	0.016	0.021	0.015	1.604	0.960
LTR:ERV1:MER57F	181	0.007	0.007	0.011	0.018	1.604	2.573
LTR:ERV1:LTR37A	549	0.006	0.007	0.010	0.005	1.599	0.670
LTR:ERVK:RLTR43A	242	0.002	0.003	0.003	0.004	1.597	1.567
LTR:ERVK:IAP-d-int	1817	0.004	0.004	0.006	0.022	1.593	5.756
LTR:ERV1:LTRIS3	194	0.017	0.012	0.027	0.030	1.590	2.437
LINE:L1:L1Md_A	16293	0.002	0.002	0.004	0.005	1.590	2.273
LTR:ERV1:MER110	144	0.001	0.001	0.002	0.001	1.588	1.126
LTR:ERV1?:LTR81B	209	0.003	0.003	0.005	0.002	1.583	0.810
LTR:ERVK:RLTR25B	4500	0.016	0.019	0.025	0.044	1.582	2.336
LTR:ERVK:RLTR10B2	850	0.004	0.004	0.006	0.004	1.579	1.165

LTR:ERV1:MER89	618	0.032	0.032	0.050	0.104	1.576	3.261
LINE:L1:L1MEf	3524	0.009	0.010	0.015	0.006	1.575	0.603
LTR:ERV1:MuLV-int	474	0.302	0.334	0.473	0.534	1.569	1.602
LTR:Gypsy:MamGypLTR3	115	0.006	0.006	0.009	0.014	1.565	2.368
LINE:L1:L1MEg2	106	0.001	0.003	0.002	0.006	1.565	1.668
LTR:ERVK:RLTR9D	826	0.025	0.026	0.039	0.037	1.564	1.408
LTR:ERV1:MURVY-LTR	1489	0.005	0.005	0.007	0.006	1.563	1.332
LTR:ERV1:RMER2	2340	0.007	0.007	0.011	0.013	1.558	1.972
LTR:MaLR:MLT1E	533	0.013	0.015	0.020	0.008	1.558	0.534
LTR:ERV1:MURVY-int	434	0.000	0.000	0.000	0.001	1.554	3.780
LINE:L1:L1ME3	1250	0.006	0.005	0.009	0.004	1.554	0.871
LTR:ERV1:RodERV21-int	1848	0.006	0.006	0.009	0.015	1.552	2.435
LTR:ERV1:RLTR1C	707	0.006	0.006	0.009	0.009	1.551	1.589
LTR:ERVK:RMER6-int	613	0.004	0.005	0.006	0.006	1.550	1.081
LTR:MaLR:MLT1K	2798	0.040	0.043	0.063	0.059	1.550	1.393
LTR:ERV1:MER67A	175	0.002	0.003	0.003	0.003	1.549	0.979
LTR:ERVK:RLTR33	1583	0.016	0.016	0.025	0.021	1.548	1.302
LTR:MaLR:MLT1F1	1609	0.007	0.008	0.011	0.009	1.547	1.117
LTR:ERV1:MER31A	712	0.021	0.019	0.032	0.031	1.546	1.616
LINE:L1:L1_Mur2	19348	0.002	0.002	0.003	0.025	1.541	11.374
LTR:ERVK:RMER16	2041	0.009	0.010	0.015	0.024	1.537	2.374
LINE:L1:L1_Rod	11246	0.003	0.004	0.005	0.003	1.535	0.910
LTR:ERVK:RLTR13A3	243	0.009	0.008	0.014	0.012	1.534	1.499
LINE:L1:Lx3B	5831	0.006	0.006	0.009	0.006	1.534	1.050
LINE:L1:L1M2	18591	0.008	0.008	0.012	0.009	1.531	1.069
LTR:ERVK:RLTR45	1685	0.012	0.012	0.019	0.041	1.520	3.499
LTR:ERVK:RMER6D	2400	0.005	0.006	0.008	0.006	1.519	0.948
LTR:ERVK:RMER6C	4853	0.003	0.004	0.005	0.003	1.517	0.892
LTR:ERVL:LTR40b	574	0.003	0.005	0.005	0.004	1.517	0.918
LTR:MaLR:MLT1G1	1074	0.086	0.097	0.130	0.062	1.516	0.643
LTR:ERVK:MYSERV16_I-int	2514	0.007	0.007	0.010	0.004	1.512	0.567
LTR:MaLR:MLT1L	1554	0.006	0.005	0.009	0.062	1.502	12.219

LTR:ERVK:IAPLTR1a_Mm	1945	0.011	0.011	0.017	0.018	1.501	1.653
LTR:ERVK:RMER6B	2008	0.017	0.016	0.025	0.037	1.497	2.292
LTR:MaLR:MLT1H-int	366	0.002	0.003	0.003	0.002	1.495	0.705
LTR:ERV1:MMVL30-int	925	0.025	0.029	0.037	0.053	1.494	1.856
LTR:ERV1:MER21B	3495	0.016	0.017	0.024	0.026	1.490	1.554
LINE:L1:L1Md_F3	15614	0.003	0.003	0.004	0.006	1.489	1.871
LINE:L1:L1P5	177	0.017	0.016	0.026	0.010	1.486	0.608
LTR:ERVK:RLTR31_Mm	1178	0.008	0.008	0.012	0.008	1.482	1.003
LTR:ERVK:RLTR13D2	327	0.006	0.005	0.008	0.022	1.482	4.563
LTR:ERVK:RMER17A	1809	0.005	0.006	0.008	0.015	1.481	2.637
LTR:MaLR:MLT1G	872	0.004	0.003	0.005	0.007	1.472	2.248
LTR:ERVL:MER77	875	0.009	0.011	0.014	0.018	1.470	1.586
LTR:ERVK:RLTR13B3	263	0.010	0.010	0.015	0.020	1.467	2.120
LTR:ERVK:RLTR9E	1627	0.079	0.086	0.116	0.202	1.465	2.353
LINE:L1:L1_Mur1	10401	0.002	0.002	0.003	0.165	1.462	89.938
LINE:L1:Lx7	42027	0.003	0.004	0.005	0.004	1.462	1.185
LTR:ERVK:MYSERV6-int	3158	0.013	0.014	0.020	0.025	1.462	1.782
LTR:ERVL:LTR16A	1500	0.003	0.004	0.005	0.009	1.461	2.292
LTR:ERVK:RLTR31_Mur	648	0.015	0.014	0.021	0.010	1.460	0.747
LTR:MaLR:MLT1H2	1050	0.012	0.012	0.017	0.007	1.458	0.590
LINE:L1:L1MCa	3656	0.015	0.015	0.022	0.006	1.455	0.391
LTR:ERVK:RLTR20B2	952	0.005	0.005	0.007	0.003	1.454	0.685
LINE:L1:Lx3_Mus	12000	0.002	0.002	0.003	0.003	1.453	1.307
LINE:L1:L1M4c	3047	0.007	0.007	0.010	0.009	1.450	1.252
LTR:ERV1:MER34A	700	0.004	0.005	0.006	0.006	1.450	1.206
LTR:ERVK:RMER3D-int	2706	0.004	0.004	0.006	0.004	1.444	0.801
LTR:LTR:LTR90B	101	0.006	0.005	0.008	0.007	1.444	1.471
LTR:ERV1:RLTR1B-int	789	0.549	0.503	0.791	0.927	1.441	1.844
LINE:L1:L1_Mus4	11073	0.002	0.002	0.003	0.003	1.440	1.458
LTR:MaLR:MTEa-int	1396	0.007	0.008	0.011	0.008	1.440	1.034
LINE:L1:L1_Mus2	19548	0.002	0.003	0.004	0.011	1.440	4.244
LTR:ERVL:RLTR28B	841	0.010	0.009	0.014	0.013	1.437	1.477

LTR:ERVK:RLTR13A2	167	0.047	0.050	0.067	0.009	1.436	0.185
LINE:L1:L1MB2	2917	0.058	0.061	0.083	0.006	1.431	0.091
LINE:L1:L1Md_F2	65451	0.002	0.003	0.003	0.004	1.428	1.656
LTR:ERVK:RLTR13C2	930	0.009	0.011	0.013	0.011	1.427	0.998
LINE:L1:L1MA4A	2668	0.005	0.005	0.007	0.006	1.426	1.087
LTR:ERVK:RLTR20A1	277	0.012	0.014	0.018	0.006	1.425	0.392
LTR:ERVK:RMER20B	3475	0.011	0.012	0.016	0.020	1.422	1.608
LTR:ERVK:RMER17C	8430	0.011	0.011	0.016	0.025	1.420	2.244
LTR:ERV1:MuRRS4-int	2895	0.006	0.006	0.008	0.010	1.418	1.601
LTR:ERV1:MER92B	240	0.003	0.002	0.004	0.166	1.417	93.580
LTR:ERVK:RLTR9A2	241	0.011	0.014	0.016	0.019	1.415	1.382
LINE:L1:L1MA7	3248	0.008	0.008	0.011	0.005	1.414	0.698
LINE:L1:L1ME3B	1505	0.042	0.042	0.059	0.014	1.412	0.330
LINE:L1:L1ME4a	1778	0.062	0.063	0.087	0.019	1.409	0.304
LINE:L1:L1MD	6285	0.009	0.009	0.012	0.007	1.408	0.868
LTR:ERVK:RLTR45-int	2294	0.014	0.013	0.020	0.027	1.407	2.031
LTR:ERV1:LTR65	165	0.053	0.061	0.074	0.070	1.404	1.144
LTR:ERV1:MER34A1	540	0.256	0.240	0.359	0.372	1.402	1.548
LTR:ERVL:MERVL_2A-int	3762	0.017	0.018	0.024	0.067	1.402	3.773
LTR:ERVL:MT2C_Mm	2010	0.018	0.020	0.026	0.030	1.399	1.533
LTR:ERVK:RLTR13B1	1042	0.005	0.005	0.007	0.007	1.396	1.330
LTR:ERVK:RLTR44B	247	0.005	0.006	0.007	0.008	1.394	1.444
LTR:ERVK:RLTR9B	256	0.011	0.012	0.015	0.015	1.392	1.184
LTR:ERVK:MurERV4-int	1519	0.030	0.031	0.041	0.010	1.389	0.329
LINE:L1:L1MB4	2718	0.012	0.014	0.017	0.005	1.388	0.401
LINE:L1:L1MA6	7031	0.005	0.005	0.006	0.017	1.384	3.718
LTR:ERV1:RLTR23	3026	0.009	0.009	0.012	0.013	1.376	1.374
LTR:MaLR:MLT1A	5033	0.006	0.006	0.008	0.006	1.374	1.021
LINE:L1:L1MB1	2480	0.004	0.005	0.006	0.004	1.373	0.842
LINE:CR1:CR1_Mam	267	0.013	0.015	0.018	0.106	1.371	7.133
LINE:L1:Lx4A	10668	0.003	0.003	0.004	0.002	1.369	0.788
LTR:ERV1:MuRRS-int	811	0.003	0.003	0.004	0.007	1.367	2.518

LTR:ERVL:MLT2F	569	0.008	0.008	0.012	0.186	1.366	23.395
LTR:ERVK:RLTR8	310	0.012	0.015	0.017	0.048	1.361	3.139
LTR:ERVK:RLTR21	3539	0.009	0.009	0.012	0.011	1.359	1.202
LINE:L1:L1M5	17544	0.008	0.009	0.011	0.009	1.356	1.001
LTR:ERVK:RNERVK23-int	754	0.016	0.017	0.022	0.033	1.354	1.943
LTR:MaLR:ORR1A2-int	2093	0.011	0.011	0.015	0.025	1.353	2.215
LTR:ERVL:LTR79	380	0.009	0.009	0.012	0.009	1.348	1.007
LINE:L1:L1MB5	3769	0.013	0.013	0.017	0.013	1.347	0.944
LINE:L1:L1MEg	4286	0.044	0.041	0.059	0.072	1.347	1.754
LTR:ERVL:LTR33A	505	0.004	0.004	0.005	0.031	1.346	7.174
LINE:L1:L1ME2	2660	0.007	0.007	0.010	0.006	1.345	0.846
LTR:ERVK:RLTR44C	202	0.106	0.104	0.142	0.005	1.345	0.047
LTR:ERVK:MERVK26-int	1010	0.004	0.004	0.005	0.022	1.344	5.741
LINE:L1:Lx6	29266	0.004	0.004	0.005	0.003	1.342	0.859
LINE:L1:L1_Mur3	27264	0.004	0.004	0.005	0.004	1.337	0.844
LTR:ERVK:RLTR10E	688	0.063	0.070	0.084	0.025	1.337	0.355
LTR:ERVL:MLT2B4	1871	0.004	0.004	0.006	0.006	1.336	1.564
LINE:L1:Lx5	28509	0.005	0.005	0.006	0.033	1.336	6.881
LTR:ERVL:MT2B2	3958	0.035	0.038	0.047	0.108	1.335	2.852
LINE:L1:L1MA4	7484	0.010	0.010	0.013	0.004	1.334	0.406
LINE:L1:Lx3A	8441	0.002	0.002	0.003	0.003	1.334	1.412
LINE:L1:L1MDb	573	0.029	0.029	0.039	0.006	1.331	0.216
LTR:ERVK:IAPEY3_LTR	983	0.007	0.006	0.009	0.004	1.331	0.666
LINE:L1:L1MDa	3489	0.008	0.008	0.010	0.005	1.328	0.603
LINE:L1:L1MB3	5357	0.015	0.016	0.020	0.011	1.327	0.647
LINE:L1:L1M7	261	0.008	0.007	0.010	0.016	1.326	2.351
LTR:Gypsy?:LTR88b	180	0.009	0.006	0.011	0.015	1.323	2.286
LTR:ERVL:MLT2B2	1340	0.006	0.007	0.007	0.003	1.323	0.423
LTR:ERV1:RMER5	7415	0.009	0.008	0.012	0.015	1.322	1.737
LINE:L1:Lx9	56621	0.005	0.005	0.006	0.004	1.321	0.825
LTR:ERVL:RMER15	17921	0.014	0.015	0.019	0.037	1.319	2.430
LTR:ERVL?:LTR89	141	0.027	0.029	0.035	0.032	1.319	1.120

LTR:ERVK:RLTR18B	2369	0.009	0.010	0.011	0.024	1.319	2.372
LTR:ERV1:RLTR6-int	1414	0.010	0.011	0.013	0.027	1.317	2.476
SINE:Alu:B1_Mur2	31802	0.069	0.071	0.090	0.090	1.317	1.274
LTR:ERV1:RLTR14	2809	0.009	0.009	0.011	0.008	1.316	0.980
LTR:ERVK:IAPLTR2b	1086	0.006	0.007	0.008	0.008	1.316	1.248
LTR:Gypsy:MamGypLTR2c	115	0.002	0.002	0.002	0.009	1.314	4.616
LINE:L1:Lx2A1	2716	0.002	0.002	0.002	0.002	1.313	0.918
LTR:ERVK:RMER17D2	1734	0.003	0.003	0.004	0.007	1.312	2.187
LTR:ERVK:RLTR13B4	380	0.003	0.003	0.004	0.005	1.311	1.738
SINE:B2:B2_Mm2	89635	0.043	0.055	0.056	0.082	1.310	1.473
LINE:L1:L1MC3	5330	0.007	0.007	0.010	0.004	1.303	0.597
LTR:ERVK:RMER12B	1038	0.011	0.013	0.015	0.009	1.302	0.660
LTR:ERVK:RLTR40	2658	0.008	0.008	0.010	0.008	1.302	0.946
LTR:ERVL:RLTR28	2016	0.014	0.014	0.018	0.019	1.301	1.358
LINE:L1:Lx4B	11010	0.005	0.005	0.007	0.002	1.299	0.367
LINE:L1:L1VL2	4520	0.006	0.006	0.007	0.009	1.296	1.610
LTR:MaLR:ORR1E	28160	0.009	0.010	0.012	0.012	1.295	1.304
LTR:ERVL:RMER10B	3328	0.020	0.020	0.026	0.028	1.293	1.377
LTR:ERVL:MLT2B3	1502	0.004	0.004	0.005	0.004	1.293	0.971
LTR:ERVK:RLTR12B	2939	0.068	0.068	0.088	0.057	1.292	0.834
LTR:ERVK:IAPEY3-int	2324	0.004	0.004	0.005	0.007	1.291	1.899
LINE:L2:L2c	11803	0.051	0.056	0.066	0.105	1.291	1.867
LTR:ERVK:RLTR13D3	801	0.004	0.004	0.005	0.004	1.290	1.176
LINE:L1:L1_Mm	15845	0.003	0.004	0.004	0.006	1.286	1.593
LINE:L1:L1M3	7083	0.008	0.008	0.010	0.006	1.285	0.765
LINE:L1:Lx	24366	0.004	0.004	0.005	0.005	1.284	1.216
LTR:MaLR:ORR1B1-int	2996	0.025	0.025	0.032	0.036	1.282	1.413
SINE:B4:RSINE1	115483	0.037	0.038	0.047	0.060	1.282	1.585
LTR:ERVK:RLTR20D	1449	0.019	0.018	0.025	0.005	1.280	0.302
LTR:MaLR:ORR1E-int	1588	0.007	0.006	0.009	0.008	1.279	1.179
LINE:L1:L1MD1	2300	0.004	0.003	0.005	0.003	1.279	0.883
LTR:ERVK:RMER17A-int	408	0.004	0.005	0.006	0.022	1.277	4.467

LINE:L1:L1MC5	2621	0.016	0.016	0.020	0.015	1.277	0.916
LINE:L1:Lx2	18182	0.004	0.004	0.005	0.004	1.273	0.925
LTR:ERVK:RLTR20A2	888	0.012	0.013	0.015	0.004	1.273	0.315
LINE:L1:L1MD2	3224	0.027	0.029	0.035	0.016	1.272	0.562
LINE:L1:Lx8	65407	0.007	0.007	0.008	0.006	1.267	0.882
LINE:L1:L1PB4	143	0.004	0.003	0.004	0.001	1.266	0.263
LINE:L1:L1MB8	6004	0.013	0.013	0.017	0.007	1.265	0.552
LTR:ERVL:MLT2E	126	0.006	0.006	0.007	0.009	1.265	1.618
LINE:L1:L1ME2z	1528	0.010	0.011	0.013	0.005	1.264	0.438
SINE:Alu:B1_Mus1	93733	0.057	0.060	0.072	0.083	1.263	1.378
LINE:L1:HAL1b	508	0.015	0.016	0.019	0.010	1.263	0.601
LINE:L1:L1MEc	5386	0.005	0.006	0.007	0.004	1.261	0.674
SINE:B2:B3A	92388	0.031	0.033	0.039	0.049	1.261	1.504
LTR:ERVL:ERVL-B4-int	1421	0.005	0.005	0.006	0.026	1.260	5.646
LTR:ERV1:MER65D	106	0.008	0.007	0.010	0.006	1.259	0.876
LINE:L1:L1MC4	6020	0.013	0.013	0.017	0.008	1.258	0.631
LTR:MaLR:MLT1A0	10496	0.007	0.007	0.009	0.008	1.255	1.132
SINE:ID:ID	7858	0.044	0.045	0.056	0.050	1.255	1.127
LTR:ERVL:MER54B	116	0.020	0.025	0.025	0.041	1.255	1.605
LINE:L1:L1MB7	6940	0.028	0.029	0.035	0.008	1.250	0.263
SINE:Alu:B1F1	20755	0.095	0.100	0.118	0.094	1.247	0.935
LTR:ERVK:SRV_MM-int	243	0.107	0.105	0.133	0.144	1.243	1.374
LINE:L1:L1M4	12388	0.015	0.016	0.018	0.011	1.243	0.725
LINE:L1:L1MC1	5619	0.006	0.007	0.008	0.005	1.241	0.803
SINE:Alu:B1_Mus2	70597	0.069	0.073	0.086	0.111	1.240	1.517
LINE:L1:HAL1-3A_ME	6749	0.058	0.064	0.071	0.079	1.238	1.244
LTR:ERVK:RMER13A	2926	0.007	0.007	0.009	0.013	1.236	1.882
LINE:L1:L1Md_F	5937	0.002	0.003	0.003	0.010	1.236	4.023
LINE:L2:L2a	23161	0.036	0.037	0.044	0.049	1.236	1.344
LTR:MaLR:MTE2a	15455	0.020	0.021	0.025	0.028	1.233	1.289
LTR:MaLR:MTE2b	14490	0.011	0.012	0.013	0.011	1.231	0.989
SINE:Alu:B1_Mur4	40539	0.054	0.057	0.066	0.063	1.231	1.104

LTR:ERV1:MER21-int	110	0.012	0.013	0.015	0.004	1.231	0.313
LTR:ERVK:RLTR10B	478	0.009	0.009	0.011	0.017	1.228	1.865
LTR:MaLR:ORR1D2	15360	0.010	0.010	0.012	0.011	1.228	1.046
LINE:L1:L1_Mus1	27653	0.006	0.006	0.007	0.025	1.228	4.190
LTR:ERVK:RLTR11A	3041	0.018	0.018	0.022	0.017	1.227	0.946
LTR:ERVK:RMER6A	3987	0.023	0.025	0.028	0.021	1.224	0.839
LTR:ERVK:RLTR10	2929	0.009	0.009	0.011	0.007	1.224	0.792
LTR:ERVK:RMER19A	2200	0.009	0.009	0.011	0.012	1.222	1.366
LTR:MaLR:ORR1B2-int	865	0.006	0.006	0.007	0.004	1.219	0.713
LTR:ERVK:IAPLTR3	664	0.003	0.002	0.004	0.002	1.217	0.904
SINE:Alu:B1_Mm	42397	0.062	0.064	0.075	0.117	1.214	1.827
LINE:L1:L1ME3A	2329	0.008	0.008	0.009	0.006	1.213	0.688
LTR:MaLR:MTC-int	3550	0.016	0.015	0.019	0.014	1.213	0.909
LINE:L1:L1M4b	2759	0.013	0.014	0.016	0.009	1.212	0.662
LINE:L1:L1_Mus3	24126	0.005	0.004	0.006	0.006	1.211	1.442
LINE:L1:L1ME5	145	0.021	0.023	0.026	0.002	1.208	0.091
LTR:ERVK:RLTR11B	1983	0.030	0.029	0.036	0.029	1.208	1.015
LTR:MaLR:ORR1C1-int	783	0.031	0.030	0.038	0.036	1.207	1.213
LTR:MaLR:MTE-int	5136	0.013	0.013	0.015	0.015	1.205	1.125
LTR:ERVL:HERV16-int	438	0.009	0.008	0.011	0.002	1.205	0.217
LTR:ERV1:RLTR41	1911	0.012	0.012	0.014	0.026	1.204	2.119
LTR:ERVK:RLTR15	5513	0.032	0.034	0.039	0.029	1.203	0.845
LTR:ERV1:LTR48	648	0.005	0.005	0.007	0.011	1.201	1.978
LINE:L1:L1MC2	2203	0.017	0.019	0.021	0.004	1.201	0.218
SINE:Alu:B1F2	16782	0.078	0.082	0.094	0.057	1.199	0.692
LTR:MaLR:MTD-int	2482	0.013	0.014	0.015	0.012	1.199	0.827
SINE:B2:B3	147277	0.036	0.039	0.043	0.044	1.199	1.143
LTR:ERVL:LTR16E2	428	0.002	0.002	0.002	0.002	1.198	0.888
LTR:ERVK:RMER4A	3106	0.021	0.022	0.025	0.016	1.197	0.739
LTR:ERVK:RLTR25A	3222	0.010	0.010	0.012	0.029	1.194	2.856
SINE:Alu:PB1D7	24985	0.084	0.089	0.100	0.091	1.194	1.024
LTR:MaLR:MLT1B-int	164	0.022	0.020	0.026	0.003	1.194	0.135

LTR:MaLR:ORR1A1-int	856	0.015	0.015	0.018	0.028	1.193	1.879
SINE:ID:ID4_	26405	0.078	0.078	0.093	0.073	1.191	0.942
LTR:MaLR:MLT1N2	727	0.007	0.007	0.009	0.010	1.190	1.421
LTR:ERVK:MYSERV-int	1447	0.009	0.010	0.011	0.012	1.190	1.212
SINE:Alu:B1_Mur1	37980	0.062	0.067	0.074	0.068	1.189	1.025
LTR:ERVK:RLTR12A	971	0.008	0.008	0.009	0.005	1.188	0.580
SINE:B4:ID_B1	111246	0.061	0.064	0.073	0.068	1.188	1.057
LTR:MaLR:ORR1A4-int	1316	0.067	0.069	0.079	0.090	1.187	1.308
LTR:ERVL:MLT2B1	1656	0.004	0.004	0.004	0.004	1.185	1.170
LINE:L1:L1VL1	2852	0.004	0.004	0.004	0.008	1.184	2.059
LTR:ERVK:RLTR20A	1090	0.013	0.012	0.015	0.006	1.184	0.513
LTR:ERVL:LTR83	161	0.096	0.092	0.114	0.002	1.183	0.021
SINE:Alu:B1_Mur3	24658	0.072	0.076	0.085	0.091	1.183	1.197
LTR:ERVK:RLTR11A2	3350	0.012	0.012	0.015	0.014	1.182	1.137
LTR:MaLR:MTB-int	591	0.287	0.275	0.339	0.018	1.180	0.065
LTR:MaLR:ORR1C2	11826	0.018	0.019	0.021	0.016	1.179	0.823
LTR:ERVL:LTR41	512	0.009	0.010	0.010	0.015	1.176	1.455
LINE:L1:L1MA9	4608	0.004	0.004	0.005	0.004	1.175	0.957
LTR:ERV1:MER57E1	166	0.004	0.004	0.004	0.001	1.175	0.354
LTR:ERVK:ETnERV-int	404	0.044	0.049	0.052	0.120	1.173	2.475
LINE:L1:L1MEd	1051	0.011	0.011	0.013	0.003	1.168	0.316
LTR:ERVK:RLTR22_Mur	3625	0.010	0.010	0.011	0.008	1.167	0.815
LTR:MaLR:MTC	26761	0.013	0.014	0.016	0.015	1.166	1.086
LTR:MaLR:MTEb	12207	0.009	0.010	0.011	0.017	1.164	1.805
LTR:ERVL:LTR80A	110	0.002	0.001	0.003	0.001	1.163	1.066
LTR:ERV1:MER90	250	0.004	0.005	0.004	0.007	1.161	1.377
LTR:ERVL:LTR16B	273	0.014	0.011	0.016	0.012	1.160	1.129
LTR:ERVL:LTR33	1459	0.005	0.006	0.006	0.005	1.159	0.874
LTR:MaLR:MLT1J1	1131	0.006	0.007	0.006	0.006	1.157	0.884
LTR:ERVL:LTR41B	286	0.012	0.016	0.014	0.084	1.157	5.364
LTR:MaLR:ORR1C2-int	908	0.018	0.018	0.021	0.017	1.157	0.918
SINE:Alu:PB1D10	72983	0.072	0.075	0.083	0.084	1.154	1.117

SINE:B4:B4	63984	0.021	0.021	0.024	0.030	1.154	1.408
LTR:ERV1:LTRIS5	268	0.015	0.014	0.017	0.015	1.153	1.070
LTR:ERVK:RLTR16	2360	0.014	0.015	0.016	0.008	1.153	0.544
LTR:ERVL:MT2B	16606	0.038	0.040	0.043	0.051	1.150	1.261
LTR:ERVL:MER68	818	0.033	0.034	0.038	0.065	1.150	1.925
LTR:MaLR:ORR1A2	14647	0.016	0.016	0.018	0.025	1.149	1.526
LTR:ERVK:RLTR17	2701	0.016	0.015	0.019	0.023	1.149	1.536
LTR:ERVK:IAPLTR2a	983	0.015	0.014	0.017	0.011	1.140	0.749
SINE:Alu:PB1D9	29287	0.120	0.125	0.137	0.159	1.140	1.273
LTR:ERVK:RMER17C-int	1926	0.009	0.009	0.010	0.013	1.136	1.440
LINE:L2:L2b	12890	0.045	0.046	0.052	0.053	1.135	1.166
LTR:ERVK:RMER17A2	2297	0.024	0.026	0.028	0.026	1.132	1.009
LTR:ERVK:RMER12	5715	0.012	0.013	0.014	0.010	1.132	0.796
LTR:ERV1:MER31-int	231	0.017	0.015	0.020	0.015	1.131	1.003
LTR:ERVK:BGLII_B	1775	0.015	0.015	0.017	0.008	1.130	0.529
LINE:RTE:L5	122	0.002	0.003	0.002	0.005	1.129	1.609
SINE:Alu:PB1	13667	0.072	0.076	0.082	0.085	1.129	1.117
SINE:MIR:MIR3	9048	0.037	0.036	0.042	0.055	1.128	1.522
SINE:MIR:MIRb	39832	0.052	0.056	0.059	0.069	1.125	1.222
LTR:ERVK:RLTR10-int	2708	0.010	0.009	0.011	0.010	1.125	1.067
LTR:ERVL:RMER10A	4456	0.020	0.019	0.022	0.052	1.125	2.679
LTR:MaLR:ORR1A4	7769	0.037	0.038	0.041	0.042	1.124	1.099
LTR:MaLR:MLT1E2	1833	0.009	0.011	0.011	0.008	1.124	0.720
LINE:L1:L1MA5A	1241	0.009	0.008	0.010	0.007	1.122	0.839
LTR:MaLR:ORR1B1	16289	0.020	0.021	0.023	0.021	1.122	1.014
LTR:MaLR:ORR1D1	21222	0.017	0.016	0.019	0.013	1.121	0.803
LTR:ERV1:LTRIS_Mm	567	0.015	0.014	0.017	0.022	1.119	1.628
LTR:MaLR:MLT1B	9348	0.015	0.015	0.016	0.012	1.118	0.779
LTR:ERVL:LTR40c	245	0.010	0.010	0.012	0.004	1.116	0.396
LINE:L1:L1MA10	886	0.003	0.003	0.003	0.003	1.116	1.122
LTR:ERVL:LTR82A	339	0.006	0.008	0.007	0.005	1.114	0.601
LTR:ERVK:RMER19B	5926	0.022	0.023	0.024	0.020	1.110	0.895

LTR:MaLR:MLT1I	1793	0.004	0.004	0.005	0.006	1.109	1.814
LINE:L1:L1ME1	5235	0.027	0.028	0.030	0.010	1.108	0.344
LTR:ERVK:RLTR27	1637	0.006	0.006	0.006	0.011	1.106	2.003
LTR:ERV1:RLTR24	1442	0.013	0.014	0.014	0.014	1.104	1.038
LTR:Gypsy?:LTR85b	109	0.004	0.002	0.004	0.007	1.101	3.823
LINE:L1:L1M3b	164	0.001	0.001	0.001	0.000	1.099	0.402
LTR:ERVK:RLTR46	592	0.026	0.027	0.028	0.023	1.095	0.833
LTR:ERVK:RMER19C	4682	0.008	0.008	0.009	0.008	1.093	1.005
SINE:Alu:B1F	45775	0.063	0.065	0.069	0.073	1.092	1.108
LTR:MaLR:MLT1H	2326	0.009	0.009	0.009	0.005	1.092	0.540
LINE:RTE:L4	4028	0.145	0.139	0.158	0.227	1.091	1.633
LTR:ERV1:MER57D	300	0.002	0.002	0.002	0.003	1.090	1.977
LINE:L1:L1MC	6484	0.009	0.009	0.010	0.006	1.089	0.679
LTR:MaLR:MLT1A0-int	208	0.006	0.005	0.006	0.014	1.089	2.946
SINE:B4:B4A	106408	0.025	0.026	0.027	0.030	1.088	1.161
LTR:MaLR:MLT1F2	2292	0.011	0.011	0.012	0.008	1.085	0.712
LTR:MaLR:MTD	50109	0.014	0.015	0.016	0.014	1.084	0.894
LTR:ERVK:RLTR42	264	0.023	0.025	0.025	0.035	1.084	1.391
LTR:ERVL:LTR16C	1365	0.003	0.004	0.004	0.014	1.083	3.996
LTR:ERVK:IAPLTR3-int	1395	0.002	0.002	0.002	0.002	1.083	0.992
LTR:ERVK:RMER17B	3780	0.006	0.006	0.007	0.007	1.082	1.191
LTR:MaLR:ORR1A3	4389	0.016	0.016	0.017	0.028	1.080	1.738
LTR:MaLR:ORR1D1-int	1734	0.030	0.028	0.033	0.006	1.079	0.213
LINE:L2:L2	17627	0.030	0.030	0.033	0.034	1.079	1.127
LTR:MaLR:MLT1A1-int	138	0.011	0.014	0.012	0.007	1.077	0.455
LTR:ERV1:MER67B	224	0.007	0.009	0.008	0.006	1.076	0.700
LINE:L1:L1MC4a	3655	0.030	0.030	0.032	0.014	1.075	0.475
LTR:MaLR:MTA_Mm-int	3024	0.039	0.043	0.042	0.058	1.074	1.335
LTR:ERV1:LTRIS4	323	0.037	0.034	0.039	0.043	1.071	1.261
LTR:ERVK:ETnERV2-int	5884	0.026	0.027	0.027	0.065	1.069	2.415
LTR:ERVK:RLTR10A	1205	0.010	0.007	0.010	0.013	1.069	1.724
LTR:MaLR:MLT1A1	9356	0.008	0.009	0.009	0.006	1.069	0.649

SINE:B2:B2_Mm1t	23118	0.208	0.324	0.222	0.416	1.069	1.283
LINE:L1:L1MD3	2484	0.008	0.008	0.009	0.006	1.068	0.790
LTR:ERVK:RLTR19C	425	0.013	0.013	0.014	0.009	1.066	0.673
LTR:MaLR:MTA_Mm	15531	0.020	0.021	0.021	0.024	1.065	1.127
LTR:MaLR:MLT1J	2938	0.010	0.009	0.011	0.008	1.065	0.907
LTR:MaLR:ORR1C1	7464	0.019	0.019	0.020	0.014	1.064	0.712
LTR:MaLR:ORR1A0	2140	0.035	0.036	0.037	0.098	1.064	2.745
LTR:MaLR:MTEa	21182	0.011	0.011	0.012	0.012	1.060	1.097
SINE:MIR:MIR	42945	0.026	0.027	0.027	0.034	1.055	1.281
LTR:MaLR:ORR1B2	8305	0.016	0.017	0.017	0.019	1.054	1.152
LTR:MaLR:ORR1A1	4255	0.026	0.029	0.028	0.028	1.051	0.972
LTR:MaLR:MLT1G3	1067	0.012	0.011	0.013	0.029	1.050	2.525
LTR:ERV1:LTR73	155	0.008	0.008	0.008	0.013	1.050	1.642
LTR:ERVL:MLT2B5	446	0.191	0.208	0.200	0.203	1.048	0.977
LTR:ERV1?:LTR81A	177	0.003	0.003	0.003	0.004	1.044	1.335
LINE:L1:L1MEa	167	0.004	0.004	0.004	0.009	1.044	2.340
LTR:ERVK:IAPEY2_LTR	1063	0.003	0.003	0.003	0.007	1.039	2.658
LTR:ERVK:RMER4B	5082	0.046	0.048	0.048	0.028	1.038	0.589
LTR:MaLR:MTB	6393	0.049	0.047	0.050	0.017	1.037	0.362
LTR:MaLR:MLT1-int	271	0.003	0.004	0.003	0.008	1.034	2.096
LTR:ERV1:RLTR1	306	0.105	0.103	0.108	0.118	1.034	1.140
LTR:MaLR:MLT1D	8382	0.017	0.017	0.017	0.018	1.033	1.041
LTR:ERVL?:LTR87	211	0.037	0.033	0.038	0.017	1.032	0.504
LINE:L1:L1M2a	211	0.013	0.012	0.013	0.003	1.031	0.223
LTR:ERVK:RLTR9F	404	0.004	0.003	0.004	0.004	1.030	1.529
SINE:MIR:MIRm	8661	0.042	0.042	0.044	0.059	1.029	1.413
LTR:ERV1:RLTR30	389	0.017	0.014	0.018	0.023	1.028	1.720
LTR:ERVL:LTR16A1	778	0.001	0.001	0.001	0.003	1.027	2.600
SINE:MIR:MIRc	21284	0.089	0.090	0.091	0.084	1.027	0.936
LTR:ERVK:RLTR18	1844	0.064	0.066	0.066	0.165	1.025	2.523
LINE:L1:L1M3c	338	0.003	0.004	0.003	0.003	1.023	0.855
LTR:ERVK:RLTR9C	792	0.031	0.031	0.032	0.043	1.022	1.390

SINE:ID:ID4	23957	0.091	0.094	0.093	0.082	1.021	0.872
LTR:ERV1:LTR37B	380	0.008	0.006	0.008	0.001	1.020	0.234
LTR:ERVK:IAPLTR4	562	0.009	0.009	0.009	0.011	1.019	1.284
LTR:ERVK:RLTR20B1	807	0.023	0.025	0.023	0.016	1.019	0.636
LTR:Gypsy:MamGypLTR2b	111	0.006	0.004	0.006	0.007	1.016	1.853
LTR:ERVK:MMETn-int	1965	0.406	0.474	0.413	0.761	1.016	1.606
Low_complexity:ERVL:LTR82B	320	0.002	0.002	0.002	0.005	1.016	2.165
LTR:ERVL:MT2A	14256	0.024	0.026	0.025	0.015	1.015	0.560
LTR:MaLR:MLT-int	849	0.036	0.034	0.037	0.014	1.012	0.417
LTR:ERVK:RLTR13D1	755	0.010	0.010	0.010	0.009	1.011	0.901
LTR:ERVL:ERVL-int	745	0.003	0.002	0.003	0.003	1.007	1.493
SINE:B2:B2_Mm1a	18354	0.407	0.634	0.409	0.859	1.006	1.355
LTR:ERVL:RMER15-int	3115	0.015	0.016	0.015	0.011	1.003	0.721
LINE:L1:L1MA8	3839	0.020	0.021	0.020	0.011	0.999	0.506
SINE:ID:ID2	5637	0.115	0.118	0.115	0.087	0.998	0.742
LTR:ERV1:MER31B	552	0.013	0.015	0.013	0.010	0.990	0.645
LTR:ERVK:RLTR31B_Mm	417	0.017	0.018	0.017	0.005	0.989	0.249
LTR:ERVK:BGLII	2271	0.014	0.012	0.014	0.018	0.986	1.410
LTR:ERV1:MER90a	569	0.010	0.011	0.009	0.005	0.981	0.483
LTR:ERVK:RLTR31A_Mm	1012	0.013	0.011	0.012	0.013	0.981	1.203
LTR:MaLR:MTE2a-int	684	0.007	0.006	0.007	0.035	0.979	5.971
SINE:Alu:FAM	215	0.162	0.176	0.158	0.099	0.975	0.564
LTR:ERVK:IAPLTR2_Mm	2285	0.014	0.014	0.013	0.012	0.975	0.839
LINE:L1:HAL1	3825	0.024	0.022	0.023	0.112	0.973	4.970
LTR:ERVL:MLT2C2	653	0.006	0.005	0.006	0.007	0.972	1.217
LTR:ERV:LTR55	150	0.007	0.005	0.007	0.007	0.971	1.343
LINE:CR1:L3	9768	0.102	0.107	0.099	0.100	0.969	0.937
LTR:MaLR:MLT1J2	1102	0.008	0.010	0.008	0.076	0.969	7.754
LTR:MaLR:MTB_Mm	4860	0.016	0.016	0.016	0.011	0.967	0.698
LTR:ERVK:RLTR19A	157	0.091	0.122	0.088	0.029	0.967	0.241
LTR:MaLR:MLT1C	7782	0.023	0.022	0.022	0.006	0.963	0.288
LTR:ERVK:RLTRETN_Mm	2864	0.108	0.115	0.104	0.194	0.962	1.684

SINE:SINE:LFSINE_Vert	344	0.094	0.100	0.090	0.004	0.958	0.039
LTR:ERVL:LTR16D	178	0.021	0.020	0.020	0.013	0.956	0.627
LINE:L1:Lx2B	12773	0.006	0.006	0.006	0.009	0.955	1.440
LTR:ERVK:RLTR9A	1791	0.016	0.017	0.015	0.020	0.953	1.172
LINE:L1:L1M2c	315	0.004	0.004	0.004	0.013	0.947	3.609
LTR:MaLR:ORR1D-int	796	0.013	0.014	0.012	0.018	0.937	1.235
SINE:SINE:AmnSINE1	518	0.023	0.025	0.021	0.011	0.934	0.432
LTR:ERV1:LTRIS_Mus	879	0.042	0.043	0.039	0.092	0.931	2.170
LTR:Gypsy:MamGypLTR1a	140	0.002	0.001	0.002	0.002	0.931	1.540
LTR:MaLR:MTB_Mm-int	377	0.023	0.027	0.021	0.006	0.928	0.208
LTR:ERV1:RLTR1B	1559	0.140	0.135	0.130	0.209	0.924	1.553
LTR:ERVL:LTR16B2	384	0.004	0.004	0.003	0.003	0.923	0.944
LTR:ERVL:LTR50	549	0.250	0.250	0.231	0.158	0.923	0.632
LTR:ERVL:LTR53	273	0.004	0.003	0.004	0.004	0.914	1.560
LTR:ERV1:MER57C2	409	0.008	0.008	0.007	0.007	0.911	0.873
LTR:ERVK:RLTR13C1	661	0.008	0.009	0.007	0.012	0.910	1.268
LTR:MaLR:MLT1M	248	0.374	0.377	0.340	0.146	0.910	0.387
LTR:ERVK:MMTV-int	158	0.021	0.024	0.019	0.014	0.901	0.585
SINE:Alu:AluF_3	166	0.049	0.029	0.043	0.392	0.891	13.487
LTR:ERVK:RLTR19-int	2543	0.273	0.279	0.243	0.802	0.889	2.878
LTR:ERVK:RLTR18-int	506	0.032	0.033	0.028	0.070	0.887	2.154
LINE:L1:L1MCc	513	0.010	0.010	0.009	0.004	0.884	0.404
LTR:Gypsy:MamGypLTR1c	148	0.004	0.003	0.003	0.001	0.881	0.246
LTR:ERVK:RLTR20B3	2403	0.015	0.014	0.013	0.006	0.879	0.436
LTR:ERV1:MER34	750	0.009	0.008	0.008	0.009	0.875	1.156
LTR:ERV1:LTR78	588	0.018	0.015	0.015	0.005	0.875	0.309
LTR:MaLR:MLT1F-int	417	0.022	0.018	0.019	0.004	0.873	0.212
LTR:ERVK:RLTR20C	1408	0.027	0.027	0.024	0.019	0.872	0.701
LTR:ERVL:LTR33B	353	0.003	0.003	0.002	0.003	0.871	1.339
LTR:MaLR:ORR1A0-int	453	0.050	0.054	0.043	0.136	0.868	2.509
LTR:ERVL:LTR16E1	513	0.004	0.004	0.004	0.009	0.860	2.389
LTR:MaLR:MLT1H1	992	0.007	0.008	0.006	0.006	0.851	0.831

LINE:L1:L1M3e	641	0.019	0.020	0.016	0.006	0.847	0.299
LINE:CR1:L3b	1705	0.034	0.033	0.029	0.044	0.843	1.338
LTR:ERVL:MER76	240	0.032	0.031	0.027	0.016	0.842	0.533
LTR:MaLR:MLT1F	1550	0.010	0.009	0.008	0.007	0.840	0.789
LTR:ERVL:MER74A	731	0.007	0.005	0.006	0.005	0.840	1.064
LTR:ERVL:LTR33A_	475	0.016	0.015	0.013	0.009	0.823	0.588
LTR:ERVL:LTR33C	229	0.096	0.094	0.078	0.017	0.817	0.177
LTR:MaLR:MLT1E1A	1155	0.018	0.019	0.015	0.004	0.817	0.225
LTR:ERV1:RMER21A	632	0.096	0.097	0.078	0.069	0.815	0.715
LTR:ERVL:MER54A	276	0.008	0.007	0.006	0.014	0.814	2.115
LTR:ERV1:LTR48B	307	0.006	0.004	0.005	0.003	0.813	0.665
LTR:ERVK:BGLII_A	308	0.015	0.014	0.012	0.014	0.785	1.029
LTR:ERV1:MMERGLN-int	842	0.438	0.439	0.343	0.922	0.784	2.102
LTR:ERV1:RLTR5_Mm	439	0.006	0.006	0.005	0.005	0.747	0.911
LTR:ERV1:MER57A-int	231	0.013	0.013	0.010	0.006	0.741	0.432
LTR:ERV1?:LTR81C	111	0.166	0.158	0.119	0.216	0.716	1.367
LTR:ERVK:RLTR9B2	343	0.018	0.019	0.013	0.012	0.707	0.621
LTR:MaLR:MTEb-int	659	0.007	0.007	0.005	0.007	0.684	1.053
LTR:ERVL:HERVL40-int	206	0.130	0.130	0.089	0.093	0.681	0.715
LTR:ERV1:RLTR14-int	1827	0.019	0.019	0.013	0.014	0.668	0.727
LTR:Gypsy:MamGypLTR1d	111	0.001	0.001	0.000	0.013	0.663	12.079
LTR:ERVL:MER74C	117	0.002	0.001	0.002	0.002	0.660	1.475
LTR:ERVL:MER74B	326	0.005	0.004	0.003	0.009	0.634	2.234
LINE:L1:HAL1-2a_MD	1302	0.208	0.212	0.131	0.339	0.632	1.602
LTR:MaLR:MLT1A-int	120	0.003	0.001	0.002	0.001	0.606	0.765
LTR:ERVK:RLTR44E	121	0.019	0.019	0.011	0.020	0.564	1.072
LTR:ERV1:MER110A	192	0.006	0.007	0.004	0.001	0.551	0.160
LTR:MaLR:MLT1E3	1031	0.026	0.024	0.013	0.008	0.505	0.358
LINE:L1:L1M3a	124	0.003	0.000	0.001	0.000	0.448	0.490
LTR:ERVL:LTR52	230	0.001	0.001	0.000	0.001	0.354	0.989
LTR:ERVL:LTR16B1	381	0.013	0.011	0.004	0.006	0.304	0.571
