# Supplementary material for: hnRNP K Coordinates Transcriptional Silencing by SETDB1 in Embryonic Stem Cells
Source: PLoS Genet. 2015 Jan 22;11(1):e1004933. doi: 10.1371/journal.pgen.1004933 (PMC4303303; doi:10.1371/journal.pgen.1004933)
Supplement: S2 Table — (PDF) [file pgen.1004933.s010.pdf]

Genes upregulated in hnRNP K KD and Setdb1 KO (54)

Red = SETDB1-bound and/or marked by SETDB1-dependent H3K9me3 (30)

Cd97	Ddit4l
Casp14	Igf2
Taf7l	Wfdc15a
Dazl	Zscan4c
Gadd45b	Foxi3
Sdc4	Dub1a
Pltp	Tex101
Perp	Slc25a31
Mdm2	Zscan4f
Ccng1	Gm12794
Btg2	C4b
Id2	Acad11
Plk2	Zscan4d
Anxa8	
1190002H23Rik	
Cdkn1a	
Eps8l2	
Cd55	
Chit1	
1700007K13Rik	
Cyr61	
Trp53inp1	
Tinagl1	
Abcb1b	
Lrpap1	
Agpat9	
Slc25a4	
Gabarapl2	
Tbx20	
Glt25d2	
Wnt6	
Cml2	
Rtkn	
Gm12800	
Vnn1	
Ltb4r2	
Mael	
Dub3	
Ly6k	
Ankrd45	
8030474K03Rik	

Genes upregulated in common between hnRNP K KD and Kap1 KO (81)

Bgn
Tmem92-ps
Gm13078
BC080695
Arg2
Usp17l5
Gm13109
Gm5699
Gm4778
Gm4858
Gm5698
Gm5662
Col4a2
Col4a1
Sct
Cryab
Dkk1
Dab2
Gata6
Tmem132c
Dkkl1
Svop
Ptges
Flnc
Fbp2
Islr2
Vgll3
Cpeb2
Wls
Pros1
Phlda1
A430110N23Rik
Snai1
Matn1
Nkx2-9
Gprc5a
Lgals3
Zbtb10
Calcoco2
Ctgf
Cd63
Fabp3
Pmepa1
Atp6ap1
Mfge8
Tpp1
Mfsd1
Sdc4
Pltp
Perp
Mdm2
Ccng1
Btg2
Plk2
Anxa8
Cdkn1a
Eps8l2
Cd55
Chit1
1700007K13Rik
Cyr61
Tinagl1
Abcb1b
Lrpap1
Agpat9
Slc25a4
Wnt6
Cml2
Rtkn
Gm12800
Vnn1
Ltb4r2
Dub3
Ly6k
8030474K03Rik
Ddit4l
Igf2
Dub1a
Gm12794
Acad11
Zscan4d

Genes upregulated in Setdb1 KO, Kap1 KO and hnRNPK KD cells (33)

**Gene**	**BioGPS expression pattern**
Sdc4	Epidermis, Macrophage, Bone marrow
Pltp	Lung, Macrophage, Eye
Perp	Ubiquitous
Mdm2	Ubiquitous
Ccng1	Ubiquitous
Btg2	Ubiquitous
Plk2	M-1, Brain, Macrophage
Anxa8	Ubiquitous
Cdkn1a	Ubiquitous
Eps8l2	M-1, Intestine, Placenta
Cd55	Mast cells, Brain
Chit1	Epidermis, Stomach
1700007K13Rik	Testis, ESCs
Cyr61	M-1, Umbilical cord, Osteoblasts
Tinagl1	Placenta
Abcb1b	Adrenal gland, Ovary
Lrpap1	Kidney
Agpat9	Intestin, Adipose, B-cells
Slc25a4	Skeletal muscle, Adipose, Brain
Wnt6	Ovary, Bone marrow
Cml2	Liver
Rtkn	Salivary gland, Brain, ESCs
Gm12800	Unknown
Vnn1	Blastocyst, Kidney, Placenta
Ltb4r2	Cornea
Dub3	Unknown
Ly6k	Testis
8030474K03Rik	Neuro2a, testis, ESCs
Ddit4l	Ubiquitous
Igf2	Umbilical cord, Placenta, Osteoblasts
Dub1a	Ubiquitous
Gm12794	Ubiquitous
Acad11	Liver, Kidney, Adipose
Zscan4d	Unknown

Unknown = data not available
