# Supplementary material for: hnRNP K Coordinates Transcriptional Silencing by SETDB1 in Embryonic Stem Cells
Source: PLoS Genet. 2015 Jan 22;11(1):e1004933. doi: 10.1371/journal.pgen.1004933 (PMC4303303; doi:10.1371/journal.pgen.1004933)
Supplement: S3 Table — (PDF) [file pgen.1004933.s011.pdf]

ChIP Target	ChIP primer Name	Sequence (5'-3')
MLV 5' LTR-int	MLV +2	TGGGCAGGGGTCTCCAAATCT
	MLV -2	ATAAAGCCTCTTGCTGTTTGCATC
IAPEz 5' LTR-int	IAP-LTR-ChIP-s	GCTCCTGAAGATGTAAGCAATAAAG
	IAP-retro-ChIP-as	CTTCCTTGCGCCAGTCCCGAG
MMERVK10c 5' LTR-int	MMERVK10C_LTR_344-fw	CAAATAGCCCTACCATATGTCAG
	MMERVK10C_int_481-rv	GTATACTTTCTTCTTCAGGTCCAC
Etn 5' LTR-MusD int	MusD&Etn2	CCCTTCCTTCATAACTGGTGTCGCA
	MusD3&Etn	TAGCATCTCTCTGCCATTCTTCAGG
MERVL 5' LTR-int	MERVL_LTR-365-fw	CTTCCATTCACAGCTGCGACTG
	MERVL_int-519-rv	CTAGAACCACTCCTGGTACCAAC
MSCV 5' LTR-PBS	LTR 302	AACCATCAGATGTTTCCAGGGTG
	LTR -500	TTCGGATGCAAACAGCAAGAGGC
MSCV Gfp internal region	GFPcF	ACTACAACAGCCACAACGTCTATATCA
	GFPcR	GGCGGATCTTGAAGTTCACC
Ifna5 core promoter	Ifna5 TSS-F	AGAACCAAACAGCCCAGAAG
	Ifna5 TSS-R	TCA GGA AAG CAC AGA GCC TA
Myc core promoter	Myc TSS-F	CCTCCCGAGTTCCCAAAGC
	Myc TSS-R	CCT TCC CCA CCT CTC TCT ATT
Egr1 core promoter	Egr1 TSS-F	TTTCAAGGGTCTGGAACAGC
	Egr1 prom-R	GGAGGGCTTCACGTCACTC
Dazl core promoter	Dazl qPCR F2	TAC AAA ATG CCC GCA GAA ATA G
	Dazl qPCR R2	CCG GAC TCA ACC TTC TCA ATG
Fkbp6 core promoter	Fkbp6 prom-F	GTGGGATTCCGTTCCTGAG
	Fkbp6 prrom-R	CTATCGATCACGTGCCGTTT
Mael core promoter	Mael prom-F	GTGGGGAGAGCACTGTGATT
	Mael prom-R	GTT CGG GAA TGT GAA ACA CC
Taf7l core promoter-+1.5kb	Taf7l prom-F	CTCCCTCAGTGGGTGCTTC
	Taf7l prom-R	AGG CGA CGT GTC TCG TTA GT

qRT Target	qRT primer Name	Sequence (5'-3')
MLV RLTR4 MM-int	EndPro-	AGAGGTATGGTTGGAATAAGTA
	EndPro+	TAGATGGAGCCTACCAAGCTCTCAA
IAPEz 5' LTR-int	IAP-LTR-ChIP-s	Same as ChIP
	IAP-retro-ChIP-as	Same as ChIP
MMERVK10C 5' LTR-int	MMERVK10C_LTR_344-fw	Same as ChIP
	MMERVK10C_int_481-rv	Same as ChIP
Etn 5' LTR- MusD int	EtnI/II-MusD 29 up_fw	TCTTAATAATAGGGAGGAGATGTAGT
	EtnI/II-MusD LTR_123_rv	GGTCCAGTAGAAAGGTCGACG
MERVL 5'LTR-int	MERVL_LTR-365-fw	Same as ChIP
	MERVL_int-519-rv	Same as ChIP
MTA	MTA-F	ATGTTTTGGGGAGGACTGTG
	MTA-R	AGCCCCAGTTAACCAGAAC
Setdb1 exons 18-19	Setdb1 ex18-F	CTTCTGGCTCTGACGGTGATG
	Setdb1 ex18-R	GGAAGCCATGTTGGTTGATT

Hnrnpk exons 8-9	Hnrnpk ex8-F	ATCAGAGTCTGGCAGGAGGA
	Hnrnpk ex9-R	TCAGTAGAGTGAGGGCAGCA
Mcaf1 exons 11-12	Mcaf1 ex11-F	GACGATGAAGAGAGCGGAAC
	Mcaf1 ex12-R	CTGATGGCTGAAGAGGTGGT
Ubc9 exons 4-5	Ubc9 ex4-F	GATGGCACAATGAACCTGATGAAC
	Ubc9 ex5-R	TTTGGTGGTGAGGACGGATAGTC
Gata6 exons 3-4	Gata6 ex3-F	CCCTCATCAAGCCACAGAAG
	Gata6 ex4-R	CATATAGAGCCCGCAAGCAT
Gata3 exons 3-4	Gata3 ex3-F	TACCACCTATCCGCCCTATG
	Gata3 ex4-R	ACACACTCCCTGCCTTCTGT
Nkx2-9 exon 1	Nkx2-9 ex1-F	CCTCGCACTTCCATCCTTAG
	Nkx2-9 ex1-R	CTAGGCTTCGCATCCTGTTC
Dazl exon 4	Dazl ex4-F	GTGGCTTCTGCTCCACCTTCG
	Dazl ex4-R	CCTTGACTTGTGGTTGCTGA
Fkbp6 exons 3-4	Fkbp6 ex3-F	TACCTGGAGCACATGGACAA
	Fkbp6 ex4-R	ATAGGCTGGCTTGAACAGGA
Mael exons 11-12	Mael ex11-F	GGTTATTATCCATCTGGGGTGA
	Mael ex12-R	CATCTTTTTGGGGCGTGTAA
Taf7l exons 10-11	Taf7l ex10-F	TAGCCTCCATGAAGCAGACC
	Taf7l ex11-R	TTGAGGGTCAAGTTTTCCACTT
Egr1 exons 1-2	Egr1 ex1-F	GAGCGAACAACCCTATGAGC
	Egr1 ex2-R	TTTGGCTGGGATAACTCGTC
Gapdh exons 4-5	Gapdh ex4-F	GTG CTG AGT ATG TCG TGG AG
	Gapdh ex5-R	GAG ATG ATG ACC CTT TTG GCT C
B-actin exons 2-3	b-actin-ex2-fw	GAACCCTAAGGCCAACCGTG
	b-actin-ex3-rv	GGAGTCCATCACAATGCCTG
